# In and out: *Leishmania* metastasis by hijacking lymphatic system and migrating immune cells

**DOI:** 10.3389/fcimb.2022.941860

**Published:** 2022-08-12

**Authors:** Baijayanti Jha, Marta Reverte, Catherine Ronet, Florence Prevel, Florence D. Morgenthaler, Chantal Desponds, Lon-Fye Lye, Katherine L. Owens, Leonardo Scarpellino, Lalit Kumar Dubey, Amélie Sabine, Tatiana V. Petrova, Sanjiv A. Luther, Stephen M. Beverley, Nicolas Fasel

**Affiliations:** ^1^ Department of Immunobiology, University of Lausanne, Epalinges, Switzerland; ^2^ Department of Oncology, Ludwig Institute for Cancer Research Lausanne, Lausanne University Hospital and University of Lausanne, Lausanne, Switzerland; ^3^ Cellular Imaging Facility, University of Lausanne, Epalinges, Switzerland; ^4^ Department of Molecular Microbiology, School of Medicine, Washington University, St. Louis, MO, United States; ^5^ Centre for Microvascular Research, John Vane Science Centre, Queen Mary University of London, London, United Kingdom; ^6^ Department of Oncology and Ludwig Institute for Cancer Research, University of Lausanne and Centre Hospitalier Universitaire Vaudois, Epalinges, Switzerland

**Keywords:** lymph nodes (LNs), inflammation, dissemination, *Leishmania*, *Leishmania* RNA virus 1 (LRV1), metastasis, extracellular, free amastigotes

## Abstract

The lymphatic system plays a crucial role in mounting immune response against intracellular pathogens, and recent studies have documented its role in facilitating tumor dissemination linked largely with cancer cells. However, in mucocutaneous leishmaniasis (MCL) caused by *Leishmania Viannia* subgenus showing infectious metastasis and resulting in severe distant secondary lesions, the route of escape of these parasites to secondary sites has not yet been investigated in detail. Our results demonstrated that when infection was associated with inflammation and additionally exacerbated by the presence of dsRNA viral endosymbiont (LRV1), lymphatic vessels could serve as efficient routes for infected cells to egress from the primary site and colonize distant organs. We challenged this hypothesis by using the intracellular *Leishmania* protozoan parasites *Leishmania guyanensis (Lgy)* associated with or without a dsRNA viral endosymbiont, exacerbating the infection and responsible for a strong inflammatory response, and favoring metastasis of the infection. We analyzed possible cargo cells and the routes of dissemination through flow cytometry, histological analysis, and *in vivo* imaging in our metastatic model to show that parasites disseminated not only intracellularly but also as free extracellular parasites using migrating immune cells, lymph nodes (LNs), and lymph vessels, and followed intricate connections of draining and non-draining lymph node to finally end up in the blood and in distant skin, causing new lesions.

## Introduction

Cutaneous leishmaniasis (CL) affects more than 1 million people across the globe annually, being the most common clinical form of leishmaniasis ((W.H.O), 2020). It manifests itself as self-healing skin lesions at the inoculation site of *Leishmania* parasites, which enter the human skin through the bite of a hematophagous female sand fly (Wong, 1995; Bates, 2008). Upon delivery into mammalian hosts, free-living flagellated promastigotes pass through neutrophils and invade macrophages, wherein they transform into a non-flagellated amastigote form to survive intracellularly in the phagolysosome-like organelles (Wong, 1995; Novais et al., 2009; Wynn et al., 2013; Carlsen et al., 2015; Bates, 2018). As mentioned, these protozoan parasites mainly cause cutaneous leishmaniasis (CL), but with some species, such as *Leishmania braziliensis (Lbr)* and *Leishmania guyanensis* (*Lgy*), CL primary lesions can progress to mucocutaneous leishmaniasis (MCL), a chronic inflammatory form with severe secondary lesions and destruction of nasopharyngeal tissues (Lessa et al., 2007; Hartley et al., 2014)*. Lbr* and *Lgy* mainly account for most CL cases in South America, with approximately 5%–10% of such cases progressing to the more severe chronic form of the disease, resulting in mucocutaneous leishmaniasis (MCL), wherein parasites metastasize and cause severe secondary cutaneous lesions. Disseminated cutaneous leishmaniasis (DCL) is another form marked by hyper-inflammation, appearance of nodular granulomas, and numerous ulcerated skin lesions exhibiting extensive parasite migration (Hartley et al., 2012; Hartley et al., 2014). These intense symptomatic manifestations associated with several *Leishmania* species infections could be linked to the presence of *Leishmania* RNA virus 1 (LRV1) from the *Totiviridae* family, an endosymbiotic double-stranded RNA virus (dsRNA) reported to be naturally present in the cytoplasm of several *Leishmania* species, along with several other parasite species as well (Tarr et al., 1988; Ives et al., 2011; Zangger et al., 2014; Cantanhede et al., 2015).

LRV1 is a potent immunogen, recognized by endosomal TLR-3 (the dsRNA sensor) in macrophages, inducing an extensive type I interferon (IFN-I) anti-viral immune response, thus leading to a hyper-inflammatory phenotype (Ives et al., 2011; Zangger et al., 2013; Heirwegh et al., 2021). Additionally, co-infection of mice already infected with *Lgy*LRV1− with lymphocytic choriomeningitis virus (LCMV) reproduced the metastatic phenotype observed in *Lgy*LRV1+ infections, confirming the severity rendered by the LRV1 virus or other co-infecting viruses (Rossi et al., 2017; Rath et al., 2019). *Lgy*LRV1+ infections result in metastatic complications which are often associated with treatment failure and clinical relapse in patients (Adaui et al., 2016; Bourreau et al., 2016; Olivier and Zamboni, 2020). However, the mode of dissemination and the cells facilitating such extensive dissemination to distant skin leading to the formation of new metastatic lesions have not been thoroughly investigated yet. Therefore, there is an urgent need to identify the process of dissemination and potential major cargo cell type(s) transporting viable parasites from the primary site of infection to distant secondary debilitating lesion sites, for targeted therapeutic intervention to ensure the control of this disease. In this regard, the lymphatic system is of significant interest, as it is known to play key roles in the development of immune responses and has also been described to be involved in the dissemination of tumor cells to both draining lymph nodes and distant secondary sites. (Petrova and Koh, 2020; Vaahtomeri and Alitalo, 2020). Furthermore, various innate and adaptive cells of the immune system are reported to be involved in a complex interplay with each other, in different species of *Leishmania* infections, to house an effective host immune response against an invading pathogen, thereby modulating the susceptibility or resistance to such infections (Qi et al., 2001; Charmoy et al., 2010; Santos Cda et al., 2013; Yang et al., 2014; Cardoso et al., 2015; Silva-Barrios et al., 2016; Glennie et al., 2017; Novais et al., 2017; Tomiotto-Pellissier et al., 2018).

Experimentally, metastatic CL can be mimicked upon infection of the interferon gamma (IFN-γ)-deficient mice (*Ifng^−/−^*) with *Lgy*-bearing LRV1 (*Lgy*LRV1+), which induces a severe metastatic phenotype (Hartley et al., 2016; Rossi et al., 2017). This metastatic mouse model is now being used to study the dissemination of the infection to secondary sites, since it validates the observation that *Lgy*LRV1+-infected patients have a high level of IL-17 but extremely low level of interferon gamma cytokine (IFN-γ) and are prone to chronic metastatic complications (Hartley et al., 2016). The model thus presents a simple yet extremely useful system for defining the different immunological determinant(s) driving leishmanial metastasis *in vivo*. Thus, in this study, we used *Lgy* parasites that may or may not be infected by LRV1, namely, *Lgy*LRV1+ and *Lgy*LRV1−, respectively, and an *in vivo Ifng^−/−^* mice model exhibiting infectious metastasis to investigate the mode of dissemination of *Lgy* parasites and the potential cargo cells facilitating widespread metastasis.

## Materials and methods

### Ethics statement

All mice experiments and animal protocols undertaken in this study were approved by the Swiss Federal Veterinary Office (SFVO), under authorization number VD 3551. Animal handling and experimental procedures were conducted with strict adherence to the ethical guidelines set by the State Ethical Committee and the SFVO for the use of laboratory animals, with regular inspection by the Department of Security and Environment of the State of Vaud, Switzerland. We managed to follow maximum experiments adhering to the set ARRIVE rules for handling laboratory animals, with proper control of animal maintenance conditions, regular behavior check, and administration of pain-relieving drugs (paracetamol/dafalgan) from the peak of infection. All these have been discussed in details in *Materials and methods*.

### Laboratory animals

WT (C57BL/6) mice were purchased from Envigo (Netherlands). *Ifng^−/−^* mice and *Rag2^−/−yc−/−^* transgenic mice were purchased from The Jackson Laboratory (United States). We used both male and female mice for randomization in the experimental setup, with age ranging between 8 and 16 weeks for these mice groups and median age of 12 weeks for infections and controls. Animals were treated randomly before intervention to maximize randomization and reduce bias. No wild animals were used in this study. No field-collected samples were used in this study. Different mice strains were genotyped by PCR, using KAPA Mouse Genotyping Kit (KAPA Biosystems) on tissue-isolated genomic DNA, as per provider’s protocol for screening. The oligonucleotides used for genotyping were the following: *Ifng* mutant Fw, CCTTCTATCGCC TTCTTGACG; *Ifng* WT Fw, AGAAGTAAGTGGAAGGGCCCAGAAG; and *Ifng* common, AGGGAAACTGGGAGAGGAGAAATAT. All mice were maintained under a specific pathogen-free (SPF) environment, housed in micro-isolator cages, for housing, breeding, and future maintenance of the line at the animal facility of the Center of Immunity and Immunology, Lausanne, (Switzerland). Mice experiments were performed in a P2 pathogen animal facility on site (as mentioned). Food (SAFE or KLIBA NAFAG) and water (filtered local water, which is autoclaved, after acidification or Innovive Aquavive) were provided *ad libitum*. The animal facility maintained a prescribed light cycle with 11 h of darkness and 13 h of light; temperature and humidity were set at 21°C ± 2 and 55% ± 10, respectively.

### Parasites and their culture

Two isogenic clones of *Lgy*, either infected with or depleted of LRV1−, namely, *Lgy*LRV1+ [LRV1^+^ *Lgy*M4147/SSU : IR2SAT-LUC(b)c3] and *Lgy*LRV1− [LRV1^−^ *Lgy*M4147/SSU : IR2SAT-LUC(b)c3], respectively, were used in this study. Both of these clones were derived from the LRV1+ parent strain, *Lgy* M4147 (MHOM/BR/75/M4147), and documented previously (Kuhlmann et al., 2017). These parasites express equivalent levels of a firefly luciferase gene, namely, “ffLUC” (5×10^7^ photons/s/10^6^ parasites), which in turn is stably integrated into the small subunit (SSU) gene of the ribosomal RNA locus (Hartley et al., 2016). Additionally, we have also used a fluorescent variant of the same parasite, namely, mCh*Lgy*LRV1+ (*Lgy*M4147 0106-9 L+ SSU : IR4BSD-LUC-mcherryc8), which has the mCherry gene integrated into the SSU, also expressing ffLUC simultaneously (Reverte et al., 2021). We also used the *Lmj* (MRHO/IR/75/ER (IR75) strain for additional controls (Noll et al., 1997; Reverte et al., 2021). *Lgy* parasites were cultured in Schneider’s *Drosophila* medium (PAN™ BIOTECH) supplemented with 20% heat-inactivated fetal bovine serum (FBS, Gibco™), 1% penicillin-streptomycin (P/S) solution (Bio-Concept), 1% HEPES buffer, 0.1% hemin-folate solution (Sigma-Aldrich, Fluka), and 0.6 µg/ml of 6-Biopterin (Sigma-Aldrich). Similarly, *Lmj* parasites were cultured in Medium 199 (M199, Gibco) supplemented with 20% heat-inactivated FBS, 1% P/S solution, 1% HEPES buffer, 0.1% hemin-folate solution, and 0.6 µg/ml of 6-biopterin. Generally, these parasites were maintained as promastigotes in culture *in vitro*, at 26°C and 5% CO_2_, for not more than five passages, and isolated and cultured from the WT-infected mice FP *in vivo*, to maintain their virulence, for long-term maintenance of parasite stocks. Each passage yielded stationary-phase, infectious metacyclic promastigotes after 6 days in culture, which were used directly for *in vivo* infections, diluted in Dulbecco’s phosphate-buffered saline (DPBS, Gibco) at required concentrations specified later.

### Reagents

*Antibodies.* The antibodies used in this study for various applications such as FACS, immunofluorescence/histological staining, and *in vivo* mice depletions are detailed in **Supplementary Tables S1** and **S2**.

### Mice infection and *in vivo* bioluminescence imaging and quantification

Age-matched (between 6 and 12 weeks) male and female mice were used separately for individual experiments to remove gender bias in this study. Mice were mostly infected with 1×10^6^ *Lgy* (either *Lgy*LRV1+ or *Lgy*LRV1−) stationary-phase, infectious metacyclic parasites in both hind FP subcutaneously, in 50 µl of 1× DPBS, as a fixed standard for the entire study. Following infection, changes in footpad thickness were measured weekly using a Vernier caliper, as a proxy for parasite growth and disease progression. Parasite burden was quantified in the infected mice by injecting VivoGlo D-Luciferin salt (Promega) at a concentration of 150 mg/kg, intraperitoneally (i.p.), and parasite bioluminescence produced in the mouse footpads and other visible secondary lesions (like tail and snout) were measured with *In-Vivo* Xtreme II (BRUKER) as previously described (Reverte and Fasel, 2019). Additionally, *in vivo* read out of inflammation was measured similarly by injecting Luminol sodium salt (Carbosynth) at a concentration of 200 mg/kg i.p. The infected experiment groups were imaged twice at 10 and 20 min, respectively, during late phase of chronic infection for better uptake of imaging salt in a highly metastatic condition. These acquired images were then analyzed using Molecular Imaging (MI) software (BRUKER), setting specific regions of interest (ROIs) on FP, tail, etc. and expressing the measured bioluminescence signals in units of photons per second (p/s) (Reverte and Fasel, 2019). Immunocompromised mice (*Ifng^−/−^*) received constant pain-relieving medication [1g/L DAFALGAN (Upsa) diluted in drinking water], from the appearance of big primary lesions (W3–4 p.i.) till secondary lesions developed (till a maximum of W12 p.i., in case of delays) and were euthanized upon reaching any ethical limit of permitted lesion size or showing visual signs of clinical disease such as ruffled fur, inactivity, labored respiration, huddling behavior, or a loss of 20% of their original body weight.

### Lymph node and lymphatic connection mapping

Groups of age-matched (6–12 weeks) male or female *Ifng^−/−^* mice were used as untreated, uninfected naive controls, alongside infected groups with *Lgy*LRV1+ parasites, at different time points (i.e., weeks 1, 2, 4, 6, 8–10) post-infection, for *in vivo* lymph node mapping using Evan’s blue, as described previously (Harrell et al., 2008). Dye injections were administered with 5% Evan’s Blue dye (Sigma) in 10–15 μl Hank’s buffered salt solution (Gibco), delivered using a precision syringe (Hamilton, 50 μl) with a stainless-steel-made sharp, beveled RN needle (Hamilton, Germany) (Harrell et al., 2008). The dye was injected subcutaneously into both hind FP of naive and infected mice group, and subjects were anesthetized under isoflurane (2.5%) for a continuous 20 min to facilitate dye uptake by the lymphatic vessels. Then, mice were euthanized with CO_2_ and dissected to locate the blue-labeled LNs of interest to assess the drained (blue) and non-drained (non-blue) LNs post-FP injection (equivalent to our infection model). While it was easier to detect the peripheral draining LNs by blue color, just after the removal of skin and fascia, deep-seated LNs like the iliac and renal LNs were visible only when the intestines were removed. Moreover, once the draining and non-draining LNs were identified, the pattern of lymphatic connection between these various LNs were mapped by injecting 2–5 µl (10 mg/ml in 1× DPBS) of the fluorescent permeability tracer fluorescein-isothiocyanate (FITC)-labeled dextran (2,000 kDa; Sigma-Aldrich) (Zheng et al., 2014; Yamaji et al., 2018) subcutaneously in the hind FP, the tip of the tail, and forelimbs of the infected mice group (*Ifng* ^−/−^ *Lgy*LRV1+), as described previously (Zheng et al., 2014). The apparatus and procedure of euthanasia is exactly as described for the Evan’s blue mapping. Once euthanized, the mice were micro-dissected and imaged at 8× magnification (in both setups of bright light and fluorescence imaging) for 30 min with a stereomicroscope (Leica, M205FA), and acquired images were processed using LAS AF 6000 software and were processed, tiled, and stitched on Photoshop (version 21.2.0) in case of fluorescence-based images of the whole mouse, as represented in **Figure 5**.

### Isolation of cells (from LNs, tissues, and blood) and cell count

Samples (LNs, liver, lung, kidney, heart, spleen, etc.) were mechanically processed using a McIlwain tissue homogenizer (Mickle Laboratory Engineering). They were then digested in a solution of incomplete Dulbecco’s modified Eagle’s medium (DMEM, Gibco™, without FBS) supplemented with 1 mg/ml collagenase type A (Roche, 1:100 dilution) and 25 mg/ml DNase (Sigma, 1:500 dilution), at 35°C for 15–30 min (depending upon organ size and type). For dense tissues such as FP and tail, the skin was peeled off, and tissue/lesions were separated from the bones, then processed as described, after digestion for 1 h (Regli et al., 2020). Enzyme activity was neutralized by the addition of cold complete DMEM [incomplete medium: DMEM (Gibco™), supplemented with 10% heat-inactivated fetal bovine serum (FBS), 1% penicillin/streptomycin, and 1% HEPES (Sigma-Aldrich)]. Cell suspension was dispersed through a 40-µm cell strainer (Falcon) to remove cell clumps and centrifuged at 500*g* for 10 min to obtain purified single-cell pellet, which may or may not be treated with erythrocyte lysis buffer (BD FACS™ Lysing Solution) to remove red blood cells. Meanwhile, blood samples (from infected mice and naive control) were collected in tubes containing 400 μl PBS-heparin [(Sigma-Aldrich H3149, 10,000× from (10 mg/ml)] on ice, centrifuged at 500*g* for 10 min to remove the PBS-heparin, treated immediately with 3–5ml of BD FACS™ Lysing Solution (1:10 diluted in deionized water from stock) for 10 min at room temperature (RT) in the dark, then washed twice with 1× DPBS and centrifuged to obtain a cleaner red blood cell (RBC)-free blood cell pellet (Regli et al., 2020).

*Cell count*. Once the cell suspension was prepared for each LN and tissue, cell numbers and cell viability were assessed *via* Trypan blue (Sigma-Aldrich) exclusion using an improved counting Neubauer chamber (Assistant^®^, catalog number 40442). This gave the absolute cell count of different organs.

### Limiting dilution assay/analysis

Different LNs (PLN, ILN, iliac LN, ALN, BLN, CLN, MLN, and organs/tissues like spleen, FP, and tail) (divided into eight pieces of equal length, from the thick to the thin end, marked from T1 till T8, respectively), kidney, liver, lung, heart, and even blood were aseptically collected from infected and/or naive control groups at different time points (weeks 1, 2, 4, 6, 8–10) post-infection in cold incomplete DMEM (Gibco) on ice and further processed to obtain single-cell suspension and their absolute cell count per organ (as described in detail, previously). Then, an eightfold limiting dilution assay (in successive dilution series of 1/1, 1/2, 1/4, 1/8, 1/10, 1/100, 1/1,000, and 1/10,000 by row) with 24 technical replicates per dilution was performed on these cell suspensions in a 96-well U bottom transparent plate (Fisher Scientific) in complete Schneider’s medium. These plates were then cultured at 26°C for 14–21 days, and subsequent readings were taken at 3, 6, 9, 14, 18, and 21 days in culture to monitor the presence of any *Lgy* promastigotes through microscopy. This was used to identify the infected organs at different time points of infection between different infected and control groups. Infection was annotated by a + sign for the different kinds of LNs and organs (as mentioned by abbreviations). The number of + assigned to any organ correlated to the detection of free parasites in equivalent progression of the dilution series, such that 1/1, 1/2, 1/4, 1/8, 1/10, 1/100, 1/1,000, and 1/10,000 translates to +, ++, +++, ++++, +++++, ++++++, +++++++, and ++++++++, respectively (only when minimum 12 out of 24 technical replicates showed free promastigotes). The parasite number was determined from the lowest cell concentration from which promastigotes could be grown using the ESTIMFRE software, which is based on the Poisson limit theorem as previously described (Regli et al., 2020). The corresponding heatmaps were generated using the R ComplexHeatmap package. The color gradient represented in the heatmaps is correlated to the number of + signs assigned in terms of infection in progressive dilution series through LDA for each organ, where increased color intensity corresponded to higher parasite load.

### RNA isolation and RT-qPCR

LNs, FP, and tail pieces were collected from *Ifng* ^−/−^ mice, infected with either *Lgy*LRV1+ or *Lgy*LRV1− parasites, at weeks 1, 2, 4, 6, 8–10 p.i, and snap-frozen in liquid nitrogen. RNA isolation from these samples were facilitated by immersing them in TRI Reagent^®^ (Molecular Research Center, Inc.) in RNAse-free tubes, adding stainless beads (Qiagen), and using Tissue Lyser system (Qiagen) for tissue disruption and homogenization. RNA was isolated through chloroform/isopropanol/ethanol phase separation, as described previously (Henn et al., 2018), following the manufacturer’s description. The quality and quantity of isolated RNA were analyzed using NanoDrop™ 2000 (Thermo Fisher Scientific). cDNA was generated using SuperScript II Reverse Transcriptase (Invitrogen). LightCycler^®^ 480 SYBR Green I Master (Roche) along with 0.5 µM primer pairs were used for performing real-time quantitative PCR (RT-qPCR) on LightCycler^®^ 480 (Roche). Gene expression was analyzed using the threshold cycle (C_T_) method 2^−ΔΔCt^. Data analyzed for *Leishmania Kmp11* gene expression in different organs of the infected mice were assessed using predetermined levels of *Kmp11* expression in *Lgy* parasites and normalized to the total RNA quantity of the sample (Hartley et al., 2016; Reverte et al., 2021). Primer sequence used for *Kmp11* gene were as follows: *Kmp11* Fw, GCCTGGATGAGGAGTTCAACA, and *Kmp11* Rev, GTGCTCCTTCATCTCGGG.

### Immunofluorescence microscopy

Draining and non-draining LNs were carefully dissected as a whole, harvested, weighed, and fixed in 4% (wt/vol) paraformaldehyde (Fluka) in 1× DPBS at 4°C overnight (O/N), then saturated in 30% (wt/vol) sucrose (Fisher Scientific) in 1× DPBS again O/N at 4°C, before being embedded in Tissue-Tek optimum cutting temperature (OCT) compound (Sakura), and frozen in an ethanol dry ice bath. Serial longitudinal and transverse cryostat sections (8–10 μm in thickness) were collected on Superfrost/Plus glass slides (Fisher Scientific), over a span of 400 μm depth. These sections were then air-dried, fixed in ice-cold acetone for 20 min, and then rehydrated in 1× DPBS and were blocked with 1% (wt/vol) BSA (Sigma-Aldrich) supplemented with 1% normal mouse and 4% donkey serum (BIO-RAD). Various primary antibodies, in different combinations, diluted in 1× DPBS containing 1% (vol/vol) normal mouse serum and 1% (wt/vol) BSA, were added for specific immunofluorescence staining. Then, these sections were incubated O/N at 4°C. The following day, cryosections were washed at least three times in 1× DPBS, then incubated with specific fluorescently labeled secondary antibodies to detect the primary antibodies. For gp38, staining was revealed using horseradish peroxidase (HRP)-conjugated secondary reagents followed by tyramide signal amplification (Molecular Probes Kit 22) according to the manufacturer’s instructions, but using a borate buffer (0.1 M in PBS; pH 8.5) for tyramide dilution. Moreover, rabbit anti-mCherry (Abcam) secondary antibody was used to detect the mCh*Lgy*LRV1+ in these infected samples, as the self-fluorescence of these parasites were quenched with 4% paraformaldehyde (PFA) fixation. Prior to mounting, sections were counter-stained for nuclei with 4′,6-diamidine-2′-phenylindole dihydrochloride (DAPI) from Molecular Probes, using ProLong anti-fade reagents (Life technologies). Stained cryo-sections were then imaged within 24–72 h post-staining and stored at 4°C in polystyrene slide box for future considerations. A detailed list of antibodies used for this study are enlisted in **Supplementary Tables S1** and **S2**. Images were partly acquired using on an Olympus VS120-SL full slide scanner with a 20×/0.75 air objective (using Olympus OlyVIA software), at the EPFL BioImaging & Optics Platform (BIOP), or with Zeiss Axio-Imager Z1, Upright in Cellular Imaging Facility, Epalinges (using Axiovision SE64 rel 4.9.1 software). For the images acquired using the Olympus VS120-SL full slide scanner, individual images were acquired using the indicated fluorescent channels having the same exposure time employed across different samples. The images were then extracted using the VSI reader action bar [provided by the EPFL BioImaging & Optics Platform (BIOP)]. Olympus slide scanner software (OlyVIA v.2.6) was employed to directly analyze the images from different groups (naive and infected LNs) by adjusting the contrast and brightness settings, so that they are constant across different compared samples and groups, as described in detail previously (Dubey et al., 2019).

### Fluorescent-activated cell sorting

All the antibody dyes and beads used for flow cytometry are detailed in **Supplementary Table S1**.

For flow cytometric analysis, different LNs, tissues, and blood (mentioned previously) were recovered from naive controls and *Ifng* ^−/−^ mCh*Lgy*LRV1+ (at different time points of infection); single-cell suspensions with respective cell counts from each organ were obtained following the procedure described in detail earlier. Briefly, in 96-well U-bottom plates, 2×10^6^ cells from each organ of interest were treated for further 5 min with 5 mM EDTA in fluorescent-activated cell sorting (FACS) buffer (1× DPBS with 2% FBS) to dissociate cell clumps, then incubated with 50 μl supernatants from hybridoma 2.4G2 antibody (Miltenyi Biotec; 1g anti-FcRIII/II antibody per 10^6^ cells) on ice for 20 min to block Fc receptors. Cells were then stained with optimal concentrations of specific antibodies (between 0.06 and 0.5 μg/1×10^6^ cells in 50 μl of FACS buffer) in appropriate combinations, for the identification of different mouse antigens, employing fluorescein phycoerythrin (PE) or allophycocyanin or PE-Cy7, isothiocyanate (FITC), Brilliant Violet (BV421)/(BV510), APC or APC/Cy7 conjugated, or biotinylated monoclonal antibodies to CD45 (clone 104), CD11b (clone M1/70), Gr-1 (clone RB6-8C5), Ly6C (clone HK1.4), Ly6G (clone 1A8), CCR2 (clone SA203G11), CD206 (clone C068C2), MHC class II (clone M5/114.15.2), CD115 (clone T38-320), F4/80 (clone BM8), CD3e (clone 17A2), CD19 (clone D3/CD19), NK1.1 (clone PK136), and CD11c (clone N418). Plates were then incubated for 30 min at 4°C in the dark. Following incubation, the cells were washed at least three times with FACS buffer. DAPI (Molecular Probes, 1:1,000 from a stock of 10 mg/ml) was added to each sample during staining to exclude dead cells during analysis. Murine cells and fluorescent or stained parasites were then passed through the flow cytometry analyzer of either BD LSRII or BD LSR-Fortessa series (Becton Dickinson), and a minimum of 50,000 live cells were acquired (Rossi et al., 2017; Regli et al., 2020). Data were analyzed with FlowJo software (Tree Star, v10.0.6). For data analyses, a minimum of 30,000 events (cells) were evaluated at all times by back-gating from CD45+/− stained cells. The absolute number of total leukocytes or each represented cell type was quantified by multiplying the total number of cells observed by hemocytometer counting with the percentage of those individual cells (on total CD45^+^ cells) determined by flow cytometry. The absolute number of each leukocyte subset (1A8, Ly6C, Ly6G, CD11b, and CCR2) was determined by multiplying the percentage of each gated population by the total number of CD45^+^ cells. For the distribution of mCherry^+^ parasites on cell-associated or non-immune cell-associated TER119^−^CD45^+^ immune cells, we calculated it from the point of view of the mCherry^+^ parasites, distributing the total numbers of mCherry^+^ parasites observed in any organ over its numbers that were either co-localized with a TER119^−^CD45^+^ membrane marker (thus, cell associated) or not (non-cell associated).

### Imaging flow cytometry (Imaging Stream^®^ analysis)

All the antibody dyes and beads used for imaging flow cytometry are detailed in **Supplementary Table S1**.

Samples were run in a two-camera, 12-channel Image-StreamX multispectral imaging flow cytometer (Amnis, Luminex Corporation) at low speed and highest magnification (60×). Instrument setup and performance tracking was performed daily using the Amnis^®^ SpeedBead^®^ Kit (Luminex Corporation) to verify optimal instrument performance. Cells were excited using a 405-nm laser (25 mW), a 488-nm laser (100 mW), a 561-nm yellow laser (200 mW), and a 642-nm red laser (150 mW). Only events with a brightfield area >5 µm^2^ (to exclude cell debris) and non-saturating pixels were collected (as described in Hui et al., 2017). Data were acquired for a minimum of 50,000 events/sample (Regli et al., 2020).

Panel design was based on antigen expression density, fluorochrome brightness, and reagent availability in each panel for the four-laser, 12-parameter ImageStreamX. The preparation of cell suspension and staining were done exactly as described previously for regular flow-cytometric analysis and passed freshly in two panels (Panels 1 and 2), as mentioned. Panel 1 experimental samples contained images and data for brightfield (channels 1 and 9), Ly6C-FITC (channel 2), Ly6G-PE (channel 3), mCherry (for mCh*Lgy*LRV1+ parasites) (channel 4), CD11b-PECy5 (channel 5), CD206-biotin revealed with SAV-PECy7 (channel 6), DAPI (channel 7), CD45.2-BV510 (channel 8), CCR2-APC (channel 11), and F4/80-APC/Cy7 (channel 12). Panel 2 experimental samples contained images and data for brightfield (channels 1 and 9), CD19-FITC (channel 2), NK 1.1-PE (channel 3), mCherry (for mCh*Lgy*LRV1+ parasites) (channel 4), CD3-PECy5 (channel 5), CD45-PECy7 (channel 6), DAPI (channel 7), MHC II-BV510 (channel 8), CD11c-APC (channel 11), and side scatter (SSC, channel 12). Single-color controls for both panels were acquired to generate the compensation matrixes, which were applied to each panel, respectively, prior to analysis using IDEAS (Image Data Exploration and Analysis Software) 6.2 software (Amnis Corporation). Cell internalization versus non-internalization, free or sticking to the cell surface or being in clumps for parasites, was defined using the internalization of the bright parasite spots within the membrane marker mask. Calculations and analyses of cell the population were done exactly as defined under FACS studies.

### Cell depletion in mice

All the antibody dyes and beads used for cell depletions are detailed in **Supplementary Table S2**.

To study the individual effect of different cell types on disease progress and outcome, the infected model of *Ifng* ^−/−^ with fluorescent parasites mCh*Lgy*LRV1+ mice (as established and documented several times previously) was subjected to three different sets of major cell depletion *in vivo*, using defined depletion antibodies (all from BioXcell) as follows:

Neutrophil depletion: To deplete neutrophils, *Ifng* ^−/−^ mice were injected with purified *InVivo*MAb anti-mouse Ly6G (Clone 1A8, BioXcell) (Moynihan et al., 2016; Davis Iv et al., 2019) at a dose of 0.2 mg/200 μl (in 1× DPBS) i.p., twice at 24 and 6 h, before the point of infection with *Lgy* parasites and every 48 h thereafter, continued with an equivalent dose till 2 weeks post-infection. Control groups received similar doses of purified whole rat IgG [*InVivo*MAb rat IgG2a isotype control; anti-trinitrophenol (clone 2A3)] in the same timeline as the recommended control for the depletion.

Simultaneous neutrophil and monocyte depletion [with *InVivo*MAb anti-mouse Ly6G/Ly6C (Gr-1), clone RB6-8C5, BioXcell] (Bodogai et al., 2015; Bansal et al., 2018): to facilitate GR-1+ myeloid cell depletion *in vivo*, anti-mouse Ly6G/Ly6C(Gr-1) mAb at a dose of 0.3mg/200 μl (in 1× DPBS) was administered i.p. in *Ifng* ^−/−^ mice, daily for 3 days prior to infection, and then continued every 48 h thereafter, with an equivalent dose till 2 weeks post-infection. Control mice received similar doses of normal rat IgG2b isotype control [*InVivo*MAb, anti-keyhole limpet hemocyanin, (LTF-2), Bioxcell] as recommended control.

Macrophage depletion [with *InVivo*MAb anti-mouse CSF1R (CD115), clone AFS989, BioXcell] (Gordon et al., 2017; Bauché et al., 2018): *Ifng* ^−/−^ mice were injected i.p. with anti-CSF-1R mAb (AFS98) keeping rat IgG1 isotype control (rat IgG2a isotype control; anti-trinitrophenol was clone 2A3), at doses of 0.4 mg/200 μl (in 1× DPBS) daily for 4 days from point of infection and then continued every 48 h thereafter, with an equivalent dose till 2 weeks post-infection.

Additionally, peripheral blood smears (randomly selected from the subjects of each depletion group) were examined on day 0 and weekly thereafter, for the duration of the neutrophil and RB6-8C5 depletion period. Briefly, blood was collected from the submandibular vein, smeared onto a slide, and allowed to dry. Diff-Quick was used to stain the blood smears, and at least 200 nucleated cells per slide were counted by an individual experienced in counting blood differentials. Percentages of specific cell types were determined from the total number of cells counted.

### Validation of antibodies

All antibodies used in this study for different purposes enlisted above are commercially available. See the corresponding manufacturer data sheets and cited references for use (on respective webpages) for further validation and protocol for individual antibodies. They further provide information about large-scale use and validation through defined means. We followed exactly similar protocols, which were defined in close context to our kind of mice studies. All antibodies were validated before use, and the information could be made available upon further request from the corresponding author.

Further information could be found by logging into the following major sites and providing the specific information about each of the antibodies enlisted for these studies:

For BioLegend antibodies: https://www.biolegend.com/

For eBioscience antibodies: https://www.thermofisher.com/ch/en/home/lifescience/antibodies/ebioscience.html

For BioXcell antibodies: https://bxcell.com/

For R& D systems antibodies: https://www.rndsystems.com/

For anti-mCherry mouse antibody from Abcam: https://www.abcam.com/mcherry-antibody-ab167453.html

### Instruments for data collection

RNA isolation mice samples were facilitated by immersing them in TRI Reagent^®^ (Molecular Research Center, Inc.) in RNAse-free tubes, adding stainless beads (Qiagen) and using Tissue Lyser system (Qiagen) for tissue disruption and homogenization. RNA was isolated through chloroform/isopropanol/ethanol phase separation, following the manufacturer’s description. The quality and quantity of isolated RNA were analyzed using NanoDrop™ 2000 (Thermo Fisher Scientific). cDNA was generated using SuperScript II Reverse Transcriptase (Invitrogen). LightCycler^®^ 480 SYBR Green I Master (Roche) along with 0.5 µM primer pairs was used for real-time quantitative PCR (RT-qPCR) on LightCycler^®^ 480.

For bright light and fluorescent lymphangiography, euthanized mice were micro-dissected and imaged at 8× magnification (in both setup) for 30 min with a stereomicroscope (Leica, M205FA), and the acquired images were processed using LAS AF 6000 software.

Flow cytometry data were collected using BD LSRII or the BD LSR-Fortessa series (Becton Dickinson), and a minimum of 50,000 live cells were acquired for each set of analysis. Additionally, for imaging flow cytometry, samples were run in a two-camera, 12-channel Image-StreamX multispectral imaging flow cytometer (Amnis, Luminex Corporation) at low speed and highest magnification (60×). Instrument setup and performance tracking were performed daily using the Amnis^®^ SpeedBead^®^ Kit (Luminex Corporation) to verify optimal instrument performance. Cells were excited using a 405-nm laser (25 mW), a 488-nm laser (100 mW), a 561-nm yellow laser (200 mW), and a 642-nm red laser (150 mW). Only events with a brightfield area >5 µm^2^ (to exclude cell debris) and non-saturating pixels were collected. Data were acquired for a minimum of 50,000 events/sample. Moreover, panel design was based on antigen expression density, fluorochrome brightness, and reagent availability in each panel for the four-laser, 12-parameter ImageStreamX. Preparation of cell suspension and staining were done exactly as described for regular flow-cytometric analysis and passed freshly in two panels (Panels 1 and 2) as mentioned. Panel 1 experimental samples contained images and data for brightfield (channels 1 and 9), Ly6C-FITC (channel 2), Ly6G-PE (channel 3), mCherry (for mChLgyLRV1+ parasites) (channel 4), CD11b-PECy5 (channel 5), CD206-biotin revealed with SAV-PECy7 (channel 6), DAPI (channel 7), CD45.2-BV510 (channel 8), CCR2-APC (channel 11), and F4/80-APC/Cy7 (channel 12). Panel 2 experimental samples contained images and data for brightfield (channels 1 and 9), CD19-FITC (channel 2), NK 1.1-PE (channel 3), mCherry (for mChLgyLRV1+ parasites) (channel 4), CD3-PECy5 (channel 5), CD45-PECy7 (channel 6), DAPI (channel 7), MHC II-BV510 (channel 8), CD11c-APC (channel 11), and side scatter (SSC, channel 12). Single-color controls for both panels were acquired to generate the compensation matrixes, which were applied to each panel, respectively.

For immuno-fluorescence microscopy, images were partly acquired using an Olympus VS120-SL full slide scanner with a 20×/0.75 air objective (using Olympus OlyVIA software), at the EPFL BioImaging & Optics Platform (BIOP), or with Zeiss Axio-Imager Z1, Upright in Cellular Imaging Facility, Epalinges (using Axiovision SE64 rel 4.9.1 software). For the images acquired using the Olympus VS120-SL full slide scanner, individual images were acquired using the indicated fluorescent channels having the same exposure time employed across different samples. For *in vivo* bioluminescence imaging and quantification of parasite burden and inflammation, parasite bioluminescence produced in the mouse footpads and other visible secondary lesions (like tail and snout) were acquired with *In-Vivo* Xtreme II (Bruker).

### Software used for analysis

For cell suspension prepared for each LN and tissue, cell numbers and cell viability were assessed *via* Trypan blue (Sigma-Aldrich) exclusion using an improved counting Neubauer chamber (Assistant^®^, catalog number: 40442). For limiting dilution assay and analysis, parasite number was determined from the lowest cell concentration from which promastigotes could be grown using the ESTIMFRE software. The corresponding heatmaps were generated using the R ComplexHeatmap package. The color gradient represented in the heatmaps are correlated to the number of + signs assigned in terms of infection in progressive dilution series through LDA for each organ, where increased color intensity corresponded to higher parasite load.

For RT-qPCR, gene expression was analyzed using the threshold cycle (CT) method 2^−ΔΔCT^. Data analyzed for *Leishmania Kmp11* gene expression in different organs of the infected mice were assessed using predetermined levels of *Kmp11* expression in *Lgy* parasites and normalized to the total RNA quantity of the sample.

For bright light and fluorescent lymphangiography, acquired images were processed using LAS AF 6000 software along with processing, tiling, and stitching on Photoshop (version 21.2.0), in case of fluorescence-based images of whole mouse (represented in **Figure 5**).

For immuno-fluorescence microscopy, images were extracted using the VSI reader action bar [provided by the EPFL BioImaging & Optics Platform (BIOP)]. Olympus slide scanner software (OlyVIA v.2.6) was employed to directly analyze the images from different groups by adjusting the same contrast and brightness settings, across different groups.

For conventional FACS analysis, data were analyzed with FlowJo software (Tree Star, v10.0.6). For data analyses, a minimum of 50,000 events (cells) were evaluated at all times, by back-gating from CD45+/− stained cells. For imaging FACS, analysis was done using IDEAS (Image Data Exploration and Analysis Software) 6.2 software (Amnis Corporation).

For mice *in vivo* bioluminescence imaging and quantification of parasite burden and inflammation, acquired images were analyzed using Molecular Imaging (MI) software (Bruker), setting specific ROIs on FP, tail, etc., and expressing the measured bioluminescence signals in units of photons per second (p/s).

### Additional information on calculations and gating strategy

#### Cell population calculation through flow cytometry

A total of 1–2 million cells were stained and collected on each measurement for both kinds of cytometer.

For conventional FACS analysis, data were analyzed with FlowJo software (Tree Star, v10.0.6). For data analyses, a minimum of 50,000 events (cells) were evaluated at all times, by back-gating from CD45+/− stained cells. The absolute number of total leukocytes or each represented cell type was quantified by multiplying the total number of cells observed by hemocytometer counting with the percentage of those individual cells (on total CD45+ cells) determined by flow cytometry. The absolute number of each leukocyte subset (for example, 1A8, Ly6C, Ly6G, CD11b and, CCR2) was determined by multiplying the percentage of each gated population by the total number of CD45^+^ cells. For imaging FACS, analysis was done using IDEAS (Image Data Exploration and Analysis Software) 6.2 software (Amnis Corporation). Cell internalization versus non-internalization, free or sticking to cell surface or being in clumps for parasites, was defined using the internalization of the bright parasite spots within the membrane marker mask. Calculations and analyses of cell population were done exactly as defined for conventional FACS studies. For the distribution of mCherry^+^ parasites on cell-associated or non-cell-associated TER119^-^CD45+ immune cells, we calculated it from the point of view of the mCherry^+^ parasites, distributing the total numbers of mCherry^+^ parasites observed in any organ over its numbers that were either colocalized with TER119^−^CD45^+^ membrane marker (thus, cell associated) or not (non-cell-associated).

#### Gating strategy for Panels 1 and 2 of flow-cytometric analysis

For imaging flow cytometry (Imaging Stream^®^ analysis), samples were run in a two-camera, 12-channel Image-StreamX multispectral imaging flow cytometer (Amnis, Luminex Corporation) at low speed and highest magnification (60×). Instrument setup and performance tracking were performed daily using the Amnis^®^ SpeedBead^®^ Kit (Luminex Corporation) for verifying optimal instrument performance. Cells were excited using a 405-nm laser (25 mW), a 488-nm laser (100 mW), a 561-nm yellow laser (200 mW), and a 642-nm red laser (150 mW). Only events with a brightfield area >5 µm² (to exclude cell debris) and non-saturating pixels were collected (as described in Henery et al., 2008). Data were acquired for a minimum of 50,000 events/sample. Panel design was based on antigen expression density, fluorochrome brightness, and reagent availability in each panel for the four-laser, 12-parameter ImageStreamX. Preparation of cell suspension and staining were done exactly as described previously for regular flow-cytometric analysis and passed freshly in two panels (panels 1 and 2), as mentioned. Panel 1 experimental samples contained images and data for brightfield (channels 1 and 9), Ly6C-FITC (channel 2), Ly6G-PE (channel 3), mCherry (for mCh*Lgy*LRV1+ parasites) (channel 4), CD11b-PECy5 (channel 5), CD206-biotin revealed with SAV-PECy7 (channel 6), DAPI (channel 7), CD45.2-BV510 (channel 8), CCR2-APC (channel 11), and F4/80-APC/Cy7 (channel 12). Panel 2 experimental samples contained images and data for brightfield (channels 1 and 9), CD19-FITC (channel 2), NK 1.1-PE (channel 3), mCherry (for mCh*Lgy*LRV1+ parasites) (channel 4), CD3-PECy5 (channel 5), CD45-PECy7 (channel 6), DAPI (channel 7), MHC II-BV510 (channel 8), CD11c-APC (channel 11), and side scatter (SSC, channel 12). Single-color controls for both panels were acquired to generate the compensation matrixes, which were applied to each panel, respectively, prior to analysis using IDEAS (Image Data Exploration and Analysis Software) 6.2 software (Amnis Corporation). Similar gating strategy to encompass the infections in different cell types was applied in conventional FACS as well. Thus, a representative example of gating strategy for both kinds of cytometer measurement (conventional and Amnis) is provided as auxiliary **Supplementary 1**, **2** in separate files (as they are too heavy in size), to define the gating strategy for panels 1 and 2, respectively.

### Statistical analysis

GraphPad Prism8 [version 8.1.1(330)] was pertinently used to generate all the graphs and related statistical analysis. For single-point analysis on bar graphs, unpaired Student’s test was used, while repeated-measures two-way ANOVA test was used for x/y curves, with Bonferroni’s post-test correction. Significance was reached with p-values of 0.05, and p-values were represented in four ranks as * for p < 0.05, ** for p < 0.01, *** for p < 0.001, and **** for p < 0.0001. NS means non-significant statistical difference and was majorly not represented on graphs.

## Results

### *Lgy*LRV1+ induces a progressively severe metastatic phenotype in *Ifng^−/−^* mice

**Figure 1:**
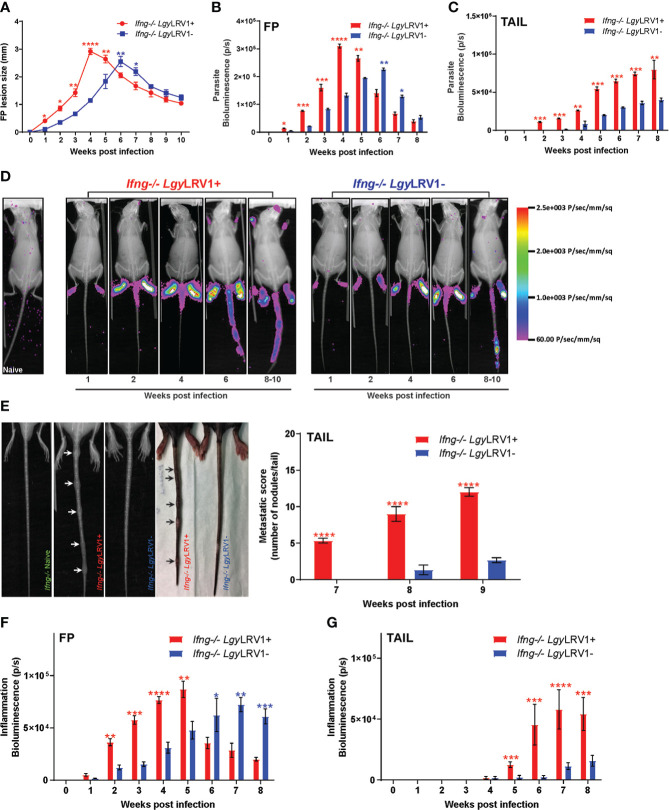
*Lgy*LRV1+ parasites induced a progressively severe metastatic phenotype in Ifng ^-/-^ mice.**(A–G)** Ifng^-/-^ mice were infected in the hind FP with 1x10^6^ *Lgy*LRV1+ or *Lgy*LRV1- stationary-phase metacyclic promastigotes. Different proxies of disease progression were documented weekly from W1-W8 p.i, between *Lgy*LRV1+ (red) and *Lgy*LRV1- (blue) groups by measuring **(A)** FP swelling; **(B–D)** parasite burden measured in terms of parasite bio-luminescence in **(B)** FP ; **(C)** Tail ; and **(D)** representative images comparing the pattern of parasite dissemination along whole mice body between both groups with time; **(E)** x-ray pictures, along with camera pictures, showing the appearance of metastatic nodules, quantified by absolute count of the number of nodules per tail in both groups. **(F, G)** inflammation measured in terms of myeloid peroxidase activity, quantified by parasite bio-luminescence weekly in **(F)**FP and **(G)** Tail. Represented statistical data are a pool of three independent experiments whereas associated images are representative from one of these experiments of the pool, with n ≥ 10 mice /group/ time point. Each experiment was repeated 5 times independently with similar results. The statistics are shown as mean ± SEM with p values determined for significance, calculated at each mentioned time-point between both groups, using two-way ANOVA with Bonferroni’s post-test for A-C and E-G. Stars of significance are determined as *p < 0.05; **p < 0.01; ***p < 0.001; ****p < 0.0001. Non-significant differences (ns) were not represented on the graphs while stars for significance were annotated in red if the value associated with LgyLRV1+ is > LgyLRV1−, and in blue if LgyLRV1− > LgyLRV1+, at each time- point, for easy reference.

**Figure 2:**
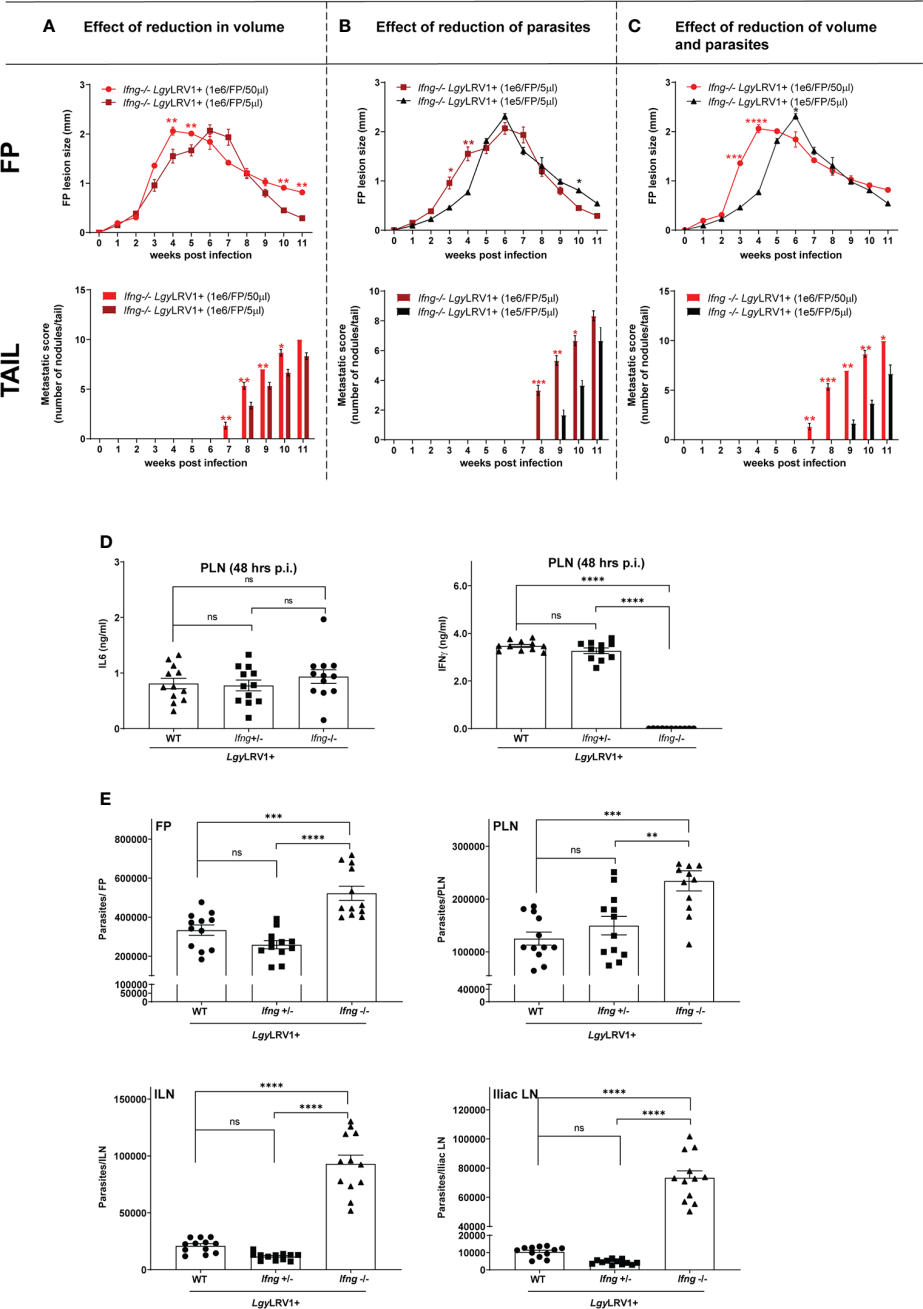
**(A–C)** Reduction in parasite number and volume of infection just delayed the metastatic phenotype in *Ifng^−/−^ Lgy*LRV1+ model: *Ifng^−/−^* mice were infected in the hind FPs with different numbers of *Lgy*LRV1+ stationary-phase metacyclic promastigotes, in different volumes as defined by group. Proxies of disease progression in terms of FP lesions (in mm) and metastatic outcome on TAIL were documented weekly, along the course of infection, between **(A)** group infected with 1×10^6^ parasites/FP in 50 μl versus group infected with 1×10^6^ parasites/FP in 5 μl, **(B)** group infected with 1×10^6^ parasites/FP in 5 μl versus group infected with 1×10^5^ parasites/FP in 5 μl, and **(C)** group infected with 1×10^6^ parasites/FP in 50 μl versus group infected with 1×10^5^ parasites/FP in 5 μl. **(D)** *Ifng* gene is haplo-sufficient for the production of active IFN-γ cytokine: WT, *Ifng^+/−^* and *Ifng^−/−^* mice groups were infected in the hind FP with 1×10^6^ *Lgy*LRV1+ stationary-phase metacyclic promastigotes. Cell suspension supernatants from PLN, extracted from each infected group, were measured for the secreted levels of IL-6 and IFN-γ cytokines, at 48 h p.i., by ELISA. **(E)** Both WT and *Ifng^+/-^* infected groups showed equivalent parasite burden in infected organs, at the peak of infection: WT, *Ifng^+/−^*, and *Ifng^−/−^* mice groups were infected in the hind FP with 1×10^6^ *Lgy*LRV1+ stationary-phase metacyclic promastigotes. Parasite burden assessed in terms of relative RNA expression levels of parasite specific *Kmp11* gene, at week 4 p.i. in the FPs, PLN, ILN, and iliac LN, in each infected group by RT-qPCR. Data are expressed (for each set of experiments above) as mean ± SEM from a pool of three independent experiments (n=12 mice/group) and repeated independently four times with similar results, p-values determined for significance, calculated at each mentioned time point between different groups, using two-way ANOVA with Bonferroni’s post-test; significance is indicated as *p < 0.05, **p < 0.01, ***p < 0.001, and ****p < 0.0001; ns, non-significant differences.

Interferon gamma (IFN-γ) is crucial for immunity against intracellular pathogens such as *Leishmania* parasites (Rossi et al., 2017), and in its absence, patients are prone to chronic metastatic complications (Hartley et al., 2016). As experimental model for the dissemination in response to an LRV1-dependent acute inflammatory stimulus, we infected groups of *Ifng^−/−^* mice in the hind FPs with either *Lgy*LRV1+ or *Lgy*LRV1−. *Ifng^−/−^* mice infected with *Lgy*LRV1+ showed a significant increase in FP lesion (**Figure 1A**) and parasite burden in FP (**Figure 1B**). Both peaked at week 4 post-infection (W4 p.i.), while the parasite burden increased progressively in the tail and remained significantly higher in the *Lgy*LRV1+ group (**Figure 1C** **)** from week 2 post-infection (W2 p.i.) till the end of infection, as compared to their *Lgy*LRV1−-infected counterparts. A detailed kinetic and comparison of parasite dissemination in *Ifng^−/−^* mice infected with either *Lgy*LRV1+ or *Lgy*LRV1− *in vivo* were documented weekly at each time point of infection with representative mice from both groups by superimposing X-ray pictures and bioluminescence of parasites expressing the LUC gene (**Figure 1D**). This confirmed that parasite dissemination in distant secondary organs, such as the tail (around W2 p.i.), or even in the forelimb and snout (around W8 p.i.), in the *Ifng^−/−^ Lgy*LRV1+ group appeared much earlier as compared to the *Ifng^−/−^ Lgy*LRV1− group, where the same phenotype appeared significantly delayed. Furthermore, the metastatic score, defined as the absolute count of the number of secondary nodules per tail, also appeared earlier and was significantly higher in the *Ifng^−/−^ Lgy*LRV1+ group from week 7 p.i. onwards, till the end of infection (**Figure 1E**). Moreover, inflammation measured by myeloperoxidase activity (Eren et al., 2016; Reverte et al., 2021) was significantly higher both in the FP (**Figure 1F**) and in the tail (**Figure 1G**) of the *Ifng^−/−^ Lgy*LRV1+ group consistently along the course of infection. We therefore confirmed and established the detailed kinetic of LRV1-associated disease exacerbation and increased metastatic outcome in *Ifng^−/−^ Lgy*LRV1+ mice model *in vivo*, as described earlier (Hartley et al., 2016). To further validate our model of parasite dissemination and metastasis, *Ifng^−/−^* mice were infected either with the same number of *Lgy*LRV1+ parasites in lesser volume of injection (i.e., in 5 µl, for volume control) (**Figure 2A**) or injected with 10-fold lesser number of *Lgy*LRV1+ (1×10^5^, for parasite number control) (**Figure 2B**). Results showed a delayed but similar final disease outcome in terms of lesion growth in footpads and metastatic score in the tail, as compared to an infection with 1×10^6^ parasites/50 µl/FP (**Figures 2A**–**C**). Additionally, the levels of IL6 and IFN-γ cytokine were measured on the cell-free supernatant isolated from the primary draining lymph node (PLN) at 48 h p.i. from *Ifng^−/−^*, *Ifng^+/−^*, and WT (*Ifng ^+/+^*) mice groups, infected with *Lgy*LRV1+. Interestingly, IL6 cytokine production was equal between all the three groups and IFN-γ production was found to be equal between both *Ifng^−/−^* and WT (*Ifng^+/+^*) group as compared to the negative control (*Ifng^−/−^* mice), thereby confirming the haplo-sufficiency of the IFN-γ allele *in vivo* (**Figure 2D**). Moreover, parasite burden was also found to be equal in the FP, PLN, ILN, and iliac LN, between the *Ifng^+/−^* and WT group and significantly lower in both these groups as compared to the *Ifng^−/−^* group, at the peak of infection (W4 p.i.) (**Figure 2E**). These results collectively established our model of IFN-γ-dependent infectious metastasis and *Leishmania* RNA virus (LRV1+)-associated disease exacerbation, detailing the various hallmarks of disease progression in terms of *Lgy* infection.

### LRV1 induces hyper-inflammation of the draining LNs in metastatic *Ifng^−/−^* mice

**Figure 3:**
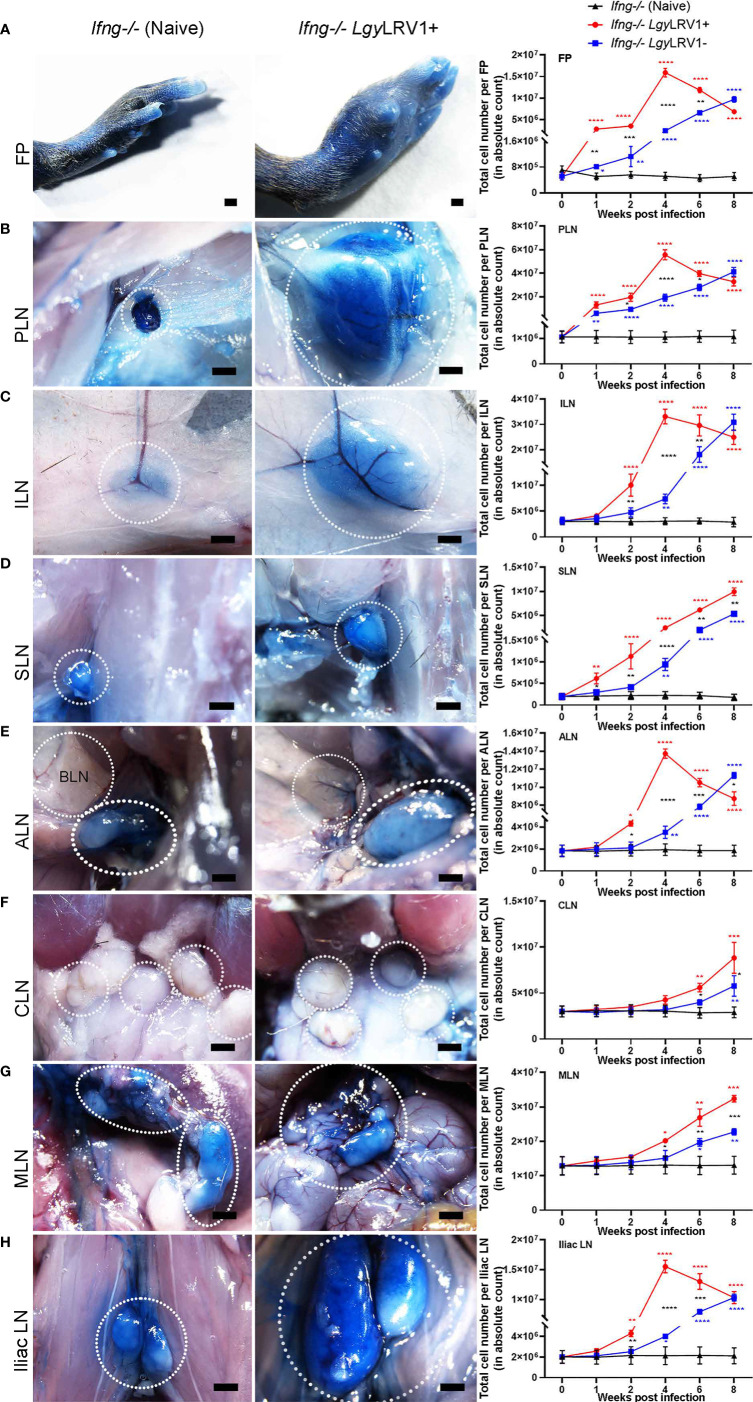
*Lgy*LRV1+ parasites triggered increased Evan’s blue drainage and hyper-inflammation of draining LNs. **(A–H)** Stereomicroscopic images (scale bars: 1 mm) of the LNs were taken, after injecting 5% Evan’s blue (in DPBS) for 20 min, in the hind FP of both *Ifng^−/−^* naive and *Ifng^−/−^ Lgy*LRV1+ at week 4 p.i. (as represented in columns 1 and 2, respectively). Simultaneously, *Ifng^−/−^* mice were infected at 1×10^6^/FP either with *Lgy*LRV1+ (*Ifng^−/−^ Lgy*LRV1+ in red) or *Lgy*LRV1− (*Ifng^−/−^ Lgy*LRV1− in blue), along with the uninfected PBS-injected control (*Ifng^−/−^* naive in black). The total number of cells in each of the three groups from W1 to W8 p.i. are represented in **(A)** FP, **(B)** PLN, **(C)** ILN, **(D)** SLN, **(E)** ALN, **(F)** CLN, **(G)** MLN, and **(H)** iliac LN in column 3. These cell counts of each organ are representative of three pooled experiment (n ≥ 18 mice/group/time point) and repeated at least three times independently with similar results for each group. Thus, represented statistical data are a pool of three independent experiments, whereas associated images are representative from one of these experiments of the pool, with n ≥ 5 mice/group/time point, and repeated five times with similar results. Statistics are represented as mean ± SD with p-values and significance determined by performing two-way ANOVA with Bonferroni’s post-test on cell counts of each group, at each time point. Only significant points were determined as *p < 0.05, **p < 0.01, ***p < 0.001, and ****p < 0.0001, and annotated in red (*Lgy*LRV1+) and in blue (*Lgy*LRV1−), as compared to naive. Significance between *Lgy*LRV1+ and *Lgy*LRV1−, at each time point, is denoted by black asterisk.

LNs draining their immediate organ such as popliteal LNs (PLNs) draining FP are used to characterize the immune response to an acute inflammatory stimulus (Morton et al., 2003; Saharinen and Petrova, 2004; Angeli et al., 2006). We therefore mapped the location of different lymph nodes (LNs) in naive *Ifng^−/−^* mice by injecting 5% Evan’s blue dye solution (**Supplementary Figures S1A–C** **)** for further extrapolating it to investigate the *in vivo* hubs of escaping infected cells in our infected metastatic model of FP infection (Van den Broeck et al., 2006; Harrell et al., 2007; Harrell et al., 2008). Thus, FP Evan’s blue lymphatic mapping of naive *Ifng^−/−^* mice and *Ifng^−/−^* mice infected in both FP 4 weeks previously, i.e., at week 4 (W4) post-infection (p.i.) with *Lgy*LRV1+, revealed a visible blue drainage and simultaneous LRV1 exacerbated cellular increase in FP, tail, and different LNs like popliteal (PLN), inguinal (ILN), sciatic (SLN), iliac LN, axillary (ALN), and mesenteric (MLN) as compared to their *Lgy*LRV1− counterparts (**Figures 3A**–**H**). Representative stereomicroscopic images showed an evident deep blue labeling and visible increase in size of popliteal (PLN), inguinal (ILN), sciatic (SLN), iliac LN, and axillary (ALN); faint blue labeling of mesenteric (MLN) while no labeling of brachial (BLN) and cervical (CLN) in both the naive and infected groups of *Ifng^−/−^* mice. Therefore, the visible hyper-inflammation of different blue-labeled draining LNs suggested a highly active LN contribution to *Lgy* parasite metastasis *in vivo.*

### LRV1 induces exacerbated parasite dissemination to lymph nodes

**Figure 4:**
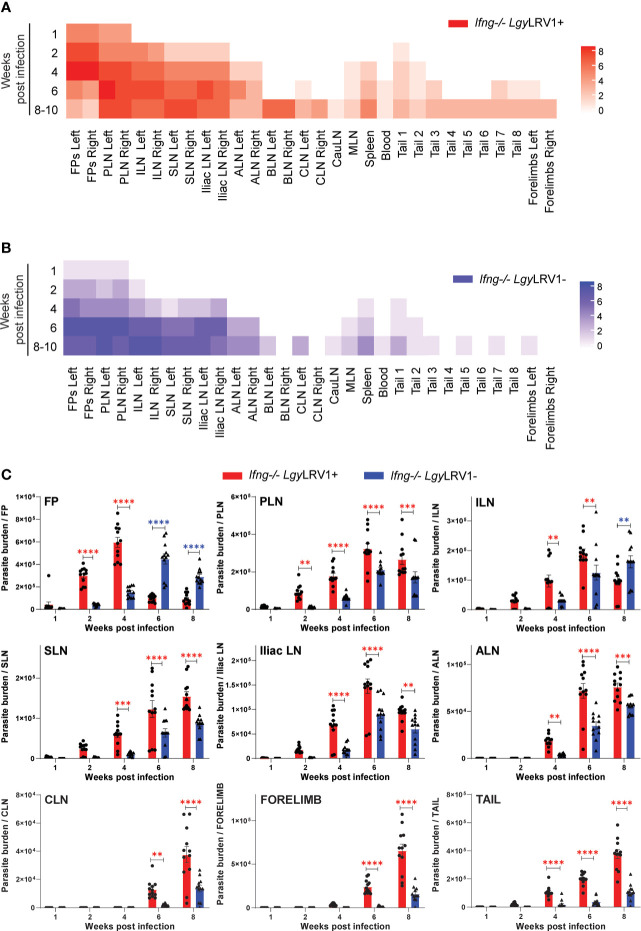
LRV1 causes exacerbated parasite dissemination through LNs into the tail. **(A, B)** Aforementioned organ suspension collected, shown as left and right, were subjected to limiting dilution analysis (LDA) from *Ifng^−/−^* mice infected in both hind FP with 1×10^6^ *Lgy*LRV1+ or *Lgy*LRV1− stationary-phase metacyclic promastigotes at weeks 1, 2, 4, 6, and 8–10 p.i. Corresponding heatmaps were generated using the R ComplexHeatmap package for **(A)** *Ifng^−/−^Lgy*LRV1+ group in red and **(B)** *Ifng^−/−^Lgy*LRV1− group in blue. The color gradient represented in the heatmaps are correlated to the number of + signs assigned in terms of infection in progressive dilution series through LDA for each organ, where increased color intensity corresponded to higher parasite load. **(C)** Parasite burden per organ was quantified through relative RNA expression levels of parasite-specific gene *Kmp11* by RT-qPCR, in FP, PLN, ILN, SLN, iliac LN, ALN, CLN, forelimb, and tail, between *Ifng^−/−^ Lgy*LRV1+ (red) and *Ifng^−/−^ Lgy*LRV1− (blue). Data are representative of three independent experiment pooled, with n ≥ 4 mice/group/time point, in each experiment. All statistics represent mean ± SEM, with p-values determined by two-way ANOVA. Significance is indicated as **p < 0.01, ***p < 0.001, and ****p < 0.0001, where they are annotated in red if the value associated with *Lgy*LRV1+ is > *Lgy*LRV1− and in blue if *Lgy*LRV1− > *Lgy*LRV1+, at each time point, for easy reference. Represented statistical data are a pool of three independent experiments, and the associated heatmap also represents the results of the pool of LDA in individual sets, with n ≥ 4 mice/group/time point, repeated at least five times independently.

We therefore collected the different LNs coming from the right and left sides of *Ifng^−/−^* mice groups infected with either *Lgy*LRV1+ or *Lgy*LRV1−, along with the FP, spleen, liver, kidney, and tail (divided into eight pieces: T1–T8), and subjected their organ suspensions to an LDA. This ensured the comparison of both *Lgy*LRV1+ or *Lgy*LRV1−-infected mice groups from week 1 to 10 p.i. LDA permits the recovery and culture of free-living motile promastigote parasites, serving as a proxy to quantify the degree of infection in each tissue. The higher inflammation correlated with the progressive presence and increase in *Lgy* parasites in the culture for different organs along the course of infection. As represented by heatmaps where increased color intensity corresponds to higher parasite load, parasites were detected in the culture in a defined order, first in PLN followed by ILN, SLN, and iliac LN showing parasite presence almost at the same time but not at equivalent level (**Figures 4A**, **B**). The more distant LNs, namely, brachial and superficial cervical (BLN and CLN, respectively), spleen, and blood showed increased parasite burden only towards the late phase of chronic infection (from W4 to W6 onwards). Major infected sites, namely, FP, PLN, ILN, SLN, iliac LN, ALN, forelimb, and tail, showed significantly higher quantifiable parasite burden in the *Lgy*LRV1+ group, as compared to their *Lgy*LRV1− counterparts (**Figure 4C**). A schematic elucidates the time-dependent comparison of *Lgy* dissemination through LNs, between the *Ifng^−/−^ Lgy*LRV1+ (red) and *Ifng^−/−^ Lgy*LRV1− (blue) infection groups, for visual reference (**Supplementary Figures S2A**–**E**). We further investigated if parasite dissemination occurred similarly in infections with other *Leishmania* species. Although we observed a similar dissemination pattern through LNs when *Ifng^−/−^* mice were infected with *L. major* (*Lmj*) (**Supplementary Figure S3A** **)**, at no point were secondary debilitating lesions observed, in any distant organ like the tail, snout, or forelimb (**Supplementary Figures S3B**–**D**). This strongly suggested that the observed disseminating phenotype was not *Leishmania* species specific but that the occurrence of debilitating lesions was.

### *Lgy*LRV1+ parasites disseminate through the lymphatics

**Figure 5:**
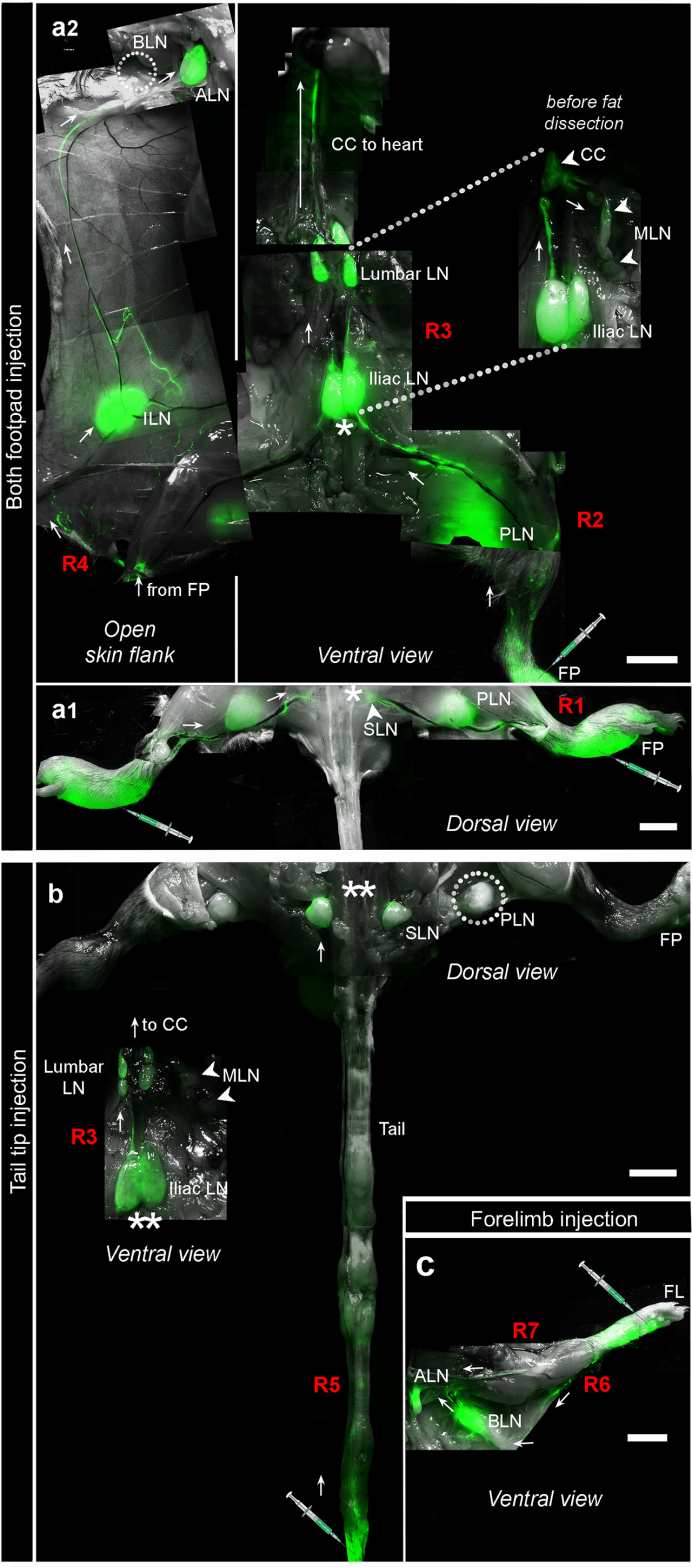
Fluorescent dextran angiography revealed lymphatic pathways of parasite dissemination. **(A–C)** *Ifng^−/−^* mice were infected in both FPs with 1×10^6^ *Lgy*LRV1+, and lymphatic connections were mapped through stereomicroscopy, by injecting 5 μl of fluorescein isothiocyanate (FITC) labeled dextran (2,000 kDa) at defined sites at different time points of infection, as follows: (6) in both FP in an *Ifng^−/−^ Lgy*LRV1+ (at week 4 p.i): a1, dorsal view of different lymphatic connections showing route R1 connecting FP to PLN to SLN to iliac ventrally, while BLN stays undrained; a2, ventral view of the lymphatic connections: revealing different routes emanating from the FP such as R2 connecting FP to iliac LN, R3 connecting iliac LN to CC to MLN to thoracic duct, and R4 connecting FP to ILN to ALN to subclavian vein. **(B)** At the tip of the tail of a metastatic *Ifng^−/−^ Lgy*LRV1+ (at week 8 p.i) revealing route R5, which drains the tail tip upwards to the SLN to iliac LN ventrally, followed by route R3 (described above). **(C)** In both forelimbs of an *Ifng^−/−^ Lgy*LRV1+ (at week 4 p.i) representing the lymphatic route R6, which connects the forelimb to BLN to ALN draining into the subclavian vein, and R7 connecting the forelimb drainage to ALN to the subclavian vein. Representation of different lymphatic drainage pattern observed in each group was derived by tiling several individual stereomicroscopic pictures (scale bar = 5mm). Each tilling represents one mouse from one experiment (to ensure common ends for stitching), with n ≥ 5 mice/group/experiment, and each kind of experiment was repeated three times with similar results.

Evan’s blue lymphangiography confirmed the active involvement of different draining LNs due to *Lgy* infection, thereby raising questions whether lymphatic connections emanating from the site of infection could act as the major routes for the dissemination of *Lgy* parasites resulting in severely inflamed LNs and, therefore, widespread metastasis. We thus explored the major lymphatic collecting vessels through fluorescent lymphangiography in infected *Ifng^−/−^* mice (Zheng et al., 2014; Wang et al., 2015; Yamaji et al., 2018) to test this hypothesis. At defined points of infection, *Ifng^−/−^* mice infected with *Lgy*LRV1+ were injected with 5 µl of fluorescein-isothiocyanate (FITC)-labeled dextran at three different sites to visually track the existing lymphatic connections. Injection in both hind FP at week 4 post-infection (W4 p.i.) showed four major collecting vessels that emanated from the FP: route 1 (R1) with one long lymphatic connection from FP to PLN to SLN draining into the iliac LN ventrally (**Figure 5A1**); route 2 (R2) connecting FP directly to iliac LN; route 3 (R3) observed from iliac LN to centrally located cisterna chyli (CC), while MLN is simultaneously positive, and all these connections ultimately drained upwards into the thoracic duct; and route 4 (R4) connecting the FP to ILN to ALN to the subclavian vein (**Figure 5A2**). Injection at the tip of the tail at W7–W8 p.i. revealed route 5 (R5) showing the collecting vessels from the tail tip upwards to the SLN to iliac LN ventrally, which ultimately joined route 3 (R3) upwards to the thoracic duct (**Figure 5B**). Finally, forelimb injection documented two major lymphatic connections: route 6 (R6) connecting the forelimb to BLN to ALN to the subclavian vein and route 7 (R7) connecting the forelimb directly to ALN to the subclavian vein region (**Figure 5C**). The different routes of lymphatic connections along with the direction of lymph flow are detailed comprehensively in schematic diagrams for each site of reference (**Supplementary Figures S4A**–**C**). These results collectively revealed the intricate lymphatic pathways of FP drainage, which were used by *Lgy* parasites in our metastatic model as indicated by the sequence of LNs infected over time (**Figure S4D**). Thus, we could conclude that the lymphatic vessels could participate in the dissemination of *Lgy* infection from the primary site, which then leads to the formation of loco-regional metastases in various organs, mainly in the LNs, and lead to their large-scale distribution.

### *Lgy*LRV1+ exhibits early infection of innate immune cells

**Figure 6:**
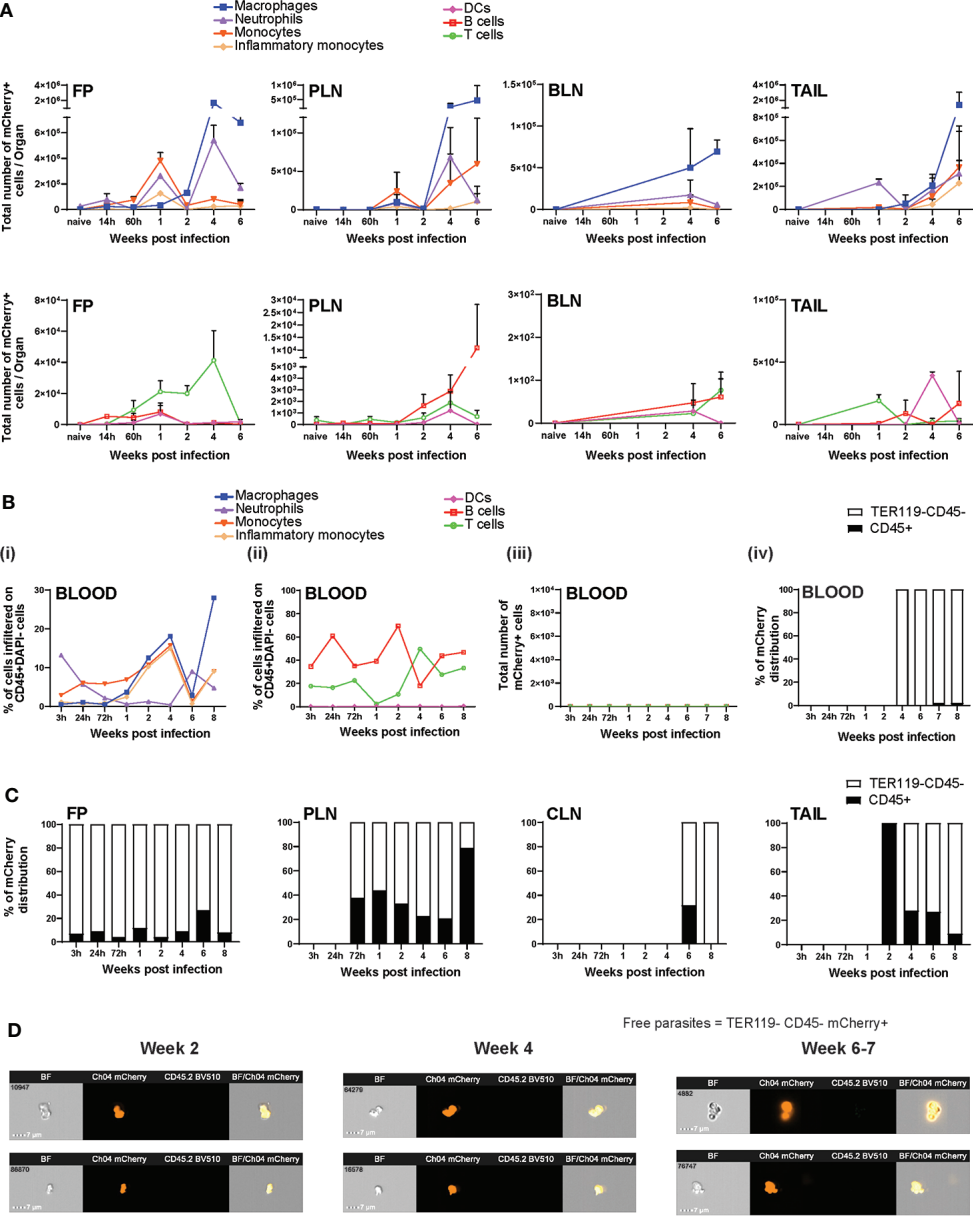
*Lgy*LRV1+ parasites existed as extracellular amastigotes in infected organs and blood. **(A–C)** *Ifng^−/−^* mice were infected in both FPs with 1×10^6^ mCherry-expressing mch*Lgy*LRV1+ promastigotes. Flow cytometric analysis revealed **(A)** the total number of mCherry^+^ cells of each cell type (defined below) in FP, PLN, BLN, and tail at W1, W2, W4, and W6 p.i. **(Bi, ii)** Percentage of infiltration of each cell type in the blood at 3 h, 24 h, 72 h, W1, W2, W4, W6, and W8 p.i. showing **(i)** neutrophils, monocytes, macrophage, and inflammatory monocytes; **(ii)** T cells, B cells, and DCs; **(iii)** total number of mCherry^+^ cells of each cell type; (iv) distribution of total mCherry observed in the blood on its CD45^+^ or TER119^−^CD45^−^ cells. **(C)** Distribution of total mCherry observed in FP, PLN, CLN, and tail over their respective CD45^+^ or TER119^−^CD45^−^ cells. **(D)** Representative images taken by imaging flow cytometry show mch*Lgy*LRV1+ amastigotes in the blood of *Ifng^−/−^ Lgy*LRV1+-infected mice, at W2, W4, and W6–W7 p.i. Corresponding samples from naive *Ifng^−/−^* mice (injected with PBS) were passed to set the baseline of increase for panel **(A)** (as negative control), while the data represented in panels **(B, C)** are already normalized to their naive values. Represented cell populations were defined by specific cell surface markers as follows: neutrophils (CD45^+^CD11b^+^Ly6G^+^), macrophages (CD45^+^CD11b^+^F4/80^+^), monocytes (CD45^+^CD11b^+^Ly6C^+^), inflammatory monocytes (CD45^+^CD11b^+^Ly6C^+^CCR2^+^), T cells (CD45^+^CD3^+^), B cells (CD45^+^CD19^+^), DCs (CD45^+^CD11b^+^CD11c^+^MHCII^+^), and free parasites (TER119^−^CD45^−^mCherry^+^). Represented statistical data are a pool of three independent experiments, whereas associated images are representative of one out of three or more independent experiments, with n ≥ 4 mice/group/time point. The statistics are given as mean ± SEM.

Once the various routes of dissemination were established, *Ifng^−/−^* mice were infected with fluorescent mCherry-expressing *Lgy*LRV1+ parasites (mCh*Lgy*LRV1+), and FP, PLN, BLN, and tail samples were collected at 3 h, 14 h, 60 h, W1, W2, W4, and W6 p.i. Flow-cytometric analysis of each enzymatically dissociated organ determined the total number of infected (mCherry^+^) cells for different immune cell populations (**Figure 6A**). Recruited neutrophils were infected first, followed by monocytes, inflammatory monocytes (expressing CCR2^+^), and then macrophages from W1 p.i. All these cells had immigrated from the blood and got infected in cycles along the course of infection, both in FP and PLN. Although BLN showed macrophage infection only from W4 p.i., all different kinds of innate cells were infected in the tail towards the second half of infection (**Figure 6A**). Imaging flow cytometry on samples from *Ifng^−/−^* mice infected with mCh*Lgy*LRV1+ showed that neutrophils were the first and most abundantly increased cells in systemic circulation and varied in abundance in cycles along the course of infection, followed by monocytes, inflammatory monocytes (expressing CCR2^+^), macrophages, and even B and T cells (**Figures 6Bi, ii**). However, none of these blood immune cells harbored significant parasite levels during the chronic phase of infection (**Figure 6B, ii**).

### Extracellular mCh*Lgy*LRV1+ amastigotes stick around recruited immune cells

Remarkably, 98%–99% of mCherry^+^ *Lgy*LRV1+ parasites in the blood were observed as free amastigotes towards the latter half of infection, i.e., as non-host-cell-associated mCherry^+^ amastigotes in the TER119^-^CD45^−^ population (**Figure 6B, iv**). However, approximately 1%–2% of the total parasites were also CD45^+^ immune cell associated, infecting neutrophils and monocytes in lower numbers (**Figure 6B, iv7**). We thus investigated whether the total mCherry fluorescence observed in different organs was due to free amastigotes (i.e., in the TER119^−^CD45^−^ non-hematopoietic cell population) or due to infected cells (associated to TER119^−^CD45^+^ immune cells). Our results showed that the majority of the mCherry^+^ parasite fluorescence (≥90% at each time point of infection, calculated from the parasite perspective) observed in each of the FP, PLN, CLN, and tail was due to non-immune cell-associated free amastigotes as compared to that associated with CD45^+^ cells (**Figure 6C**). Imaging flow cytometry also confirmed the presence of free mCherry^+^ parasites in the blood at W2, W4, and W6–W7 p.i. (**Figure 6D**).

**Figure 7:**
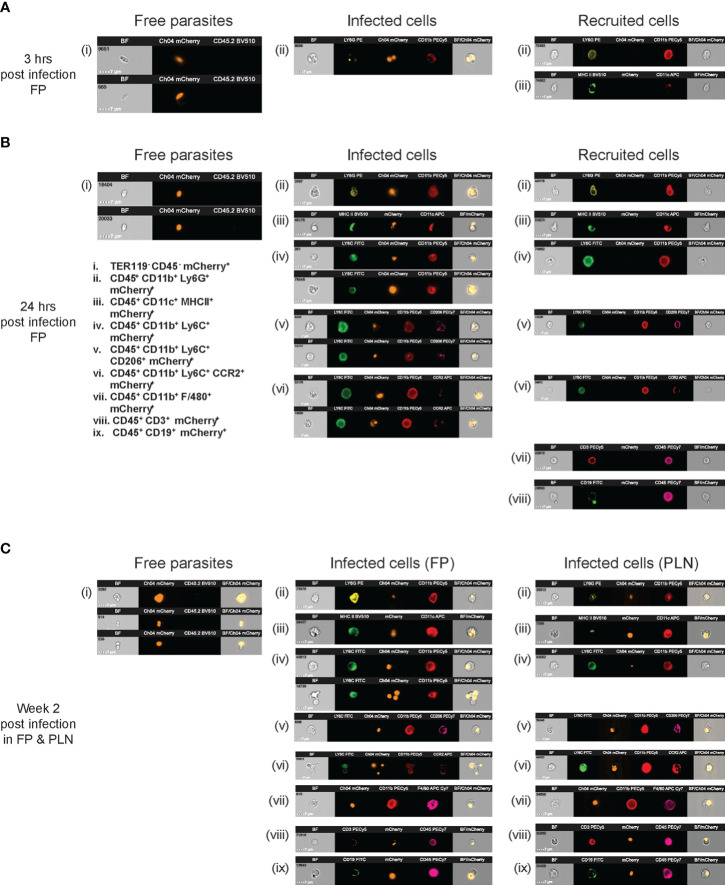
Within infected organs, *Lgy*LRV1+ mostly appeared as extracellular parasites, bound to the surface of immune cells and also as free-floating amastigotes. **(A–C)** *Ifng^−/−^* mice were infected in the hind both FP with 1×10^6^ mCherry-expressing metacyclic mCh*Lgy*LRV1+ promastigotes. Sets of five mice were euthanized at 3 h, 24 h, and W2 p.i., to collect FP and PLN, which were then homogenized and digested, and different kinds of cell contents were analyzed by imaging flow cytometry (as detailed in the *Materials and methods*). Different states of association of mCh*Lgy*LRV1+ parasites with respect to the infiltrated immune cells were defined as “infected” (parasites internalized by CD45^+^ immune cell or stuck to their surface), “recruited” (non-infected, infiltrated CD45^+^ immune cells), and as “free” (extracellular mCherry^+^ parasites, not bound or internalized by any CD45^+^ immune cells). Representative images (scale, 7 μm) of **(A, B)** recruited/infected individual immune cells and free parasites are thus illustrated in **(A)** FP at 3 h p.i. **(B)** FP at 24 h p.i., and only free parasites and infected cells in **(C)** FP and PLN at W2 p.i. Individual immune cell populations were defined by specific marker sets as follows: neutrophils (CD45^+^CD11b^+^Ly6G^+^), DCs (CD45^+^CD11b^+^CD11c^+^MHCII^+^), classical monocytes (CD45^+^CD11b^+^Ly6C^+^), resident mannose receptor monocytes (CD45+CD11b+CD206^+^Ly6C+), inflammatory monocytes of CCR2 origin (CD45^+^CD11b^+^Ly6c^+^CCR2^+^), macrophages (CD45^+^CD11b^+^F4/80^+^), T cells (CD45^+^CD3^+^), B cells (CD45^+^CD19^+^), free parasites (DAPI^−^TER119^−^CD45^-^mCherry^+^), while each of their infected counterparts were associated with an additional mCherry^+^ signal, as defined in the figure corroboratively. Free parasites were defined as TER119^−^CD45^−^mCherry^+^ non-immune cell-associated, extracellular amastigotes. Images are representative of the pool of three independent imaging experiments, with n ≥ 5 mice/group/time point.

Imaging flow cytometry was thus further employed to understand the unexpected presence of a significantly lower number of infected cells but higher percentage of free parasites in each infected organ. Representative images revealed a significantly higher recruitment of neutrophils along with few DCs, as the first cells to arrive at the site of infection (FP) approximately 3 h p.i. While recruited neutrophils took up parasites and thus exhibited significant mCherry internalization, DCs appeared practically uninfected, and the injected mCh*LgyLRV1+* parasites mostly appeared as flagellated extracellular promastigotes in the FP of infected *Ifng^−/−^* mice at 3 h p.i (**Figure 7A**). Although various other immune cell types showed increase in numbers within 24 h p.i. at the site of infection, only neutrophils and various subsets of monocytes (defined from ii to ix in **Figure 7B**) appeared to have internalized only a part of the parasite on site by this time. The mCh*LgyLRV1+* parasites on site appeared sequestered and more roundish, mostly existing as non-cell-associated extracellular amastigotes around the recruited immune cells (**Figure 7B**). Furthermore, both FP and PLN showed the maximum number of infected cells (as described previously in FACS studies, too) at W2 p.i. We indeed observed a significant amount of mCh*LgyLRV1+* parasites, either internalized by several subsets of monocytes, neutrophils, and macrophages, while a part of them stayed non-internalized around various recruited adaptive and innate cells, as extracellular amastigotes both in FP and PLN, at even W2 p.i. Collectively, we observed that mCh*LgyLRV1+* parasites were mostly distributed in three independent categories: (a) within neutrophils and, to a lesser extent, in monocytes and its various subtypes and in macrophages; (b) extracellularly around various innate and adaptive cell types; (c) majority as free/extracellular parasites or as clumps of parasites in all infected organs, as represented at 3 and 24 h p.i. in FP (**Figures 7A**, **B**) and at W2 p.i. in both FP and PLN (**Figure 7C**). Thus, the presence of these non-cell-associated amastigotes in various infected organs and even blood (during chronic phase of infection) pointed towards the possibility of non-cellular transport of *Lgy* parasites as well.

### Extracellular mCh*Lgy*LRV1+ amastigotes disseminate *via* lymphatics to blood

**Figure 8:**
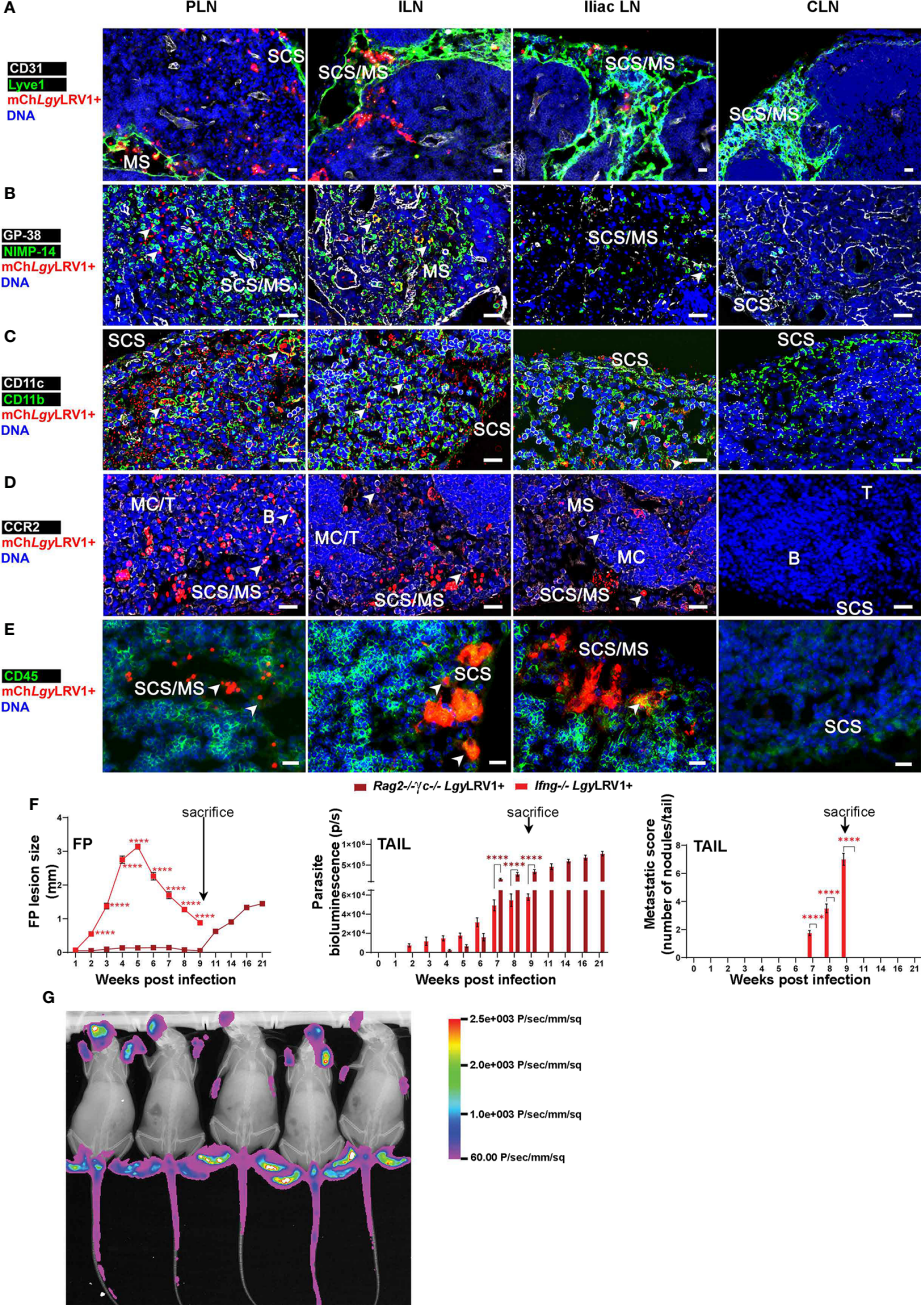
*Lgy*LRV1+ parasites dissemination was not dependent on a single immune cell type. **(A–E)** *Ifng^−/−^* mice were infected in both FPs with 1×10^6^ mch*Lgy*LRV1+ promastigotes and euthanized at W2 p.i. to recover PLN, ILN, iliac LN, and CLN for OCT embedding. Cryostat sections of 8–10μm of each of these LNs were subjected to anti-mCherry and DAPI (DNA) staining along with: **(A)** CD31 and Lyve-1, staining the blood vessels and lymphatic vessels, respectively; **(B)** GP-38 and NIMP-14 for staining fibroblastic reticular cells (FRCs) and neutrophils, respectively; **(C)** CD11c and CD11b for staining DCs and macrophages, respectively; **(D)** CCR2 staining inflammatory monocytes; and **(E)** CD45, as a pan marker for staining the myeloid immune cells. Data are representative of two to three experiments with at least two mice and six LNs/mouse. SCS, sub-capsular sinus; B/T, B-/T-cell zone in LNs; MC/MS/IL, medullary cord/medullary sinus/intranodal lymphatics, respectively (scale bars, 20 μm). **(F, G)** Groups of *Rag2^−/−^γc^−/−^* and *Ifng^−/−^* mice were infected in both FPs with 1×10^6^ *Lgy*LRV1+ promastigotes: **(F)** several measures of disease progression like FP swelling, parasite bioluminescence in tail, and metastatic score in tail were documented in each group along W1–W21 p.i. **(G)** Representative X-ray image nested with bioluminescent *Lgy*LRV1+ migration in *Rag2^−/−^γc^−/−^* mice at W21 p.i. Represented statistical data **(F,G)** are a pool of four independent experiments, whereas associated images **(A–E)** are representative from one of these experiments of the pool, repeated four times with similar results; n ≥ 6 mice/group/time point. Statistics are given as mean ± SEM, with p-values determined by two-way ANOVA. Significance is indicated as ****p < 0.0001.

Following our FACS analysis, we further assessed parasite localization in the different LNs *via* histological analysis. Immunofluorescence (IF) microscopy was performed on PLN (as the first LN shown to drain the FP), on ILN, and on iliac LN (as the two next draining LNs of FP-derived lymph), and in parallel on CLN as the non-draining negative control LN from *Ifng^−/−^* mice infected with mCh*Lgy*LRV1+ at W2 p.i. As expected, we detected mCherry^+^ *Lgy* parasites both in the FP and PLN from infected *Ifng^−/−^* mice (W2 p.i) but not in uninfected FP and PLN from *Ifng^−/−^* mice (**Supplementary Figure S5A**). Additionally, collagen IV and B220 staining of the basement membrane and B-cell follicles, respectively, elucidated the different zones in each of these massively swollen, infected LNs and localized mCherry^+^ parasites mainly within the subcapsular sinus (SCS) and medullary sinus (MS) spaces (**Supplementary Figure S5B**). We observed that mCh*Lgy*LRV1+ parasites decreased progressively in number from PLN to ILN and furthermore in iliac LN, as based on their distance to the FP, while being practically absent in the most distant CLN (**Figures 8A**–**E**). Subsequently, we stained these LNs for different vascular cell types. We confirmed that the *Lgy* parasites were mostly located in Lyve1^+^ lymphatic sinus space, both in the SCS and MS of each infected LN, while the CD31^+^ blood vessels showed a meager-to-no mCherry^+^ co-localization at this point of infection (**Figure 8A**).

### Infected organs are populated with extracellular mCh*Lgy*LRV1+ amastigotes

**Figure 9:**
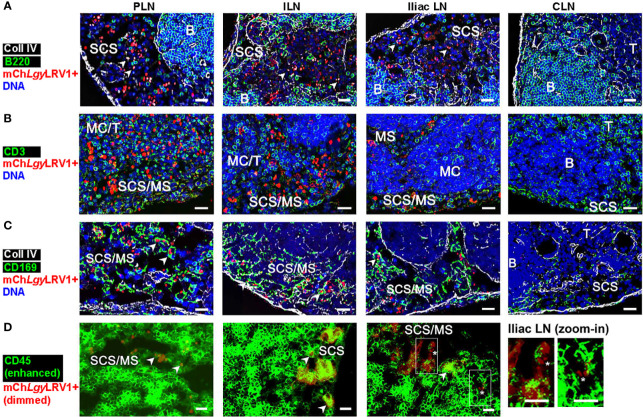
*Lgy*LRV1+ existed as extracellular amastigotes, showing little co-localization with most immune cells on site. **(A–C)** *Ifng^−/−^* mice were infected in hind FPs with 1×10^6^ mch*Lgy*LRV1+ promastigotes and euthanized at W2 p.i. to recover different LNs for OCT embedding. Cryostat sections of 8–10 μm from infected PLN, ILN, iliac LN, and CLN (as negative control) were stained with DAPI (for DNA) and anti-mCherry (for mCh*Lgy*LRV1+ parasites) labels and stained simultaneously for **(A)** B220 and collagen IV (Coll IV) antibodies for staining B cells and the basement membrane of vessels and its conduits, respectively; **(B)** CD3^+^ antibody for staining myeloid T cells; **(C)** Coll IV and CD169 antibodies for staining basement membrane of vessels and LN resident macrophages, respectively. Representative images were obtained from the slide scanner (scale bar, 20 μm). **(D)** Cryostat sections of 8–10 μm from infected LNs—PLN, ILN, and iliac LN—were subjected to CD45 and anti-mCherry antibodies for histological staining of all myeloid immune cells and mch*Lgy*LRV1+ parasites, respectively. CD45 signal was pushed to saturation (enhanced) while simultaneously dimming the mCherry signal to check if free mCherry^+^ extracellular amastigotes were still observed. These free amastigotes are pointed by white asterisk and also represented in a zoom-in section of iliac LN additionally. Representative images were obtained using Zeiss Axiovision (scale bar, 20 μm). White arrowheads represent co-localized mCherry+ *Lgy*LRV1+ with respectively mentioned cell markers. All data are representative of a pool of two to three experiments with n ≥ 2 mice/group and six LNs/mouse. SCS, sub-capsular sinus; B/T, B-/T-cell zone in LNs; MC/MS/IL, medullary chord/medullary sinus/intranodal lymphatics, respectively.

As neutrophils and fibroblastic reticular cell (FRCs) are known to be infected by *Leishmania* parasites (Bogdan et al., 2000; Hurrell et al., 2016), the FRC network and neutrophil infiltration were investigated by GP-38 and NIMP-14 staining, respectively. While FRCs showed little change in their architecture, we observed massive neutrophil recruitment that appeared to correlate with the mCherry^+^ parasite presence in the primary drained PLN and even in the more distantly drained ILN and iliac LN, while very few neutrophils were observed in the CLN. However, most recruited neutrophils and resident FRCs were not found to be harboring mCherry+ parasites (**Figure 8B**). Additionally, these LNs were also stained with antibodies for other innate immune cell types. While CD11b^+^ monocytes/macrophages and CD169^+^ macrophages frequently showed intracellular parasite staining (**Figures 8C**, **9C**, respectively), significantly lower numbers of CD11c^+^ DCs harbored parasites (**Figure 8C**). CCR2^+^ inflammatory monocytes were less frequent in the infected LNs and showed very low levels of infection/co-localization with the mCh*Lgy*LRV1+ parasites present within the organs, as observed earlier for neutrophils as well (**Figure 8D**). Moreover, both B and T cells showed no significant parasite internalization but partially exhibited cell contact with mCherry^+^ amastigotes, possibly attached to their cell surface (**Figures 9A**, **B**). Also by histology, the parasites found inside LNs appeared mostly as extracellular amastigotes. To further test this finding, a pan CD45^+^ staining for all immune cells was performed on each of the mentioned LNs, revealing that mCh*Lgy*LRV1+ parasites existed mainly in three forms: (a) partly internalized within the recruited immune cells; (b) partly around the immune cells, possibly in their close vicinity but not internalized; and (c) majority as non-cell associated (CD45^−^), presumably unbound extracellular amastigotes or its clumps (**Figure 8E**). Furthermore, when this CD45^+^ signal was further pushed to saturation, although it exhibited more CD45^+^-mCherry^+^ parasite co-localization in these staining, the overall phenotype of mCherry^+^ parasites appearing mostly as non-immune cell-bound, extracellular amastigotes still persisted (**Figure 9D**).

### *Lgy*LRV1+ dissemination is not solely immune cell dependent

**Figure 10:**
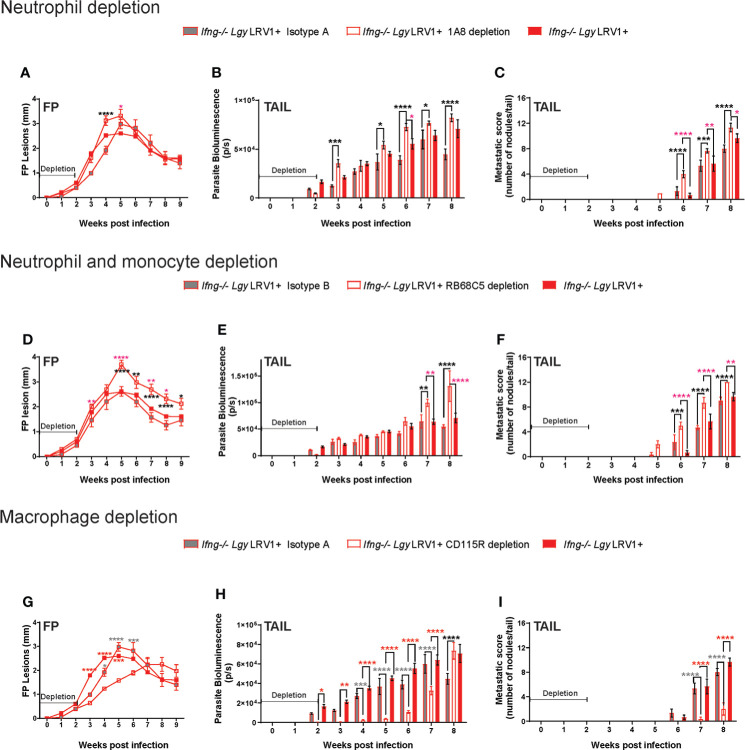
*Lgy*LRV1+ dissemination was not dependent on any particular innate cell type *in vivo*. **(A–C)** Individual groups of six *Ifng^−/−^* mice were depleted prior to infection for: **(A)** neutrophils, using anti- Ly6G monoclonal antibody (mAb), **(B)** monocytes and neutrophils together, using RB6-8C5 mAb, and **(c)** macrophages, using anti-CD11R mAb, treating additional *Ifng^−/−^* mice groups with either isotype A or B mAb as their respective treatment controls (suggested as per manufacturer’s protocol). *Ifng^−/−^* mice (injected with only DPBS) were simultaneously maintained as undepleted control for comparing all depletions. Depletion regime for each mAb is detailed in *Materials and methods*. Each group was then infected in both FPs with 1×10^6^ *Lgy*LRV1+ promastigotes. Individual depletion in each group was continued till 2 weeks p.i. The following measures of disease progression were documented weekly from W1 to W8 p.i.: **(A,D,G)** FP swelling, i.e., proxy of parasite growth; **(B,E,H)** parasite bioluminescence in the tail, i.e., measure of parasite dissemination, and **(C,F,I)** metastatic score, i.e., measure of systemic debilitating metastases, as a result of **(A–C)** neutrophils/PMN depletion, **(D–F)** RB6-8C5/simultaneous monocyte and neutrophil depletion, and **(G–I)** CD115R/macrophage depletion. All statistical data are representative of a pool of four independent experiments, whereas associated images are representative from one of these experiments of the pool with n ≥ 6 mice/group/time point. Statistics, p values and significance are exactly as described for **Figure 8**. Asterisks of significance are in black when the value associated with the depletion group > the isotype treated (isotype A/B) group, gray when isotype A/B > depletions, red when *Lgy*LRV1+(only) > depletions, and magenta when depletions is > *Lgy*LRV1+(only) for easy visual reference.

To further assess the role of the various innate immune cells as vehicles of parasite dissemination, we performed *in vivo* depletions of the major cell types that were recruited in large numbers and also partially infected at the primary site of infection (FP) and in the first draining LN (PLN) during the early phase of infection (as previously observed through FACS studies, too). We observed that neutrophils and monocytes had limited roles in terms of parasite control, as assessed by parasite numbers in the FP and tail metastasis, while macrophage depletion led to a significant reduction in parasite numbers in the early but not late infection phase (**Figures 10A**, **C**, **D**, **F**, **G**, **I**). However, *Lgy*LRV1+ parasite dissemination still occurred even when neutrophils, neutrophils and monocytes together, or macrophages were depleted (**Figures 10B**, **E**, **H**). This suggested that albeit neutrophils and monocytes were significant cell type(s) to get infected during the earliest hours of infection, they were most definitely not the sole carrier(s) of *Lgy* parasites for dissemination, individually or together. However, it also signifies the commanding role of resident macrophages (and incoming monocytes) in the establishment of *Lgy* infection, thereby directly impacting disease development, its progression, and thus, its final metastatic outcome as well.

Finally, we infected *Rag2^−/−^γc^−/−^* mice (deficient in B, T, and NK cells) with *Lgy*LRV1+. These mice showed evident parasite burden in the tail, increasing significantly from W4 onwards till the end of infection (**Figure 8F**, tail). However, they developed significantly smaller FP lesions as compared to our metastatic *Ifng^−/−^ Lgy*LRV1+ mice till W9 p.i. and peaked very late between W16–W21 p.i. (**Figure 8F**, FP). They never showed any metastatic nodules or cartilaginous destruction as observed in the *Ifng^−/−^ Lgy*LRV1+ group even during the late phase of chronic infection (**Figure 8F**, tail) even if they showed extensive parasite migration to distant secondary organs such as the tail, forelimb, and snout, by W21 p.i. (**Figure 8G**). Thus, T, B, and NK cells were also not absolutely essential to facilitate metastatic dissemination but probably mediated disease exacerbation and tissue destruction. In conclusion, this confirmed that *Lgy* dissemination could occur even in the absence of the adaptive cells (B, T, and NK cells) (**Figure 8G**) or major innate cells (**Figure 10**) and that dissemination was not limited to the presence of any specific immune cell.

## Discussion

Our study presented a multi-component model of parasite dissemination associated with infectious inflammation, which allowed us to demonstrate inflammation-associated metastatic spread from the site of parasite entry in the skin. Conclusively, our data presented a detailed murine model of metastatic CL that documented the dynamics of early myeloid cell recruitment and their role in the large-scale dissemination of *Lgy* parasites through lymphatics. We identified LNs as major reservoirs of *Lgy* infection and defined extensive routes of their lymphatic drainage in addition to what has been previously described (Harrell et al., 2007; Harrell et al., 2008; Zheng et al., 2014; Wang et al., 2015; Yamaji et al., 2018). While parasite quantification clearly pointed towards LNs failing as efficient firewalls to stop the extensive *Lgy* spread, we established that macrophages on site play most significant roles both in the maintenance and in the resolution of infection, thereby impacting dissemination and overall disease outcome. Albeit heavily infected sites showed a significant presence of non-immune cell-associated extracellular amastigotes, a similar phenotype in the lymphatic SCS of infected LNs and a similar observation through imaging FACS further consolidated our hypothesis that *Lgy* dissemination occurred partly by cell association and also as free amastigotes through the lymph entering the systemic circulation. Thus, parasite dissemination was favored by migrating infected myeloid cells along with extracellular amastigotes sticking to their cell surfaces and amastigote clumps from different draining LNs, moving to the circulatory system. Several immuno-histological studies in clinically affected dogs with different *Leishmania* strains have reported a similar presence of amastigotes, suggestive of hematogenous dissemination of the parasite and tropism for the skin (Solano-Gallego et al., 2004; Mohebali et al., 2011; Ordeix et al., 2017). Remarkably, the presence of a large number of free amastigotes has been described across multiple clinical cases of HIV co-infection with different *Leishmania* strains (Puig and Pradinaud, 2003; Parmentier et al., 2016; Henn et al., 2018) along with free virulent amastigotes for active dissemination (Rosazza et al., 2021). Histopathological studies in humans infected with *Leishmania infantum* have also revealed the presence of free amastigotes in draining LNs and skin (Cobo et al., 2016). Thus, the presence of extracellular amastigotes in draining LNs and blood as observed in our *Ifng^−/−^* mice is not a singular event and even supports recent studies on the mode of dissemination of *S. pyogenes* bacteria, thereby suggesting that infection in *Ifng* ^−/−^ mice could be a very useful model system to study pathogen dissemination in various host–pathogen setup (Bogdan et al., 2000; Puig and Pradinaud, 2003; Solano-Gallego et al., 2004; Mohebali et al., 2011; Cobo et al., 2016; Ordeix et al., 2017; Henn et al., 2018; Rosazza et al., 2021; Siggins et al., 2020).

**Figure 11:**
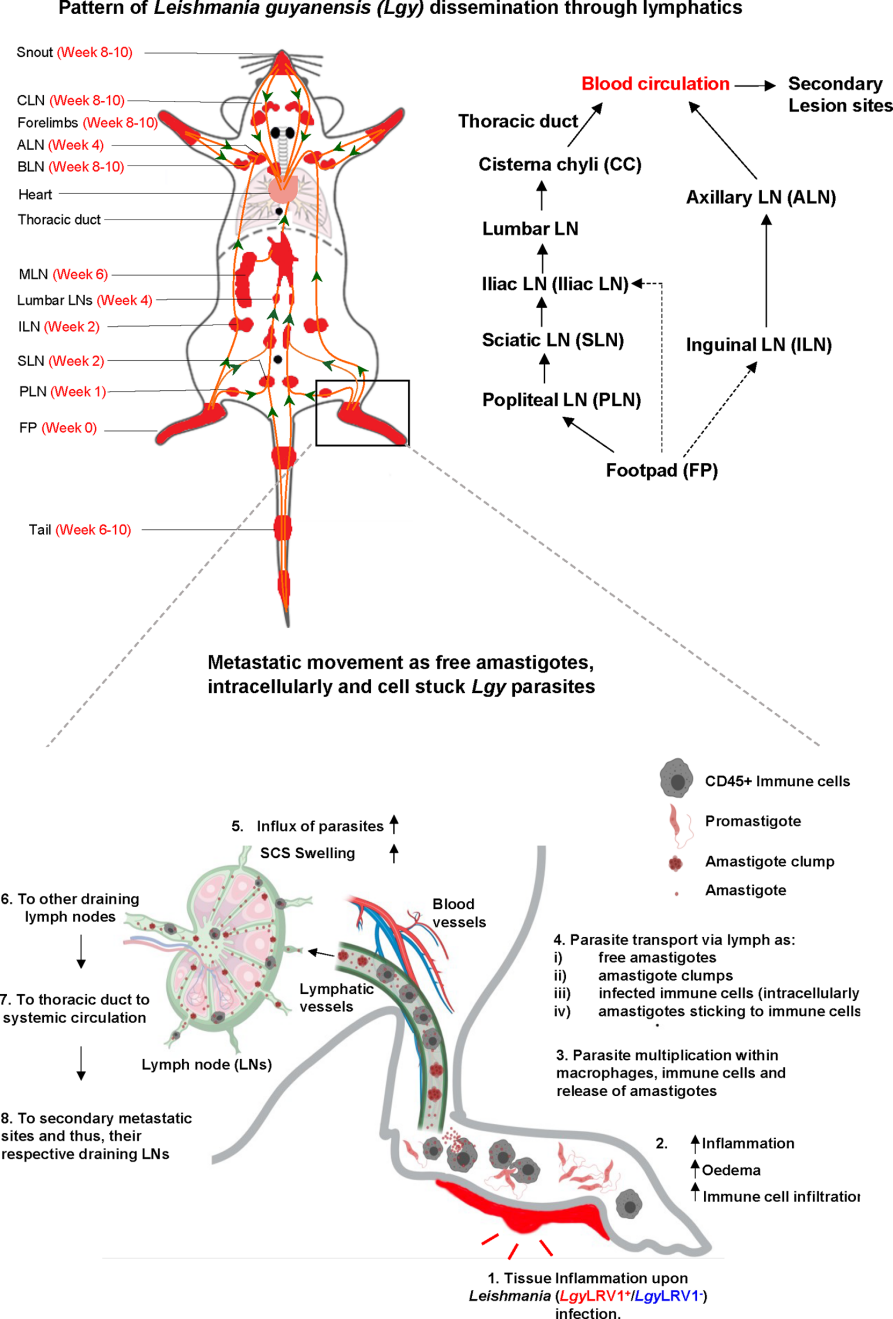
Overall model of dissemination and metastasis of *Lgy* parasites through lymphatics. Representative schematic diagram proposes the model of systemic dissemination of *Lgy* parasites, through sequential infection of mentioned draining LNs following different lymphatic routes, thereby gaining access to the systemic circulation for widespread metastasis in infected *Ifng^−/−^* mouse. The diagram summarizes stepwise events following an FP infection with either *Lgy*LRV1+ or *Lgy*LRV1− metacyclic promastigotes, leading to (1) hyper-inflammation of the infection site, causing the (2) recruitment of various CD45^+^ immune cells that exacerbates the disease. (3) Infection of recruited neutrophils, macrophages, and other immune cells facilitates the transformation and multiplication of amastigotes (from the promastigotes form) within these cells by simple division, which then ruptures to release free amastigotes. (4) Amastigotes move freely or in clumps, either infecting or getting stuck to the surface of different moving immune cells additionally to enter the draining LNs *via* lymph. (5) Influx of amastigotes causes hyper-inflammation of SCS in draining LNs, leading to further exacerbation. (6) Efferent lymphatic collecting vessels carry these parasites (in all four described forms) to infect other draining LNs sequentially in a similar manner. (7) All lymphatic collecting vessels ultimately join the thoracic duct, thereby draining *Lgy* parasites to systemic circulation, which can then (8) disseminate them further to favorable sites for continued multiplication, thereby facilitating large-scale metastases and causing secondary debilitating lesions. Although *Lgy*LRV1− infection displayed a significantly delayed disease phenotype, the sequence of disease progression followed exactly similar pattern of dissemination described.

Although we cannot definitively exclude the possibility that the presence of extracellular amastigotes could be due to a local burst of heavily infected macrophages, observation of sustained numbers of extracellular parasites in blood performed through imaging flow cytometry, showing viable mCherry^+^ amastigotes, strongly favors our proposed hypothesis of parasites dissemination partly as non-cell=bound, extracellular amastigotes. *Lgy* parasites could then be flushed by the blood to selective niches for further metastases, such as to the skin for subsequent transmission by the sand fly. Additionally, complement receptors mediators or proteins (secreted by macrophages or fibroblasts) are upregulated in response to IFN-γ. The surge in complement pathway activity enhances opsonic uptake of extracellular pathogen *via* receptor mediated phagocytosis. So we probably cannot exclude a role of the complement system and that less parasites were internalized as the complement pathway was probably impaired in our model system due to the lack of IFN-γ. This would require further investigation. Additionally, the depletions of major upregulated cell types known to be host cells for *Leishmania*, could not totally block *Lgy* metastatic spread, thereby confirming that none of these heavily trafficked myeloid cells acted as the sole carrier for *Lgy* intracellular transport. While we also do not deny that such exacerbated phenotype observed could be directly associated with our unique *Ifng^−/−^* model, the metastatic outcome in *Rag2^−/−^γc^−/−^ Lgy*LRV1+ infection established that the pro-inflammatory immune response mediated by adaptive cells lead to tissue degradation during chronic infection. Interestingly, dissemination without the formation of debilitating lesions was also observed in *Ifng^−/−^* mice infected with *Lmj*, thereby suggesting that dissemination and lesion formation were two distinct processes. In our model, dissemination occurred with different *Leishmania* species, but lesion formation was species specific as observed in MCL patients and possibly linked to the activity of adaptive cells or hyper-inflammation as described in MCL patients infected with *L. braziliensis* exhibiting CD8^+^T cell-mediated disease pathology (Cardoso et al., 2015; Novais et al., 2017). It also showed that *Leishmania* (*Viannia*) parasites could use different modes of transport and model the lymphatic system to cause secondary lesions and reach transmission sites. Additionally, our data cohesively established the exacerbation rendered by LRV1 in association with *Lgy* parasites, wherein their absence evidently delayed and lowered the overall disease outcome. Collectively, our results suggest that LNs should not be seen only as the site for mediating antigen-specific adaptive response through the egress of specific activated lymphocytes but also as a local canvas supporting parasite capture, multiplication, and dissemination. We therefore concluded that LRV1 could possibly impact cell retention of draining LN, which directly affect the parasite dissemination apart from increased myeloid cell and extracellular amastigote trafficking through the lymphatics. Thus, our proposed model of overall *Lgy* dissemination in metastatic CL has been represented by a schematic diagram for visual reference (**Figure 11**).

## Data availability and Reporting Guidelines

Sample size determination: Sample size used in this this study were not predetermined based on any statistical methods. They were rather chosen based on previous experimental experience, prior studies that showed significant effects with similar sample sizes and standards in the field. This included repetition of any designed experiment at least three independent times or more with similar results/trends, with at least 4-5 number of biological replicates for each condition. For assays in which variability is commonly high, we typically used n>5 while for assays which show low variability, we used n<5 sometimes.

Randomization: Animals were treated randomly before intervention, division for different experiments, tissue collection, and analysis. No formal randomization method such as random number generation were used to assign animals in experimental set ups. However, samples were treated randomly for several ex vivo measures like RT-qPCR or LDA processing and for various *in vitro* experiments.

Criteria of inclusion/exclusion: We did not exclude any data from consideration during analysis of individual experiments. However, while pooling experiments, expected variability in mice studies were defined to exclude outliers, all surgical or accidental deaths were excluded from the analyses. This exclusion was pre-established. Data from contaminated samples were also excluded. This exclusion was pre-established.

Blinding: Investigators were not blinded during the course of experimental follow up or treatment. Blinding during collections were not needed because the conditions were well controlled. However, mice samples were processed ex vivo by technicians without any prior judgement or interpretation. The person performing several sample preparations for immunofluorescence imaging were unaware of the sample identity and collaboration across labs were carried out without giving prior expectations and were confirmed later with similar results.

Replication of experiments: It is provided in the respective Figure legend of each of the given Figure. All attempts at replication were successful with similar results/trends, across multiple experiments. All experiments included sufficient sample size, taking into account the expected variability when performing mice studies. The number of independent experiments, biological replicates for each Figure is indicated in the Figure legend itself. Many data show the aggregation of several independent experiments, while few data like immunofluorescence images and LDA, are from a representative experiment. In case of such representative experiments, the number of independent experiments that reproduced the finding is also indicated in the Figure legends and support conclusions drawn in this report.

ARRIVE rules adherence: Although we stuck to following the prescribed ARRIVE rules to the closest possible in our animal studies, we didn’t officially define it during licensing.

Software availability: All software used for analysis are commercially available and detailed in the “software used for analysis” section, under “methods” section below.

All datasets generated and analyzed are available in the main text or the **Supplementary Materials**; raw sources could be accessible upon request from the corresponding author.

## Data availability statement

The raw data supporting the conclusions of this article will be made available by the authors, without undue reservation.

## Ethics statement

The animal study was reviewed and approved by Swiss Federal Veterinary Office (SFVO).

## Author contributions

Conceptualization: BJ and NF. Methodology: BJ, TP, SL, and NF. Investigation: BJ, CR, MR, FP, FM, LD, CD, AS, LS, SL, and NF. Resources: SL, L-FL, KO, SB, and NF. Writing—original draft: BJ and NF. Writing—review and editing: BJ, NF, SL, TP, AS, and SB. Funding: NF and SB. All authors read and approved the final manuscript.

## Funding

This work is funded by grants from the Swiss National fund for research (http://www.snf.ch/en/Pages/default.aspx) (No. 310030_173180, NF) and the National Institutes of Health (NIH, https://www.nih.gov/) (R01AI-31078 and R01AI-30222-02, SB).

## Acknowledgments

We would like to thank Dr. Luigi Bozzo (Cellular Imaging Facility, CHUV); J.-C. Stehle and Janine Horlbeck (Mouse Pathology Facility); and Dr. Francisco Sala de Oyanguren, Dr. Romain Bedel, and Dr. A. Wilson (Flow Cytometry Facility) for the technical assistance. We also thank Dr. Amel Bekkar for generating the heatmaps using R, Dr. Slavica Masina for critically assessing the manuscript, and Prof. Jean-Daniel Tissot for his support.

## Conflict of interest

The authors declare that the research was conducted in the absence of any commercial or financial relationships that could be construed as a potential conflict of interest.

## Publisher’s note

All claims expressed in this article are solely those of the authors and do not necessarily represent those of their affiliated organizations, or those of the publisher, the editors and the reviewers. Any product that may be evaluated in this article, or claim that may be made by its manufacturer, is not guaranteed or endorsed by the publisher.

## Glossary

**Table:**

Aa	amino acids
Ab(s)	antibody(-ies)
ADCL	anergic diffused cutaneous leishmaniasis
AIDS	acquires immunodeficiency syndrome
APC(s)	antigen-presenting cell(s)
ALN(s)	axillary lymph node(s)
ATP	adenosine triphosphate
B	B-cell zone (in lymph nodes)
B cell(s)	B lymphocyte(s)
BMDM	bone marrow-derived macrophage
BLN(s)	brachial lymph node(s)
Bp (bp)	base pair
CAM	cellular adhesion molecule
CC	cisterna chylae
CD	cluster of differentiation
CD169	lymph node resident macrophages
CLN(s)	superficial cervical lymph node(s)
CLR	c-type lectin receptor
CTR	C-terminal region
DAMP	danger-associated molecular pattern
DAPI	4′
6-diamidino-2-phenylindole	
DC(s)	dendritic cell(s)
DLN(s)	draining lymph node(s)
DKO	double knock-out
DNA	deoxyribonucleic acid
DL	disseminated cutaneous leishmaniasis
dsRNA	double-stranded RNA
ER	endoplasmic reticulum
EB	Evan’s blue
ELISA	enzyme-linked immunosorbent assay
FACS	fluorescence-activated cell sorting
FBS	fetal bovine serum
FDA	Food and Drug Administration
FL	forelimb
FLI	forelimbs injection
FITC	fluorescein isothiocyanate
FP(s)	footpad(s)
Gp63	63 kDa surface glycoprotein of <italic>Leishmania;</italic> gl
ganglion lymphatic	
GST	glutathione <italic>S</italic>-transferase
H	hour (majorly post-infection)
H2O2	hydrogen peroxide
HEPES	N-(2-hydroxyethyl) piperazine-N´-2 ethane sulfonic acid
H2O2	hydrogen peroxide
HIV	human immunodeficiency virus
IF	immunofluorescence
IFN	interferon
IFNGR	IFN gamma receptor
IL	Interleukin
ILN(s)	inguinal lymph node(s)
ILC LN(s)	iliac lymph node(s)
Infl. Monos	inflammatory monocytes
i.p.	intraperitoneal
i.v.	intravenous
iNOS	inducible nitric oxide synthase
iPSC	induced pluripotent stem cell
kDa	kilo Dalton
Kg	kilogram
L. <italic>Leishmania;</italic> LCL	localized cutaneous leishmaniasis
LDA	limiting dilution assay
LCMV	lymphocytic choriomeningitis virus
LFP	left footpad
LPG	lipophosphoglycan
<italic>Lmj	Leishmania major;</italic>
LPS	lipopolysaccharide
LN(s)	lymph node(s)
LRR	leucine-rich repeat
LRV	<italic>Leishmania</italic> RNA virus
MAb	monoclonal antibody
MC	medullary chord
Macros	macrophages
MHC	major histocompatibility complex
MPO	myeloid peroxidase
qRT-PCR	quantitative real-time polymerase chain reaction
ψm	mitochondrial membrane potential
MLV	multilamellar vesicle
mM	millimolar
mRNA	messenger ribonucleic acid
MCL	mucocutaneous leishmaniasis
MEF	mouse embryonic fibroblast
MLN(s)	mesenteric lymph node(s)
Monos	monocytes
MS	medullary sinus
MYA	millions of years ago
MyD88	myeloid differentiation response protein 88
NaN3	modium azide
NaNO2	modium nitrite
NK cells	natural killer cells
NO	nitric oxide
O2-	superoxide
O3	ozone
PAMP	pathogen-associated molecular pattern
P.I./p.i.	post-infection
PBS	phosphate-buffered saline
PC	phosphatidylcholine’
PCR	polymerase chain reaction
PE	phycoerythrin
PKDL	postkala-azar-dermal leishmaniasis
PLN(s)	popliteal lymph node(s)
PRR	pathogen recognition receptor
PRX	peroxiredoxin
PV	parasitophorous vacuole
R (1,2,3,4,5,6,7)	route (1,2,3,4,5,6,7)
RDRP	RNA-dependent RNA polymerase
RFP	right footpad
ROS	reactive oxygen species
RNS	reactive nitrogen species
s.c	sub-cutaneous
S.D	standard deviation
SE	standard error
SCS	sub-capsular sinus
SOD	superoxide dismutase
SLN(s)	sciatic lymph node(s)
ssRNA	single-stranded RNA
T	T-cell zone (in lymph nodes)
T cell(s)	T lymphocyte(s)
TDR	Research and Training in Tropical Diseases
TEMED	NNN¢N¢-tetramethylethylene-diamine
TG	tegumentary leishmaniasis
TGF-β	transforming growth factor beta
Th	T helper
TIR	Toll/IL-1 receptor
TLR	Toll-like receptor
TNF-α	tumor necrosis factor alpha
TTI	tail tip injection
TNFR	TNF receptor
TOSV	Toscana virus
TRAF	TNFR-associated factor
TRIF	TIR domain-containing adapter inducing IFN beta
VL	visceral leishmaniasis
OH	hydroxyl
W/WK	week
WHO	World health Organization
Wt	weight
