# Supplementary material for: In and out: Leishmania metastasis by hijacking lymphatic system and migrating immune cells
Source: Front Cell Infect Microbiol. 2022 Aug 12;12:941860. doi: 10.3389/fcimb.2022.941860 (PMC9414205; doi:10.3389/fcimb.2022.941860)
Supplement: Supplementary file 1 [file DataSheet_1.pdf]

Supplementary Material

Supplemental information includes 6 supplementary Figures, namely **S1 to S5** respectively. Figure S1 represents the schematic diagram showing the location of different LNs in naive *Ifng* $^{-/-}$ mice. Figure S2 represents the schematic diagram comparing kinetics and pattern of parasite dissemination between *Lgy*LRV1+ and *Lgy*LRV1- infections in *Ifng* $^{-/-}$ mice. Figure S3 shows that parasitic dissemination through LNs was not *Lgy* species-specific unlike occurrence of debilitating lesions in *Ifng* $^{-/-}$ *Lmj* infections. Figure S4 represents the schematic diagram of detailed lymphatic drainage in infected *Ifng* $^{-/-}$ mice and thereby explaining our model of dissemination. Figure S5 shows that *Lgy*LRV1+ amastigotes primarily existed in the lymphatic space of infected LNs.

Supplementary Figures (1-5)

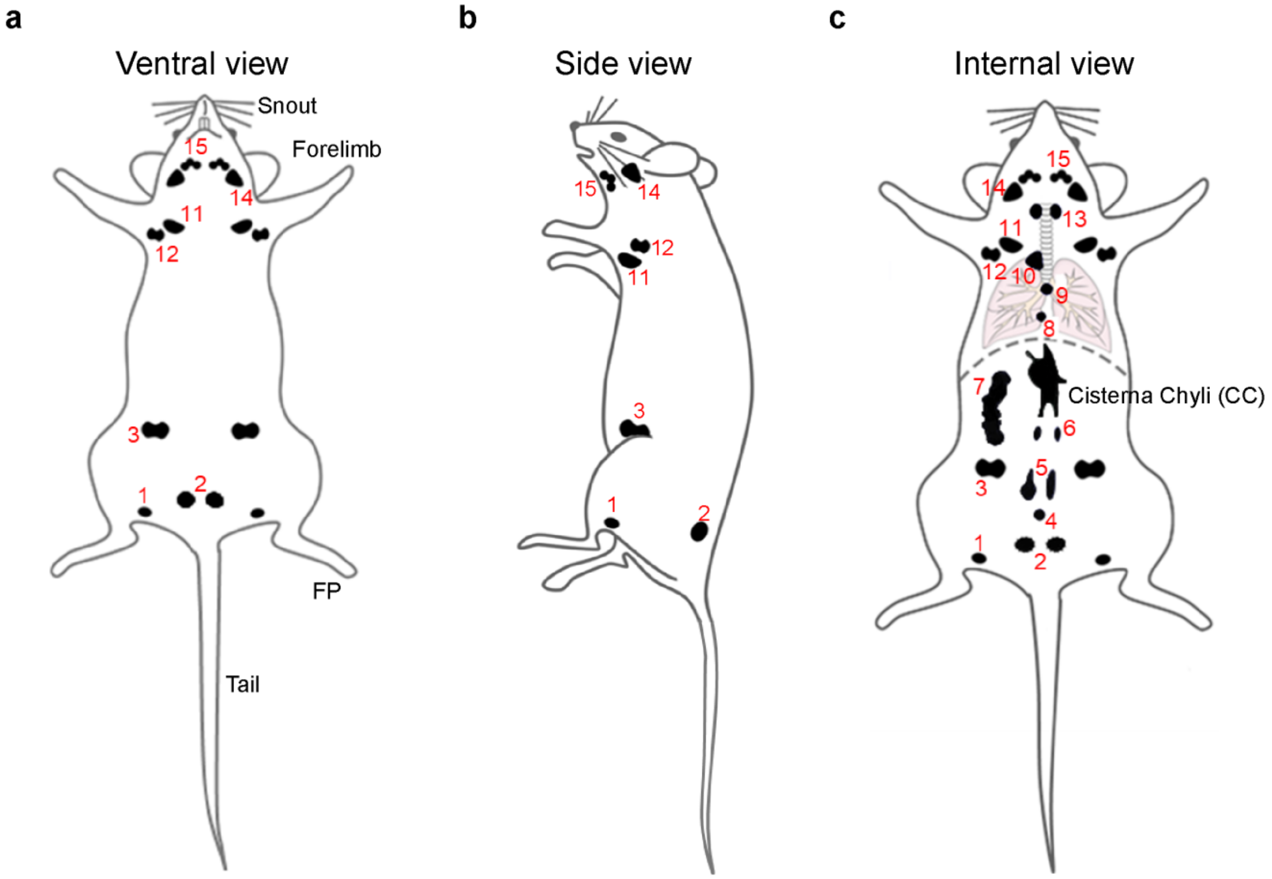

Peripheral LNs
1. Popliteal
2. Sciatic
3. Inguinal
11. Axillary
12. Brachial

Superficial cervical LNs:
14. Parotid
15. Mandibular

Internal abdominal LNs
4. Caudal mesenteric
5. Iliac
6. Lumbar
7. Mesenteric

Internal thoracic LNs
8. Caudal mediastinal
9. Tracheo-bronchial
10. Cranial mediastinal
13. Deep cervical

Figure S1

Schematic diagram showing location of different LNs in naive *Ifng* $^{-/-}$ mice.

(a-c) Naive *Ifng*$^{-/-}$ mice were injected with 5µl Evan's blue at different body-sites to locate the various kind of LNs distributed across the mouse body. Schematic drawings summarize the location of these LNs, numbered (1-15) in the mouse, upon dissection in **a)** ventral view- showing LN numbers 1-3, 11-14, **b)** side view- showing LN numbers 1-3, 11-14 in a lateral plane, and **c)** Internal view- showing all the different LNs from 1-15, encompassing even the deeply located LNs which are visible upon opening the mouse viscera. These LNs are represented in black and categorized amongst- Peripheral LNs, Superficial cervical LNs, Internal abdominal LNs and Internal thoracic LNs, as correlated with the LN numbers represented in the schematic itself. Additionally, these LNs are numbered in context to their respective distances from the FP, for easy reference for the FP infection models described later.

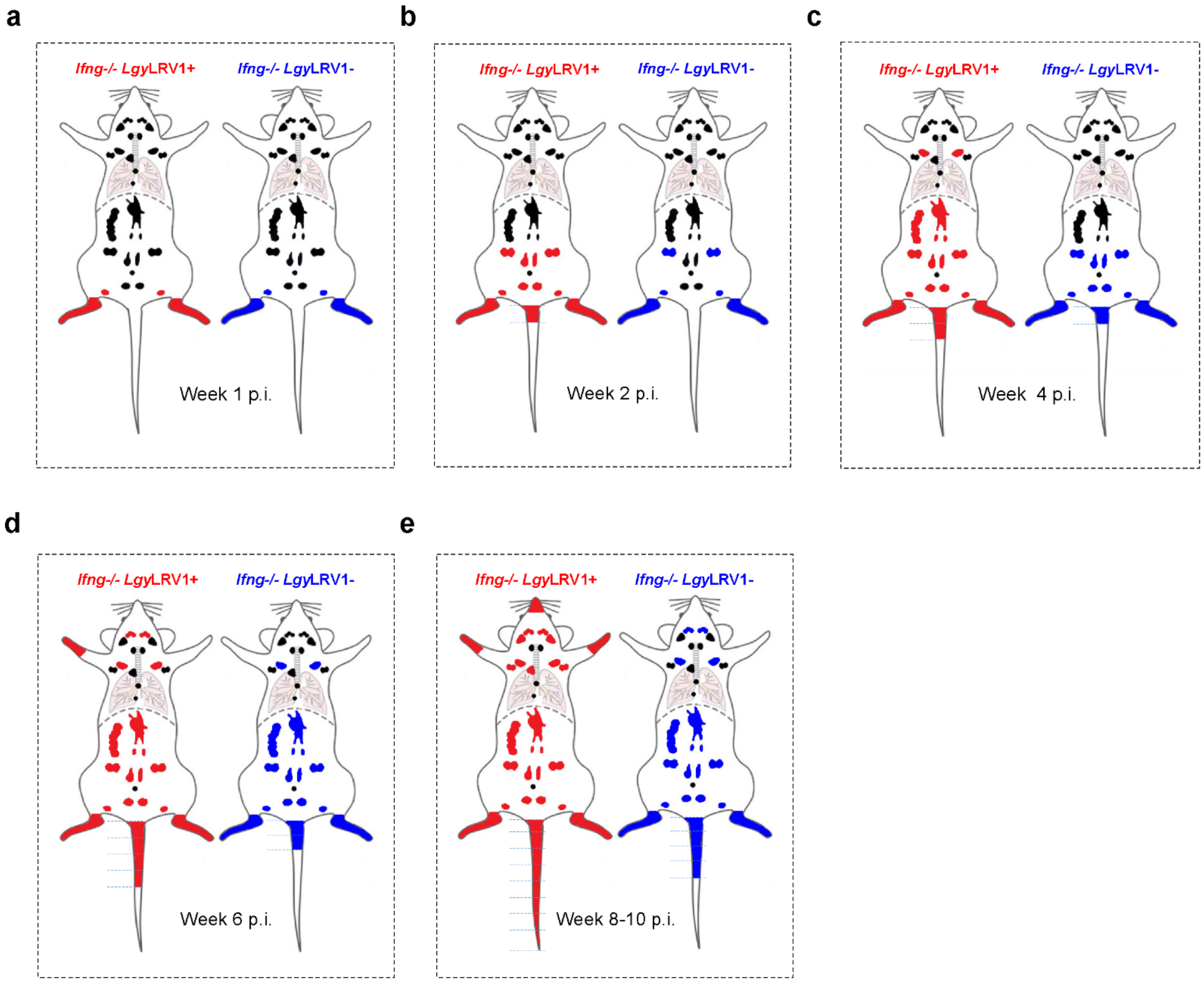

Figure S2

Schematic diagram comparing kinetics and pattern of parasite dissemination between *Lgy*LRV1+ and *Lgy*LRV1- infections in *Ifng* $^{-/-}$ mice.

Infection levels in different organs and LNs in *Ifng*$^{-/-}$ mice subjected to either *Lgy*LRV1+ or *Lgy*LRV1- infections have been represented by colored heat maps and their corresponding quantification in Figure. 1(a-c). Presented schematics **(a-e)** visually represent the same data, comparing the pattern and progression of *Lgy*LRV1+ (in red) or *Lgy*LRV1- (in blue) infections through *Ifng*$^{-/-}$ mice simultaneously, along the course of infection at: **a)** Week 1 p.i. **b)** Week 2 p.i. **c)** Week 4 p.i. **d)** Week 6 p.i, and **e)** Week 8-10 p.i. The black colored LNs represent the same as introduced and numbered previously in Extended Data 2. Black color represents their uninfected state, while their corresponding red or blue marking represents infection by *Lgy*LRV1+ or *Lgy*LRV1- respectively, for visual comparison of progression of infection across the mouse body in each group, at specified time points.

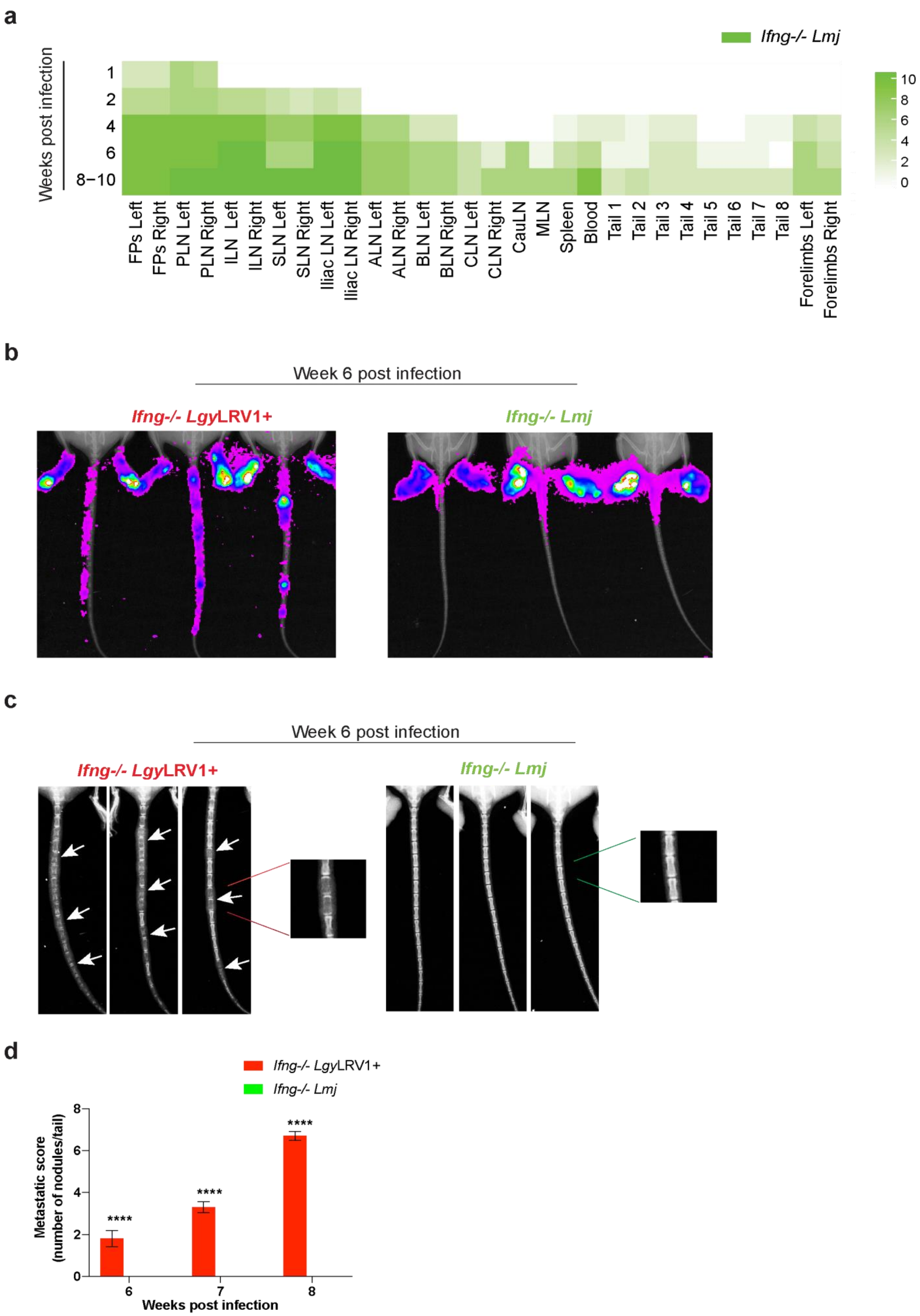
a
Ifng-/- Lmj
Weeks post infection
1
2
4
6
8−10
FPs Left
FPs Right
PLN Left
PLN Right
ILN Left
ILN Right
SLN Left
SLN Right
Iliac LN Left
Iliac LN Right
ALN Left
ALN Right
BLN Left
BLN Right
CLN Left
CLN Right
CauLN
MLN
Spleen
Blood
Tail 1
Tail 2
Tail 3
Tail 4
Tail 5
Tail 6
Tail 7
Tail 8
Forelimbs Left
Forelimbs Right
10
8
6
4
2
0
b
Week 6 post infection
Ifng-/- LgyLRV1+
Ifng-/- Lmj
c
Week 6 post infection
Ifng-/- LgyLRV1+
Ifng-/- Lmj
d
Ifng-/- LgyLRV1+
Ifng-/- Lmj
Metastatic score
(number of nodules/tail)
8
6
4
2
0
****
****
****
6
7
8
Weeks post infection

Figure S3

Figure S3 (continued)

Parasitic dissemination through LNs was not *Lgy* species-specific unlike occurrence of debilitating lesions.

(a) Aforementioned cell suspension of each organ and LNs (collected as left and right), were subjected to limiting dilution analysis (LDA) from *Ifng*$^{-/-}$ mice infected in both hind FP with $1x10^6$ *Lmj-* stationary-phase metacyclic promastigotes at week 1, 2, 4, 6 and 8-10 weeks p.i. Corresponding heatmaps were generated using the R ComplexHeatmap package for **a)** *Ifng*$^{-/-}$ *Lmj* group in green, along the course of infection. The color gradient represented in the heatmaps are correlated to the number of + signs assigned in terms of infection in progressive dilution series observed through LDA for each organ, where increased color intensity corresponded to higher parasite load. **(b, c)** representative images of Tail comparing *Ifng*$^{-/-}$ *Lgy*LRV1+ group with *Ifng*$^{-/-}$ *Lmj* group at W6 p.i showing **b**) inflammation measured in terms of myeloid peroxidase activity, by parasite bioluminescence; **c**), x-ray pictures showing the appearance of metastatic nodules nested with cartilaginous destruction, pointed by white arrow-heads; **d)** metastatic score quantified by absolute count of the number of nodules per tail comparing *Ifng*$^{-/-}$ *Lgy*LRV1+ with *Ifng*$^{-/-}$ *Lmj* group. All data are representative of one experiment with $n \geq 5$ mice /group/ time point, and repeated at least 5 times independently with similar result. Statistics are shown as mean ± SEM with p values determined using two-way ANOVA with Bonferroni's post-test for d. Stars of significance are determined as $*p < 0.05$; $**p < 0.01$; $***p < 0.001$; $****p < 0.0001$.

a

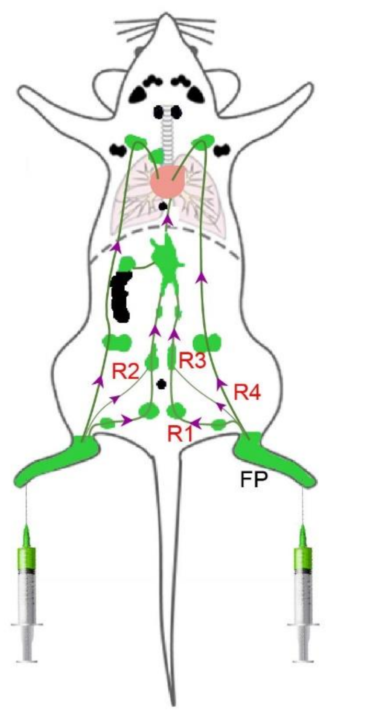

Lymphatic connections observed after both foot pad injection of FITC-Dextran

b

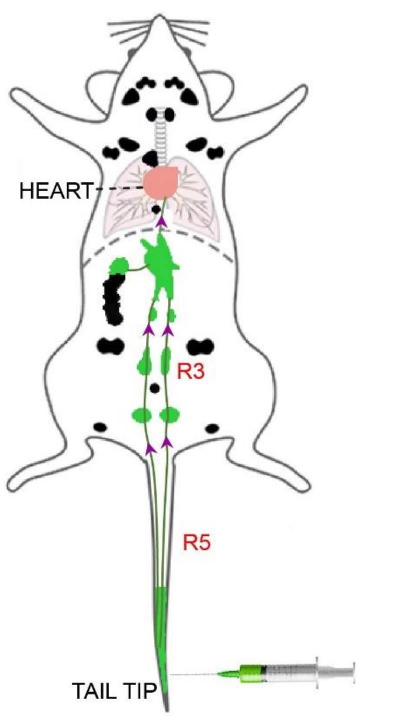

Lymphatic connections observed after tail tip injection of FITC-Dextran

c

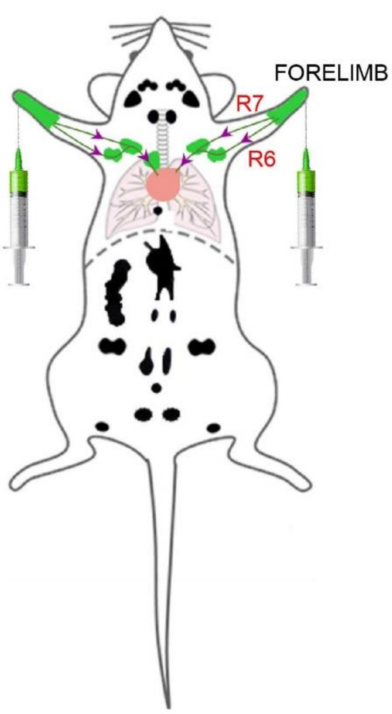

Lymphatic connections observed after both fore limb injection of FITC-Dextran

d

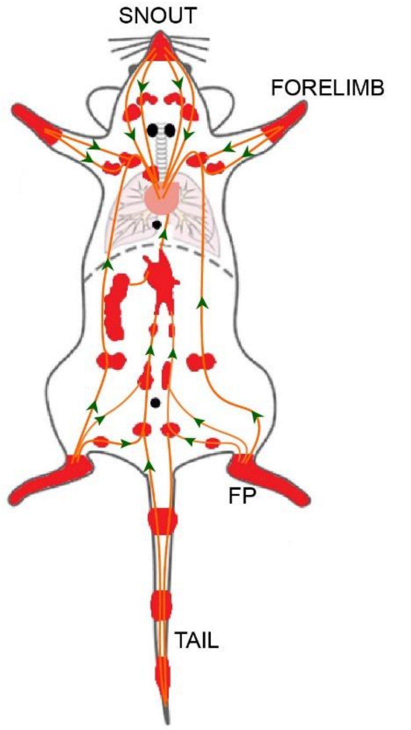

Overall pattern of dissemination in our model of infected *Ifng-/- Lgy*LRV1+ or *Ifng-/- Lgy*LRV1- mice

Figure S4

Figure S4 (continued)

Schematic diagram of detailed lymphatic drainage in infected *Ifng* $^{-/-}$ mice.

The different routes of lymphatic drainage observed in an infected *Ifng*$^{-/-}$ *Lgy*LRV1+ group has been shown in Figure. 2 (a-c). Presented schematics **(a-d)** visually summarizes the same data, comparing the different lymphatic connections observed through fluorescent stereo-microscopic lymphangiography in infected *Ifng*$^{-/-}$ mice, following a 5ul injection of FITC- Dextran individually in **a)** both FP: showing Routes(R) - R1, R2, R3 and R4; **b)** tail tip: revealing R3 and R5; and **c)** both forelimb: showing R7 and R8 of lymphatic drainage. **d)** Taken together, these connections reveal the overall lymphatic routes followed by the *Lgy* parasites (both *Lgy*LRV1+ and *Lgy*LRV1-) to ultimately drain into the circulatory system and gain access to the blood for systemic dissemination to secondary sites like forelimb, tail and snout, as represented in **d**. R1 = FP to PLN to SLN to Iliac ventrally, R2 = FP to Iliac LN, R3 = iliac LN to CC to MLN to thoracic duct, R4 = FP to ILN to ALN to subclavian vein, R5 = tail tip upwards to the SLN to Iliac LN ventrally, R6 = forelimb to BLN to ALN draining into subclavian vein, and R7 = forelimb drainage to ALN to subclavian vein. The different arrowheads show the direction of lymphatic drainage, collecting from a mentioned organ and draining into another organ (mostly LNs) pointed to.

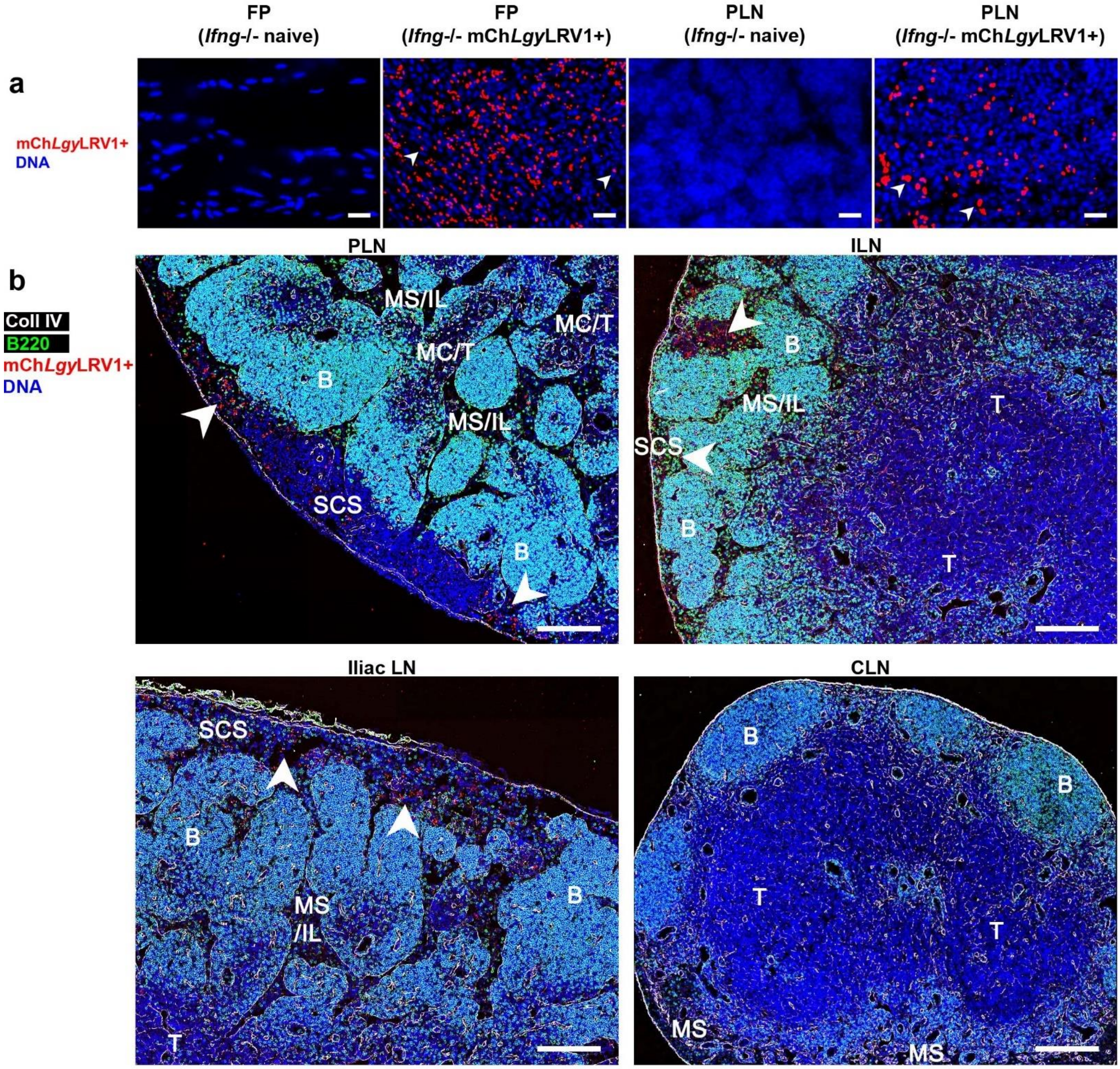

Figure S5

***Lgy*LRV1+ amastigotes primarily existed in the lymphatic space of infected LNs.**

(a-b) Naive *Ifng*$^{-/-}$ mice (previously injected with DPBS in both hind FP) and *Ifng*$^{-/-}$ mice infected in both FP with $1x10^6$ mCherry expressing mch*Lgy*LRV1+ promastigotes at week 2 p.i., were euthanized to obtain LN types and FP and subjected to histological staining as described previously. Representative images acquired through slide scanner shows the histological staining of mentioned sections with **a)** DAPI (staining DNA) and anti-mCherry staining on FP and PLN from naive *Ifng*$^{-/-}$ mice and *Ifng*$^{-/-}$ mice infected with mch*Lgy*LRV1+ parasites at W2 p.i for comparison (scale bar- 20um); **(b)** cryostat sections of major infected LNs- PLN, ILN, Iliac LN and CLN (as negative control), stained with DAPI, anti-mCherry, B220 and Collagen IV (Coll IV) labels for staining DNA, mCh*Lgy*LRV1+ parasites, B cells and the basement membrane of vessels and its conduits, respectively. Representative overview images are provided to show mCherry+*Lgy*LRV1+ distribution in these LNs (scale bar- 200um). (a-b) white arrow heads points to the presence of

Figure S5 (continued)
mCherry+ *Lgy*LRV1+. All data are representative of 2-3 experiments with at least 2 mice (n ≥ 2 mice /group) and 6 LNs/ mouse. SCS=Sub-capsular Sinus, B/T= B/ T cell zone in LNs, MC/MS/IL= Medullary cord/ Medullary sinus/intranodal lymphatics, respectively.
