# Supplementary material for: In and out: Leishmania metastasis by hijacking lymphatic system and migrating immune cells
Source: Front Cell Infect Microbiol. 2022 Aug 12;12:941860. doi: 10.3389/fcimb.2022.941860 (PMC9414205; doi:10.3389/fcimb.2022.941860)
Supplement: Supplementary file 2 [file DataSheet_2.pdf]

Table S1. Antibodies, dyes and beads used in this study

Target	Clone name	Coupling	Dilution	Brand	Lot	Reference	Methods
Anti-mouse Ly6C	HK1.4	FITC	1:300	BioLegend	B195146	Cat. #128006	Flow cytometry and Immunofluorescence analysis
Anti-mouse/human CD11b	M1/70	PE-Cyanine 5	1:1000	BioLegend	B178549	Cat: #101210	Flow cytometry and Immunofluorescence analysis
Anti-mouse F4/80	BM8	APC-Cyanine7	1:100	BioLegend	B324923	Cat: #123118	Flow cytometry and Immunofluorescence analysis
Anti-mouse CD45.2	104	Brilliant Violet 510	1:500	BioLegend	B251593	Cat. #109837	Flow cytometry and Immunofluorescence analysis
Anti-mouse CD192 (CCR2)	SA203G11	Alexa fluor 647	1:100	BioLegend	B239629	Cat. #150604	Flow cytometry and Immunofluorescence analysis
Anti-mouse CD206 (MMR)	C068C2	Biotin	1:200	BioLegend	B275399	Cat. #141714	Flow cytometry and Immunofluorescence analysis
Anti-Biotin	-	Streptavidin PE-Cyanine7	1:200	BioLegend	B345298	Cat. #405206	Flow cytometry and Immunofluorescence analysis
Anti-mouse CD19	1D3/CD19	FITC	1:500	BioLegend	B243569	Cat. #152404	Flow cytometry and Immunofluorescence analysis
Anti-mouse CD3	17A2	Brilliant violet 510	1:200	BioLegend	B170040	Cat. #100233	Flow cytometry and Immunofluorescence analysis
Anti-mouse CD11c	N418	APC	1:500	BioLegend	B262130	Cat. #117310	Flow cytometry and Immunofluorescence analysis
Anti-mouse I-A/I-E	M5/114.15.2	Brilliant violet 510	1:1000	BioLegend	B172398	Cat. #107635	Flow cytometry and Immunofluorescence analysis
Anti-mouse NK-1.1 (CD161)	PK136	PE	1:200	BioLegend	B134095	Cat. #108708	Flow cytometry and Immunofluorescence analysis
Anti-mouse Ly6G	1A8	APC/Cy7	1:700	BioLegend	B238265	Cat. #127624	Flow cytometry and Immunofluorescence analysis
Anti-mouse/human CD11b	M1/70	Brilliant Violet 421	1:600	BioLegend	B227047	Cat. #101235	Flow cytometry and Immunofluorescence analysis
Anti-mouse CD206 (MMR)	C068C2	Brilliant violet 650	1:500	BioLegend	B229790	Cat. #141723	Flow cytometry and Immunofluorescence analysis
Anti-Biotin	-	Streptavidin PerCP	1:500	BioLegend	B214631	Cat. #405213	Flow cytometry and Immunofluorescence analysis

Target	Clone name	Coupling	Dilution	Brand	Lot	Reference	Methods
Zombie Aqua Fixable Viability Kit	Stains eukaryotic cells	Emission range-512/526nm	1:1000	BioLegend	B226206	Cat: #423102	Flow cytometry.
Aqua fluorescent reactive dye*for 405 nm excitation*	Stains eukaryotic cells	Emission range-367/526	1:250	Invitrogen Molecular probes	430671	Cat: #L34957	Flow cytometry
Anti-mouse Ly6G (Gr-1)	1A8-Ly6G	PE	1:600	eBioscience	4284862	Cat #12-9668-82	Flow cytometry
Anti-mouse CD45	30-F11	PE-Cyanine 7	1:200	eBioscience	4277984	Cat #25-0451-82	Flow cytometry
Anti-mouse CD11c	N418	efluor 660	1:500	eBioscience	E15733-102	Cat #50-0114-82	Flow cytometry
Anti-mouse CD3e	145-2C11	PE-Cyanine 5	1:100	eBioscience	E15711-596	Cat #15-0031-82	Flow cytometry
Anti-mouse CD4	RM4-5	FITC	1:200	eBioscience	E00083-1631	Cat #11-0042-85	Flow cytometry
Anti-mouse Ly6C (Gr-1)	HK1.4	Alexa Fluor 488	1:300	eBioscience	4314544	Cat #53-5932-82	Flow cytometry
Rat Anti-mouse/human CD115 (CSF-1R)	T38-320	PE	1:100	BD Pharmingen	7111867	Cat #565249,	Flow cytometry and Immunofluorescence analysis
DAPI (4',6-Diamidino-2-Phenylindol, Dihydrochloride)	Subcellular localization- nucleus	Emission range-456/460 nm	1:1000	Invitrogen	WB3208041	Cat# D1306	Flow cytometry and Immunofluorescence analysis
Fluoroshield with DAPI F6057-20ml	Subcellular localization- nucleus	Emission range-456/460 nm	Used directly- a drop	Sigma-Aldrich	SLBS2101V	Cat #1002339026	Histology mounting medium
Fluoroshield F6182-20ml	Subcellular localization- nucleus	Emission range-456/460 nm	Used directly- a drop	Sigma-Aldrich	MKCC2089	Cat #1002409107	Histology mounting medium
Onecomp eBeads	Compensation beads	full spectrum cell analysis beads	1 drop (50 µL)/test	eBioscience	4299596	Cat # 01-1111-42	Flow cytometry
Onecomp eBeads	Compensation beads	full spectrum cell analysis beads	1 drop (50 µL)/test	Invitrogen	19214193	Cat # 01-1111-42	Flow cytometry

Target	Clone name	Coupling	Dilution	Brand	Lot	Reference	Methods
BD calibrate Unlabeled Beads	set fluorescence compensation	Any fluorophore as per need	1 drop (50 µL)/test	BD	-	Cat# 340486	Flow cytometry
Rabbit polyclonal antibody to mCherry	-	mCherry	1:500	Abcam	GR3213077-6	Cat # ab 167453	Western blot, Flow cytometry and Immunofluorescence analysis.
Mouse Anti-ds RNA mAb J2 (IgG2a)*	(IgG2a)	-	1:500	English & Scientific consulting Kft	J2-1904	Cat# 10010200	Western blot, Flow cytometry and Immunofluorescence analysis
Affinity purified Goat polyclonal anti- mouse LYVE-1	LYVE-1	-	1:200	R&D Systems	UBZ0418121	Cat# AF 2125	Western blot and Immunofluorescence analysis
Purified Rat monoclonal anti mouse CCR2	CD192	-	1.200	R&D Systems	CDOA0418101	Cat# MAB55381	Western blot, Flow cytometry and Immunofluorescence analysis
Armenian Hamster-anti-CD3e+	Monoclonal CD3e	Primary antibody	1:500	BioXCell	Received as gift	Received as gift	Histology & Immunofluorescence analysis
Rat-anti-CD31/PECAM-1+	Monoclonal EPR17259	Primary antibody	1.300	Home-made	Received as gift	Received as gift	Histology & Immunofluorescence analysis
Rabbit -anti mouse -Lyve1+	Monoclonal RM103	Primary antibody	1:250	RELIATech	Received as gift	Received as gift	Histology & Immunofluorescence analysis
Syrian-Hamster-anti mouse -gp38+	Monoclonal 8.1.1	Primary antibody	1:500	BioXCell	Received as gift	Received as gift	Histology & Immunofluorescence analysis
Armenian-hamster anti-CD11c+	Monoclonal N418	Primary antibody	1:100	Home-made	Received as gift	Received as gift	Histology & Immunofluorescence analysis
Rabbit - anti-Laminin+	Monoclonal A5	Primary antibody	1:300	Sigma-Aldrich	Received as gift	Received as gift	Histology & Immunofluorescence analysis
Rat -anti mouse-CD11b+	Monoclonal M1/70	Primary antibody	1:250	Home-made	Received as gift	Received as gift	Histology & Immunofluorescence analysis
Rat -anti mouse-CD169+	Monoclonal 5F 1.1	Primary antibody	1:200	Thermo Fisher	Received as gift	Received as gift	Histology & Immunofluorescence analysis
Rat-a-CD45pan+	Monoclonal 2D1	Primary antibody	1:500	Home-made	Received as gift	Received as gift	Histology & Immunofluorescence analysis

Target	Clone name	Coupling	Dilution	Brand	Lot	Reference	Methods
Mouse-anti-CD45.2 Biotin	Monoclonal (AL-1)+	Primary antibody	1:500	Home-made	Received as gift	Received as gift	Histology & Immunofluorescence analysis
Mouse anti-NIMP-R14+	Monoclonal NIMP-R14+	Primary antibody	1:500	Adipogen	Received as gift	Received as gift	Histology & Immunofluorescence analysis
Mouse anti-LYVE-1+	Monoclonal 11A9.1	Primary antibody	1:200	R&D systems	Received as gift	Received as gift	Histology & Immunofluorescence analysis
Goat anti-mouse–Collagen IV+	Monoclonal CB243	Primary antibody	1:500	Southern Biotech	Received as gift	Received as gift	Histology & Immunofluorescence analysis
Rat- anti mouse-CD45R/B220+	RA3-6B2	Primary antibody	1:1000	Home-made	Received as gift	Received as gift	Histology & Immunofluorescence analysis
Rat anti-mouse B220+	RA3-6B2	Primary antibody	1:250	Life Technologies	Received as gift	Received as gift	Histology & Immunofluorescence analysis

*200µg reconstitution with 0.25ml distilled H_20

+ Antibody received as gift from collaborators

Table S2. Antibodies used for *in vivo* mice depletion

Target	Clone name	Brand	Lot	Reference	Methods	Isotype control suggested by manufacturer
Purified InVivoMAb anti-mouse Ly6G	1A8	BioXcell	626716O1	Cat #BE0075-1	Neutrophil Depletion	IgG Isotype control: Rat IgG2a, BioXcell
InVivoMAb anti-mouse Ly6G/Ly6C (Gr-1)	RB6-8C5	BioXcell	594416O1	Cat #BE0075	Simultaneous neutrophil and monocyte depletion	IgG Isotype control: Rat IgG2b, BioXcell.
InVivo MAb anti-mouse CSF1R (CD115)	AFS989	BioXcell	661317N1	Cat #BE0213	Macrophage and monocyte depletion	Isotype control: Rat IgG2a, BioXcell
InVivoMAb Rat IgG2a Isotype control, anti Trinitrophenol (Isotype Control A)	2A3	BioXcell	654817M2	Cat #BE0089	Neutrophil Depletion<br><br>Macrophage and monocyte depletion	-
InVivoMAb Rat IgG2ab Isotype control, anti KLH (Isotype Control B)	LTF-2	BioXcell	629817F1	Cat #BE0090	Simultaneous neutrophil and monocyte depletion	-
