# Supplementary material for: In and out: Leishmania metastasis by hijacking lymphatic system and migrating immune cells
Source: Front Cell Infect Microbiol. 2022 Aug 12;12:941860. doi: 10.3389/fcimb.2022.941860 (PMC9414205; doi:10.3389/fcimb.2022.941860)
Supplement: Supplementary file 3 [file DataSheet_3.pdf]

Supplementary Materials 1: Gating strategy for Panel 1 for FACS

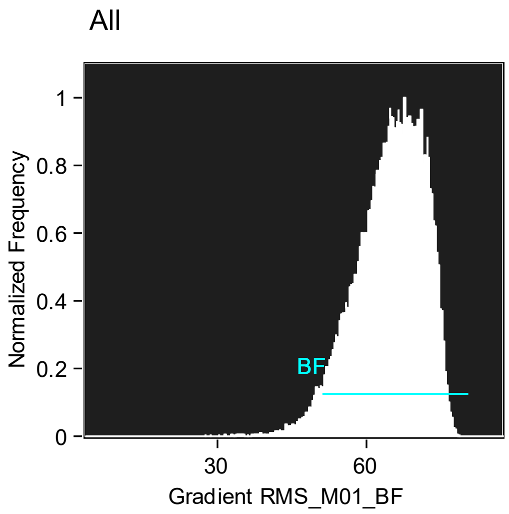

Gradient RMS_M01_BF

Population	Count	%Gated
All	100000	100
BF	96376	96.4

	Ch01	Ch02	Ch03	Ch04	Ch05	Ch06	Ch07	Ch08	Ch09	Ch10	Ch11	Ch12
Ch01	1	0.029	0.045	0.075	0.036	0.02	0.018	0	0	0	0.009	0.023
Ch02	0.042	1	0.142	0.088	0.035	0.04	0.113	0	0	0	0.043	0.179
Ch03	0	0.131	1	0.335	0.097	0.449	0.106	0	0.021	0	0.039	0.183
Ch04	0	0.054	0.455	1	0.056	0.203	0.092	0	0	0	0.033	0.14
Ch05	0	0.018	0.176	0.695	1	0.125	0.089	0	0	0	0.148	0.19
Ch06	0.013	0.024	0.054	0.135	0.138	1	0.059	0	0	0	0.025	0.274
Ch07	0.042	0.01	0.015	0.017	0.024	0	1	0.484	0.014	0	0.049	0.109
Ch08	0	0.021	0.015	0.018	0.024	0	0.426	1	0.018	0	0.042	0.068
Ch09	0	0.005	0.113	0.01	0.024	0.004	0.085	0.289	1	0	0.031	0.027
Ch10	0	0.003	0.051	0.021	0.019	0.001	0.052	0.197	0.029	1	0.026	0.026
Ch11	0	0.003	0.027	0.027	0.907	0.004	0.05	0.09	0.014	0	1	0.251
Ch12	0	0.001	0.01	0.007	0.133	0.129	0.065	0.055	0.013	0	0.154	1

Gradient RMS: measures the sharpness of an image and is useful for the selection of focused objects.

Panel 1

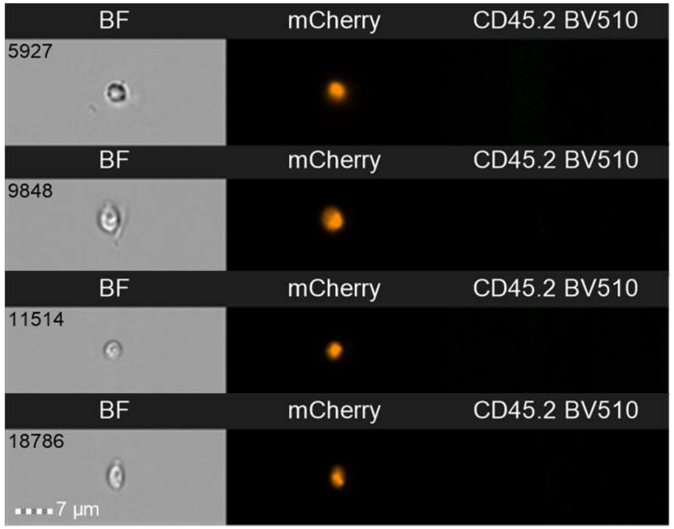

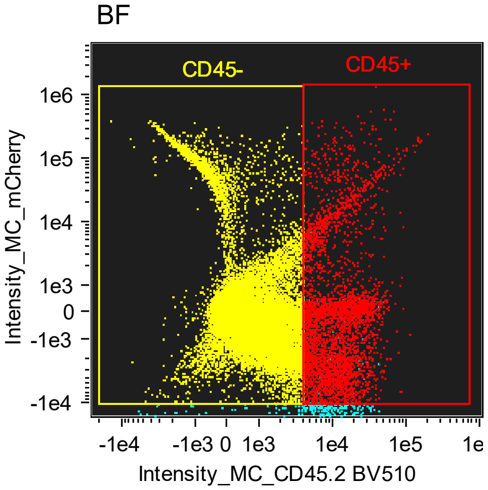

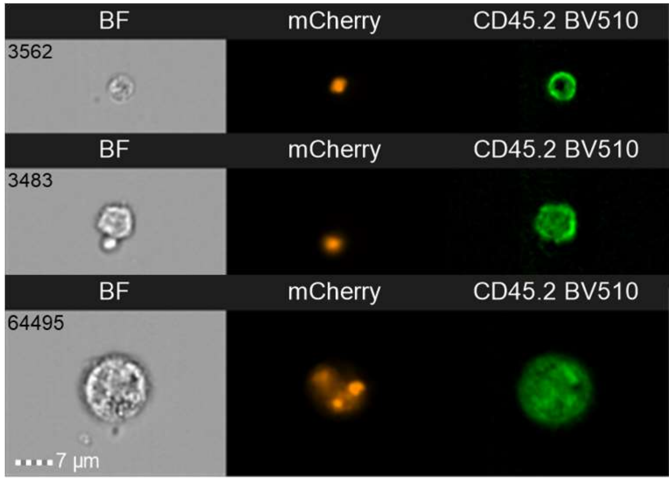

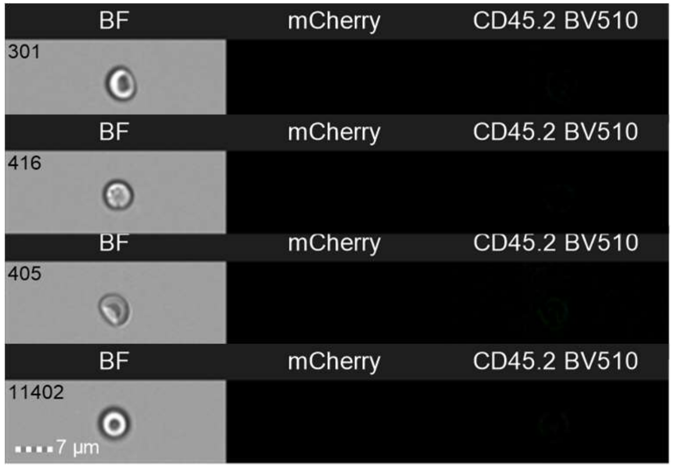

Intensity_MC_CD45.2 BV510, Intensity_MC_mCherry

Population	Count	%Gated
**BF**	96376	100
**CD45- & BF**	91820	95.3
**CD45+ & BF**	4140	4.3

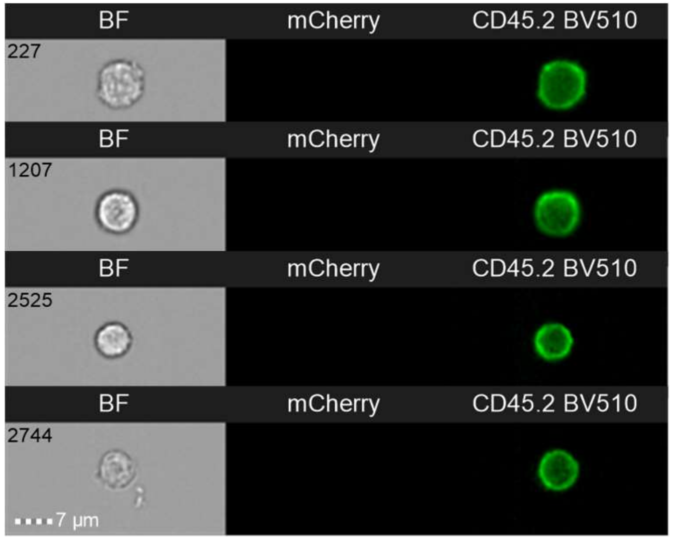

Panel 1

(TER119- CD45- free parasites)

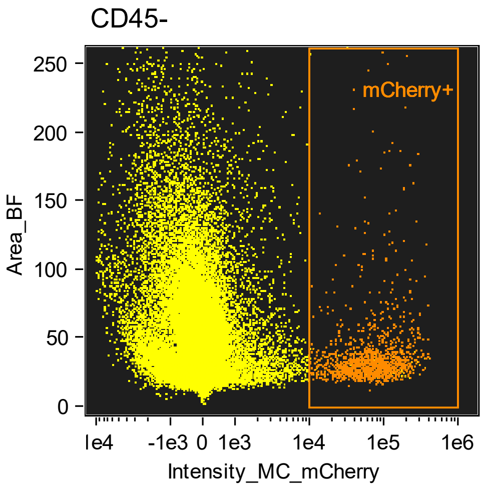

Intensity_MC_mCherry, Area_BF

Population	Count	%Gated
**CD45- & BF**	91820	100
**mCherry+ & CD45- & BF**	1470	1.6

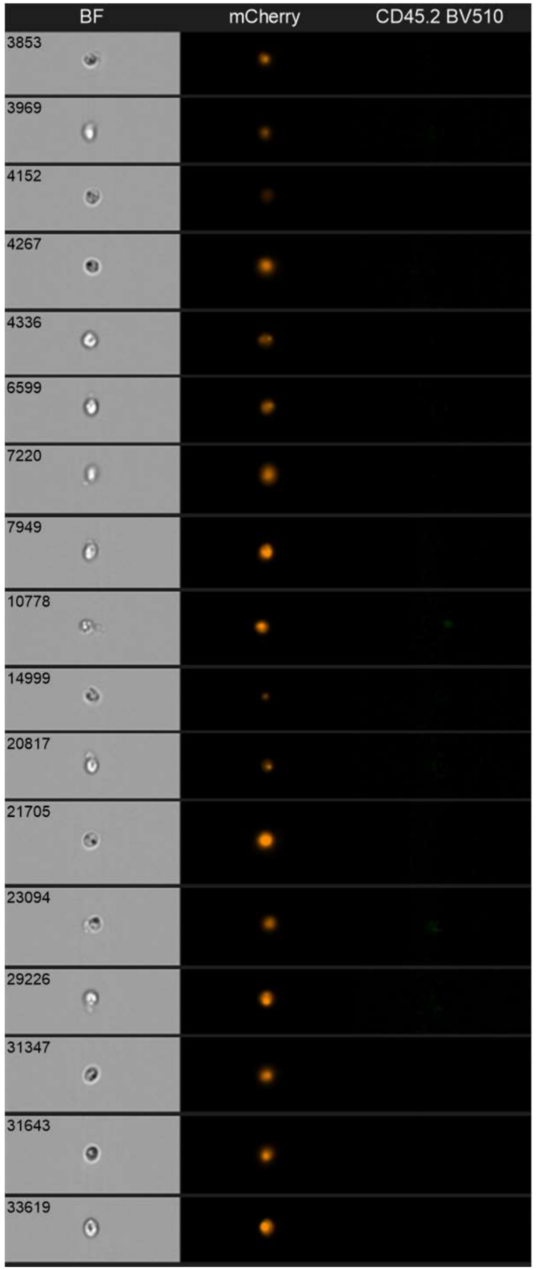

Panel 1
(gated from TER119- CD45+)

Aspect ratio=0.93

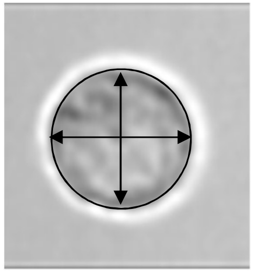

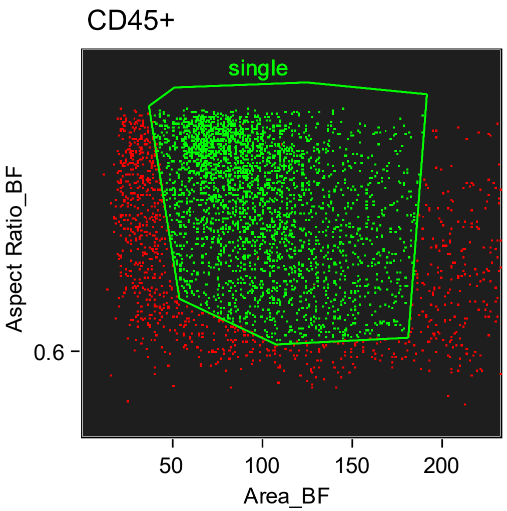

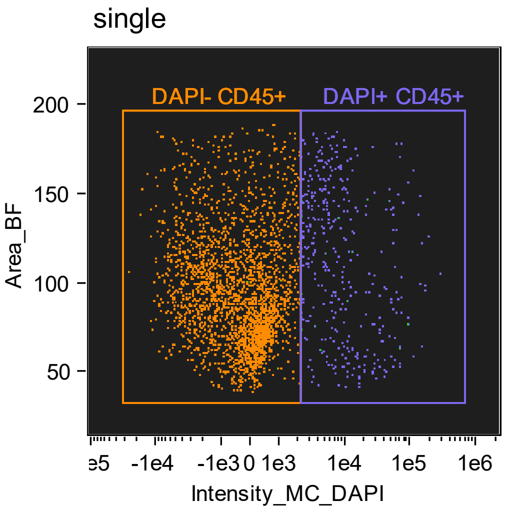

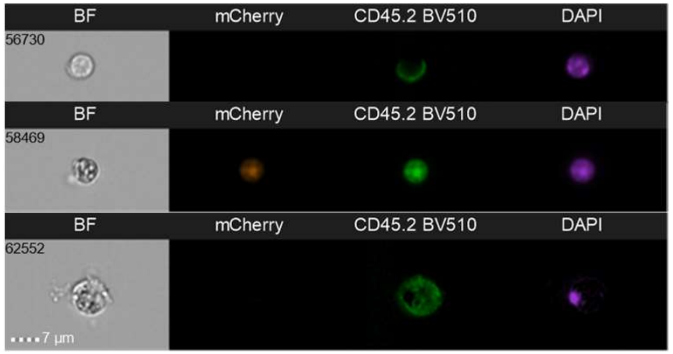

Aspect ratio=0.45

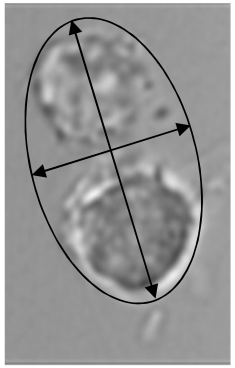

Area_BF, Aspect Ratio_BF

Population	Count	%Gated
CD45+ & BF	4140	100
single & CD45+ & BF	2894	69.9

Intensity_MC_DAPI, Area_BF

Population	Count	%Gated
single & CD45+ & BF	2894	100
DAPI- CD45+ & single & CD45...	2511	86.8
DAPI+ CD45+ & single & CD45...	379	13.1

Single cells are located by plotting the **area** of the bright field image vs. the **aspect ratio** (the ratio of the width and height of the object, i.e. the shape) of the bright field channel.

Panel 1

(gated from DAPI-CD45+)

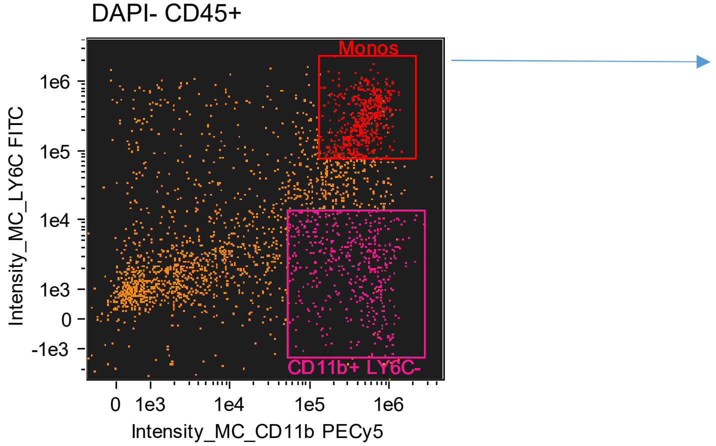

Intensity_MC_CD11b PECy5, Intensity_MC_LY6C FITC

Population	Count	%Gated
DAPI- CD45+ & single & CD45...	2511	100
Monos & DAPI- CD45+ & singl...	541	21.5
CD11b+ LY6C- & DAPI- CD45+ ...	464	18.5

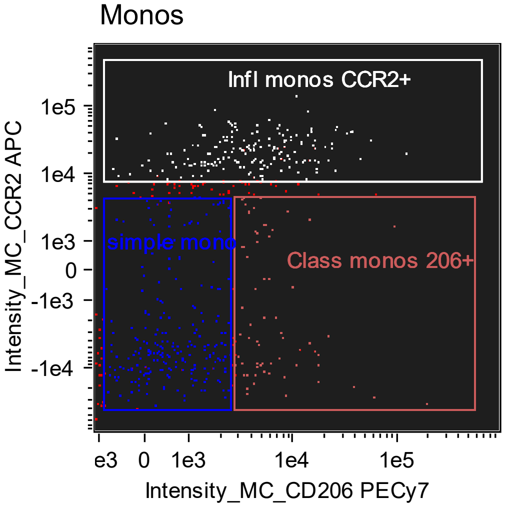

Intensity_MC_CD206 PECy7, Intensity_MC_CCR2 APC

Population	Count	%Gated
Monos & DAPI- CD45+ & singl...	561	100
simple mono & Monos & DAPI-...	221	39.4
Infl monos CCR2+ & Monos & ...	179	31.9
Class monos 206+ & Monos & ...	65	11.6

Panel 1

Inflammatory Monocytes (Ly6G-Ly6C+CD11b+ CD206- CCR2+)

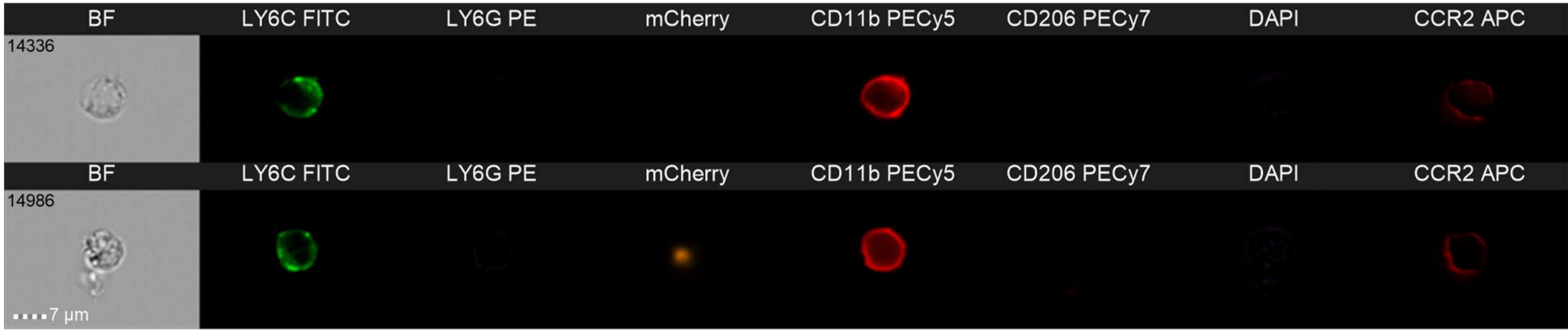

Classical Monocytes (Ly6G-Ly6C+CD11b+ CD206- CCR2-)

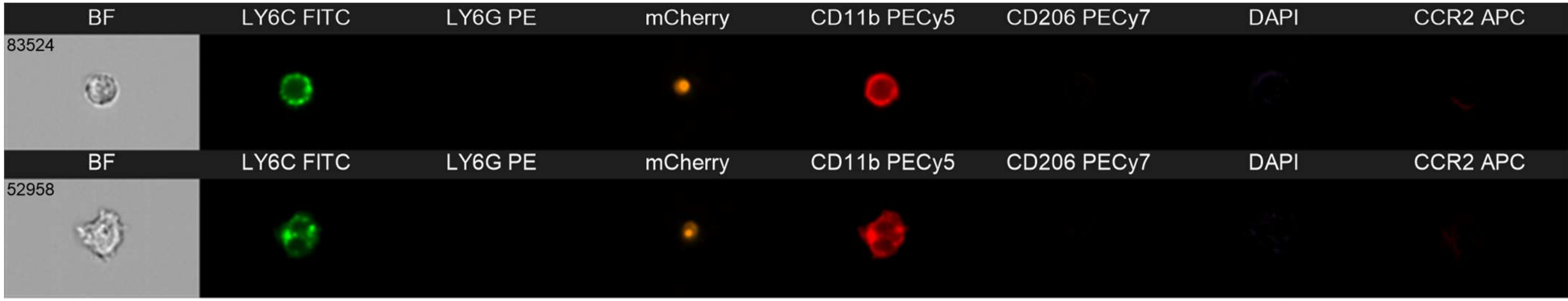

Macrophage/monocyte with Mannose Receptor (Ly6G-Ly6C+CD11b+ CD206+ CCR2-)

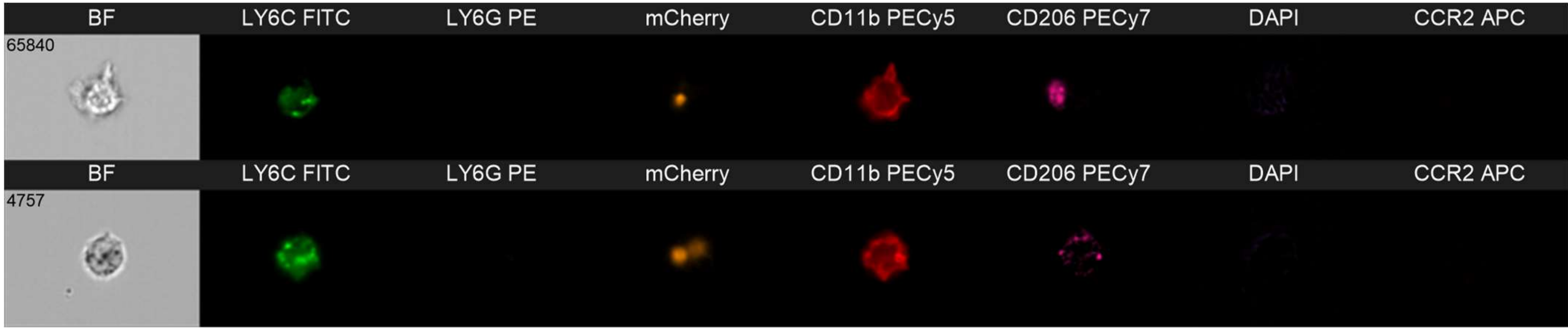

Panel 1
(gated from TER119- CD45+)

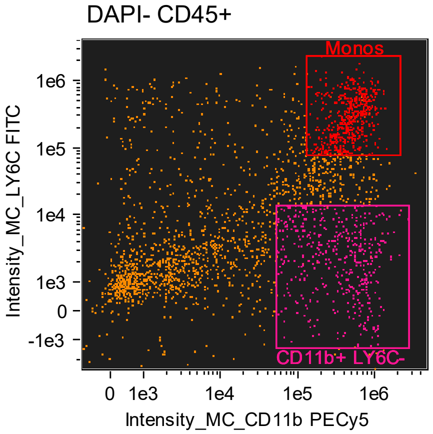

Intensity_MC_CD11b PECy5, Intensity_MC_LY6C FITC

Population	Count	%Gated
DAPI- CD45+ & single & CD45...	2511	100
Monos & DAPI- CD45+ & singl...	541	21.5
CD11b+ LY6C- & DAPI- CD45+ ...	464	18.5

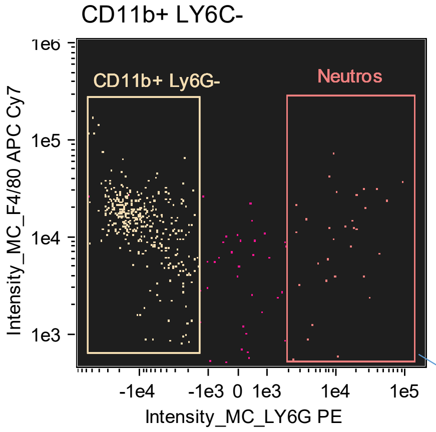

Intensity_MC_LY6G PE, Intensity_MC_F4/80 APC Cy7

Population	Count	%Gated
CD11b+ LY6C- & DAPI- CD45+ ...	464	100
CD11b+ Ly6G- & CD11b+ LY6C-...	375	80.8
Neutros & CD11b+ LY6C- & DA...	31	6.68

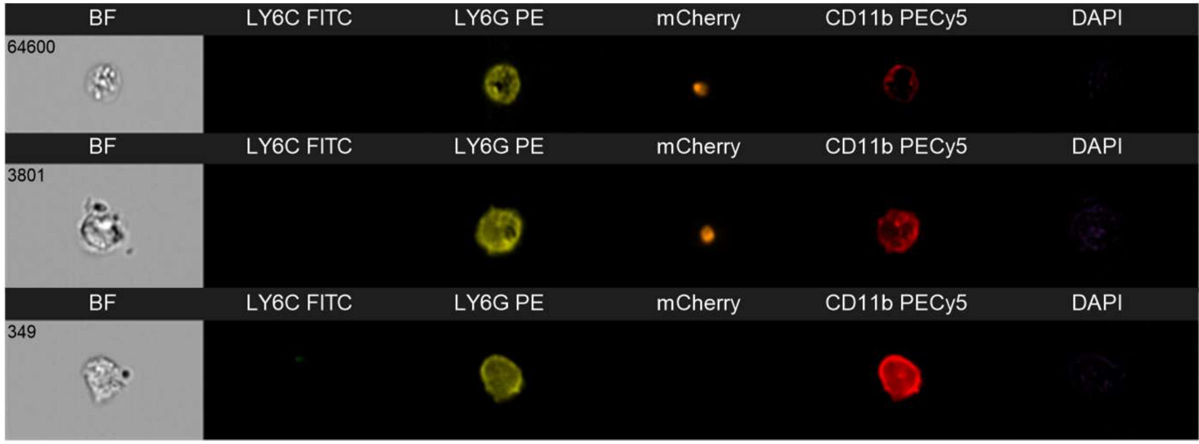

Panel 1

(gated from CD11b+ LY6G-)

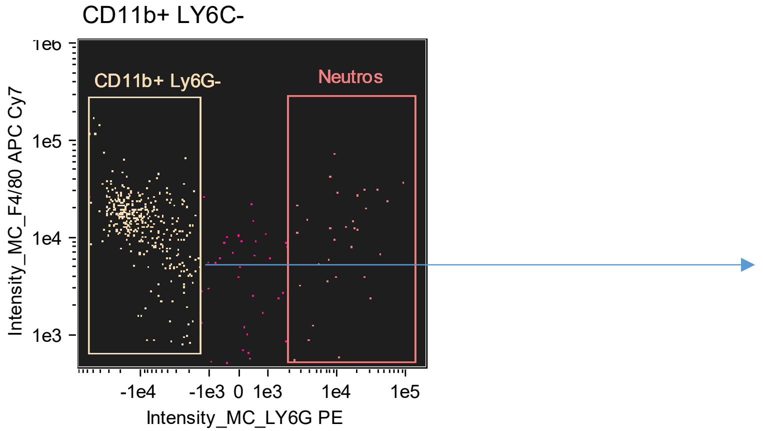

Intensity_MC_LY6G PE, Intensity_MC_F4/80 APC Cy7

Population	Count	%Gated
**CD11b+ LY6C- & DAPI- CD45+ ...**	464	100
**CD11b+ Ly6G- & CD11b+ LY6C-...**	375	80.8
**Neutros & CD11b+ LY6C- & DA...**	31	6.68

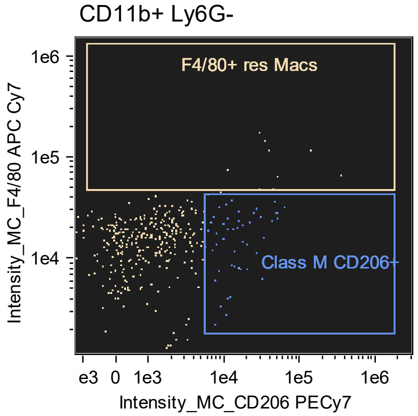

Intensity_MC_CD206 PECy7, Intensity_MC_F4/80 APC Cy7

Population	Count	%Gated
**CD11b+ Ly6G- & CD11b+ LY6C-...**	375	100
**F4/80+ res Macs & CD11b+ Ly...**	7	1.87
**Class M CD206+ & CD11b+ Ly6...**	50	13.3

Resident Macrophages (Ly6G-Ly6C-CD11b+ CD206+ F4/80+)

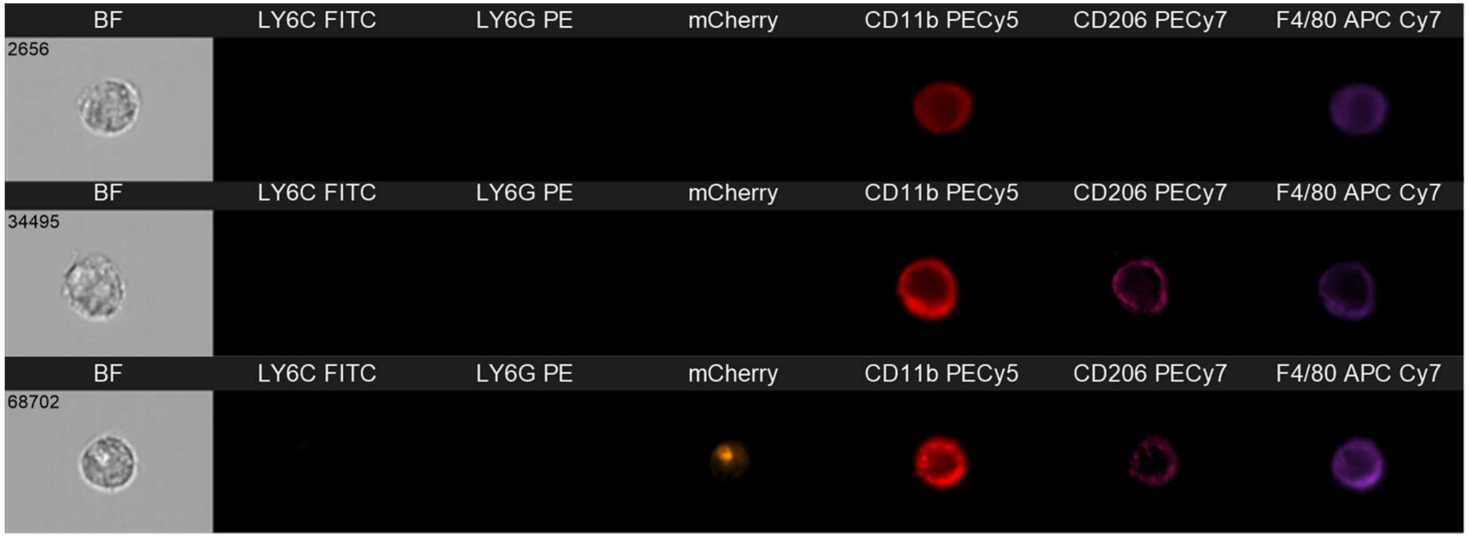

Resident Mannose receptor macrophages (Ly6G-Ly6C-CD11b+ CD206+ F4/80-)

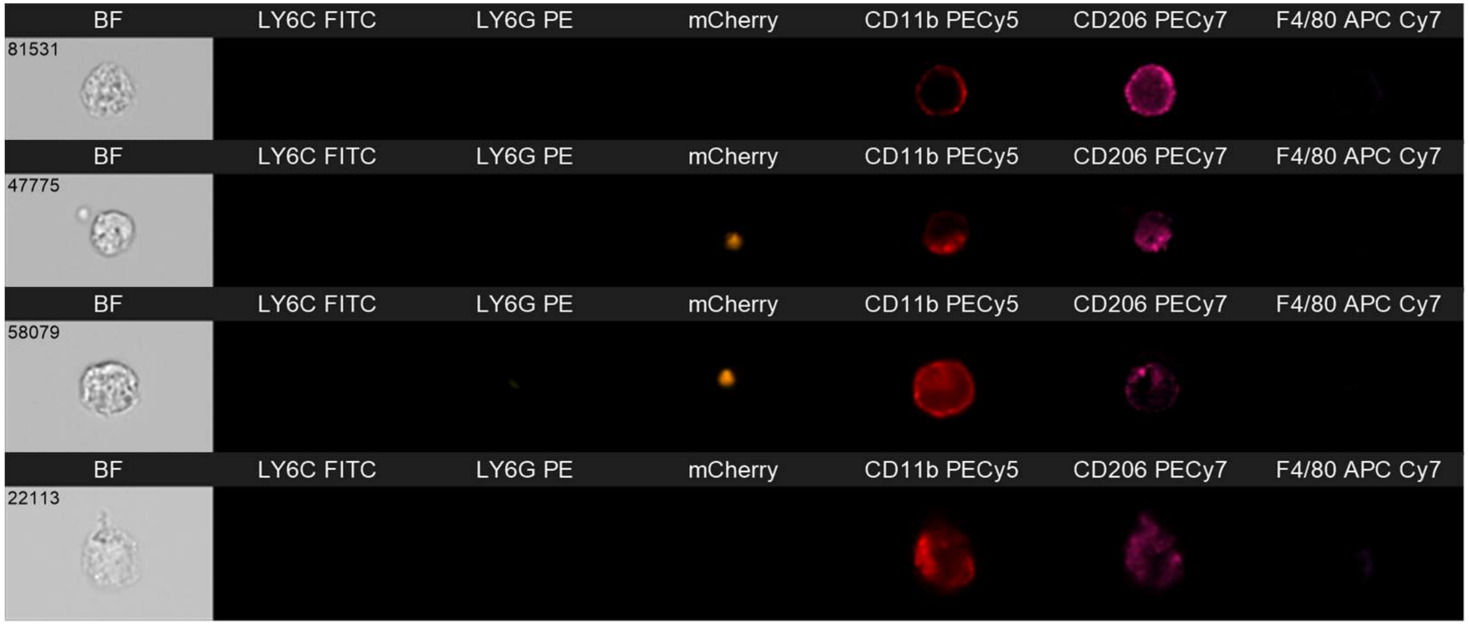

BF, single , simple mono , Infl monos CCR2+ , Class monos 206+ , F4/80+ res CD11b+ Ly6G- , Class M CD206+ , Neutros , mCherry +

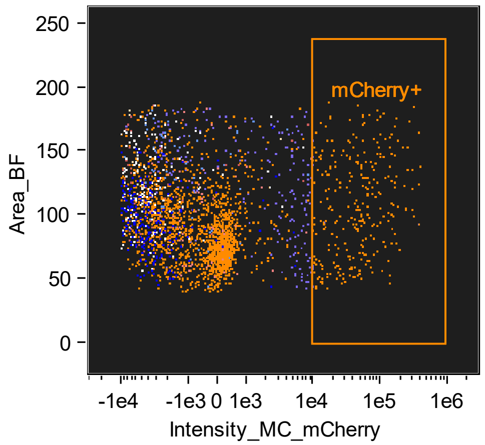

Intensity_MC_mCherry, Area_BF

Population	Count	%Gated
simple mono & Monos & DAPI-...	221	100
mCherry+ & simple mono & Mo...	19	8.6
Infl monos CCR2+ & Monos & ...	179	100
mCherry+ & Infl monos CCR2+...	18	10.1
Class monos 206+ & Monos & ...	65	100
mCherry+ & Class monos 206+...	16	24.6
F4/80+ res Macs & CD11b+ Ly...	8	100
mCherry+ & F4/80+ res Macs ...	1	12.5
Class M CD206+ & CD11b+ Ly6...	60	100
mCherry+ & Class M CD206+ &...	3	5
Neutros & CD11b+ LY6C- & DA...	63	100
mCherry+ & Neutros & CD11b+...	2	3.17
DAPI- CD45+ & single & CD45...	2511	100
mCherry+ & DAPI- CD45+ & si...	112	4.46
DAPI+ CD45+ & single & CD45...	379	100
mCherry+ & DAPI+ CD45+ & si...	169	44.6
