# Supplementary material for: In and out: Leishmania metastasis by hijacking lymphatic system and migrating immune cells
Source: Front Cell Infect Microbiol. 2022 Aug 12;12:941860. doi: 10.3389/fcimb.2022.941860 (PMC9414205; doi:10.3389/fcimb.2022.941860)

Supplementary Materials 2: Gating strategy for Panel 2 for FACS

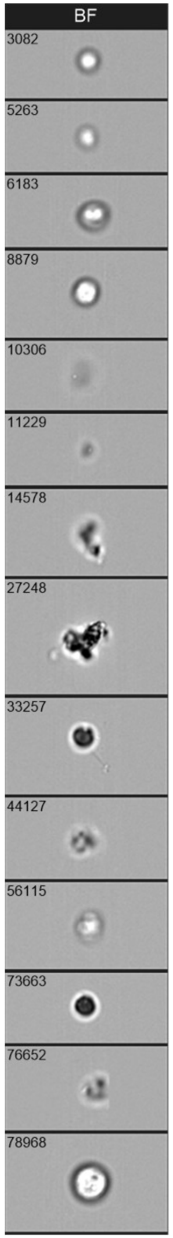

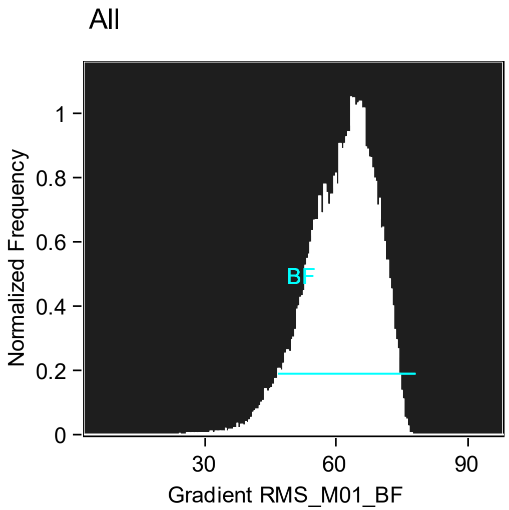

Gradient RMS_M01_BF

Population	Count	%Gated
All	100000	100
BF	95469	95.5

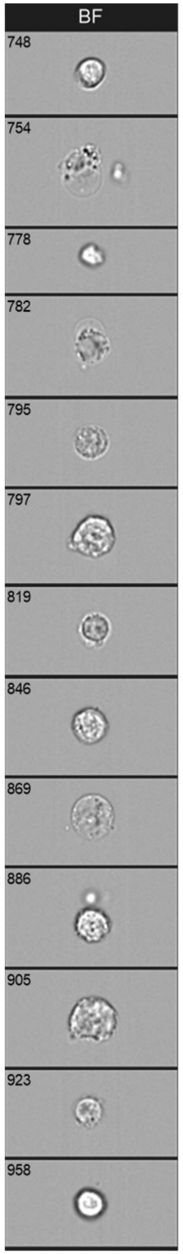

	Ch01	Ch02	Ch03	Ch04	Ch05	Ch06	Ch07	Ch08	Ch09	Ch10	Ch11	Ch12
Ch01	1	0.1	0.071	0.075	0.039	0.016	0.015	0	0	0	0.01	0
Ch02	0.042	1	0.633	0.087	0.05	0.043	0.102	0	0	0	0.059	0
Ch03	0	0.768	1	0.334	0.187	0.333	0.078	0	0.021	0	0.083	0
Ch04	0	0.68	0.544	1	0.095	0.158	0.07	0	0	0	0.044	0
Ch05	0	0.43	0.232	0.694	1	0.111	0.069	0.011	0	0	0.128	0
Ch06	0.013	0.178	0.083	0.135	0.139	1	0.02	0.001	0	0	0.022	0
Ch07	0.042	0.625	0.1	0.016	0.024	0.001	1	0.507	0.014	0	0.03	0
Ch08	0	0.514	0.085	0.017	0.023	0.001	0.432	1	0.018	0	0.029	0
Ch09	0	0.17	0.046	0.01	0.022	0.004	0.085	0.284	1	0	0.027	0
Ch10	0	0.138	0.031	0.021	0.017	0.002	0.051	0.192	0.029	1	0.022	0
Ch11	0	0.211	0.076	0.027	0.726	0.007	0.041	0.091	0.014	0	1	0
Ch12	0	0.084	0.02	0.007	0.106	0.133	0.045	0.057	0.013	0	0.152	1

Panel 2

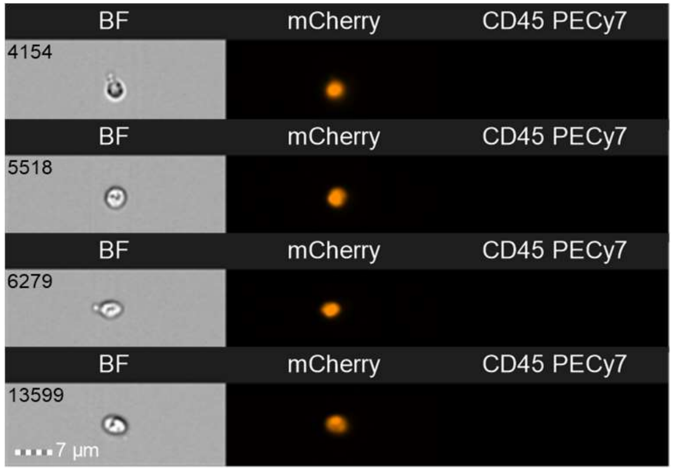

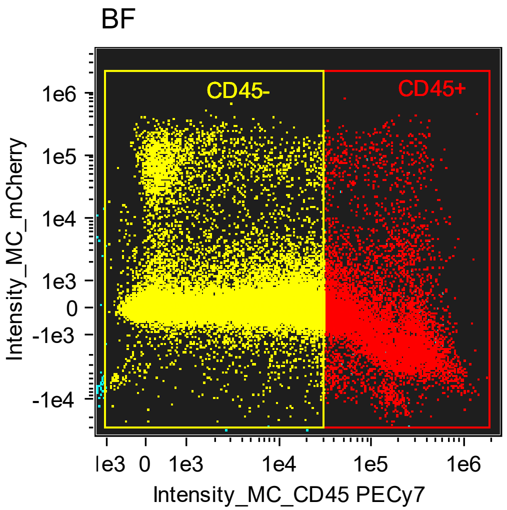

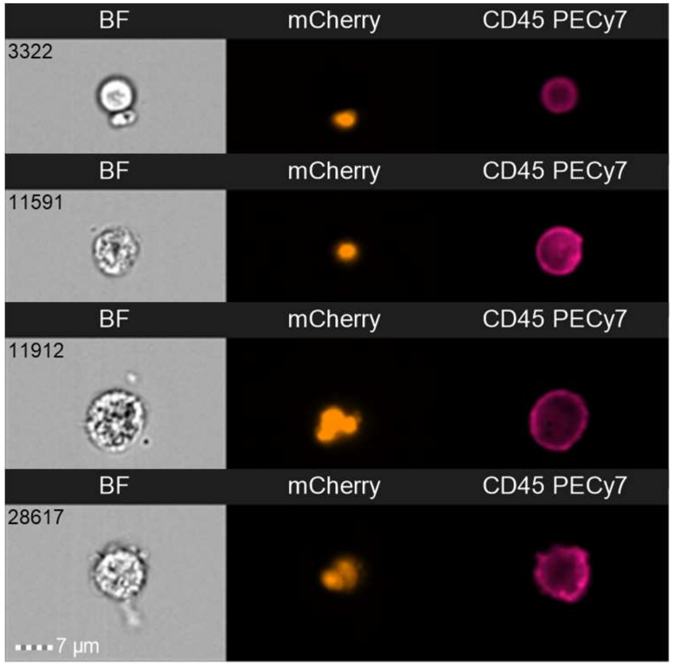

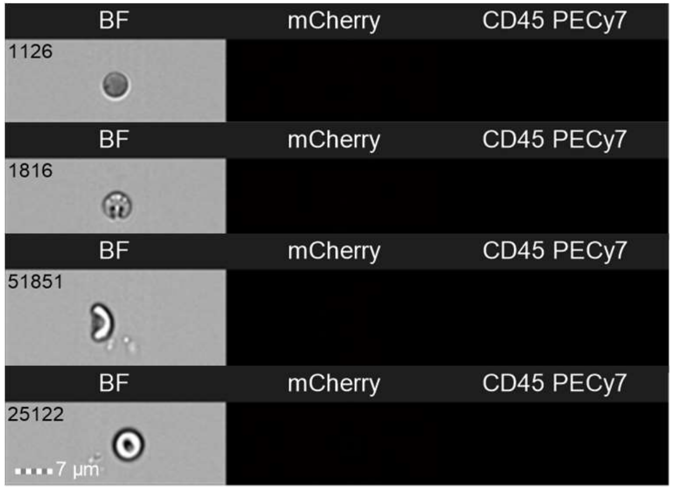

Intensity_MC_CD45 PECy7, Intensity_MC_mCherry

Population	Count	%Gated
BF	95469	100
CD45+ & BF	10246	10.7
CD45- & BF	85042	89.1

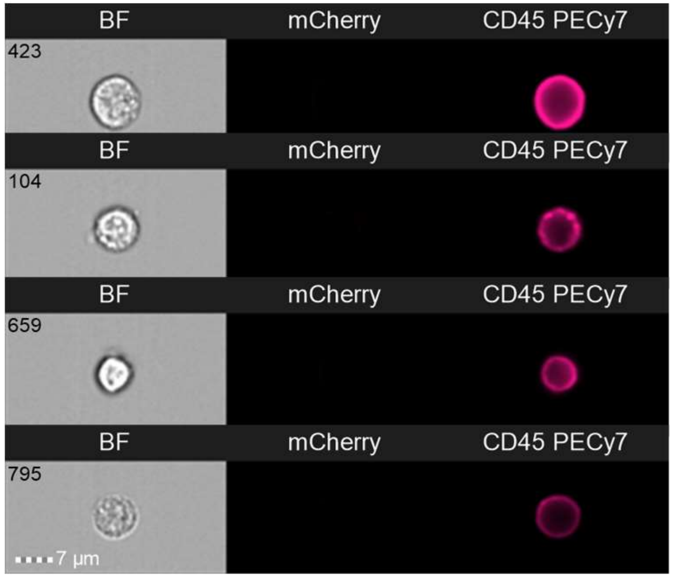

Panel 2

(TER119-CD45- free parasites)

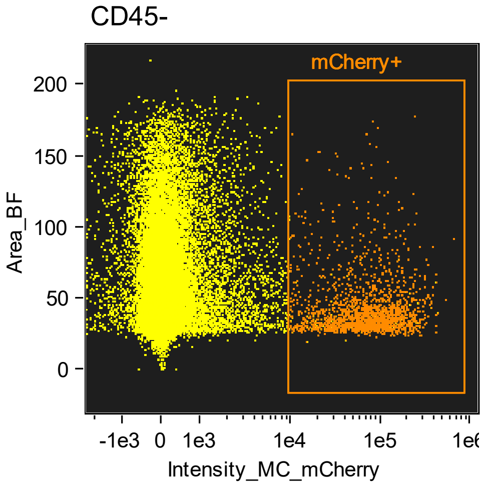

Intensity_MC_mCherry, Area_BF

Population	Count	%Gated
**CD45- & BF**	85042	100
**mCherry+ & CD45- & BF**	1930	2.27

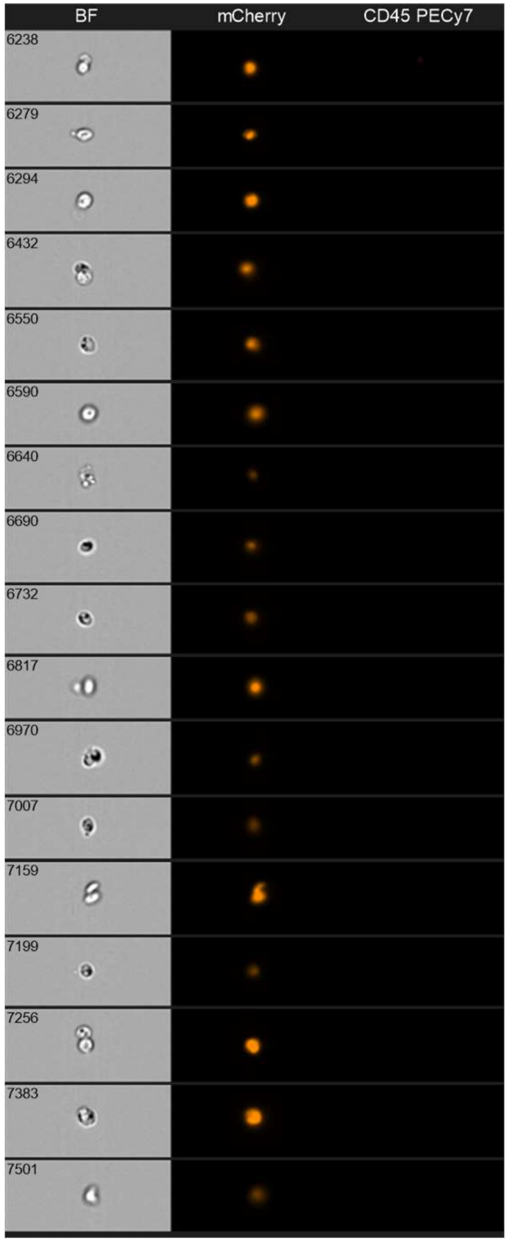

Panel 2

(gated from TER19-CD45+)

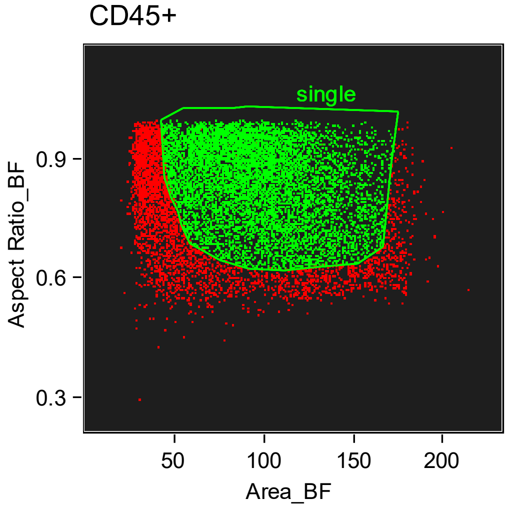

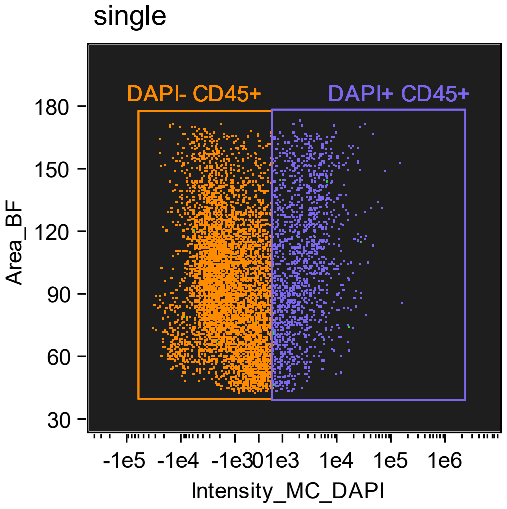

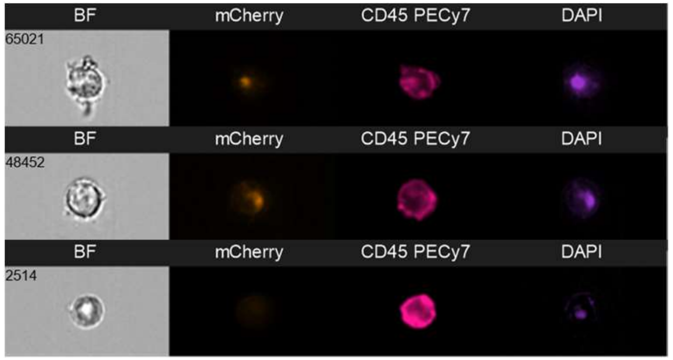

Area_BF, Aspect Ratio_BF

Population	Count	%Gated
CD45+ & BF	10246	100
single & CD45+ & BF	6982	68.1

Intensity_MC_DAPI, Area_BF

Population	Count	%Gated
single & CD45+ & BF	6982	100
DAPI- CD45+ & single & CD45...	5611	80.4
DAPI+ CD45+ & single & CD45...	1367	19.6

Panel 2: B cells, T cells, DCs

DAPI- CD45+

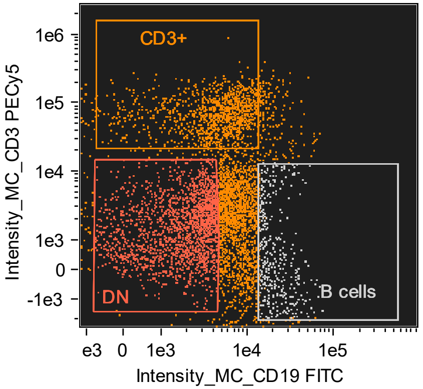

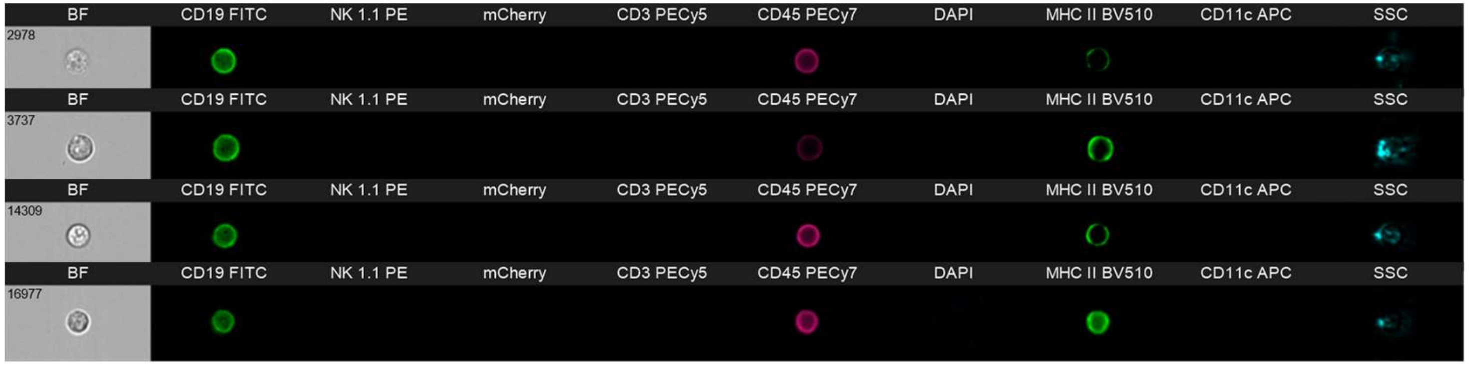

Intensity_MC_CD19 FITC, Intensity_MC_CD3 PECy5

Population	Count	%Gated
DAPI- CD45+ & single & CD45...	5611	100
CD3+ & DAPI- CD45+ & single...	1113	19.8
DN & DAPI- CD45+ & single &...	1971	35.1
B cells & DAPI- CD45+ & sin...	379	6.75

DN

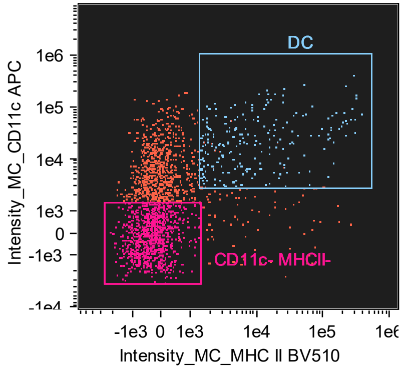

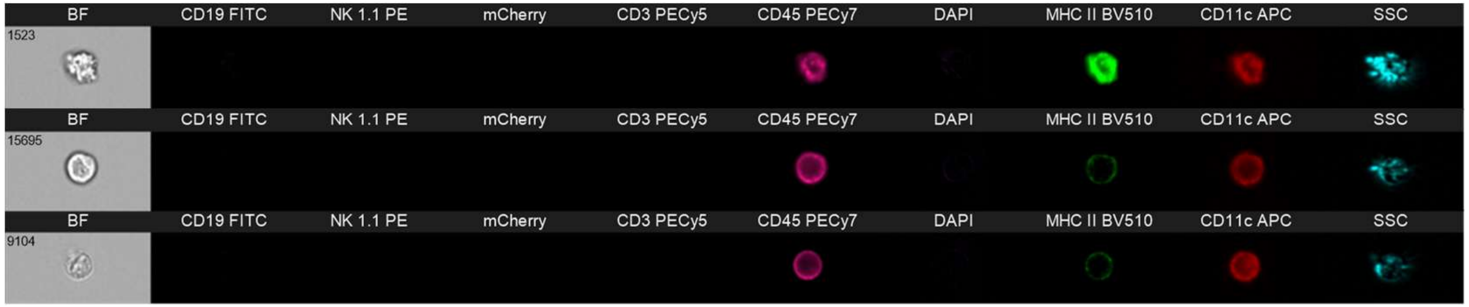

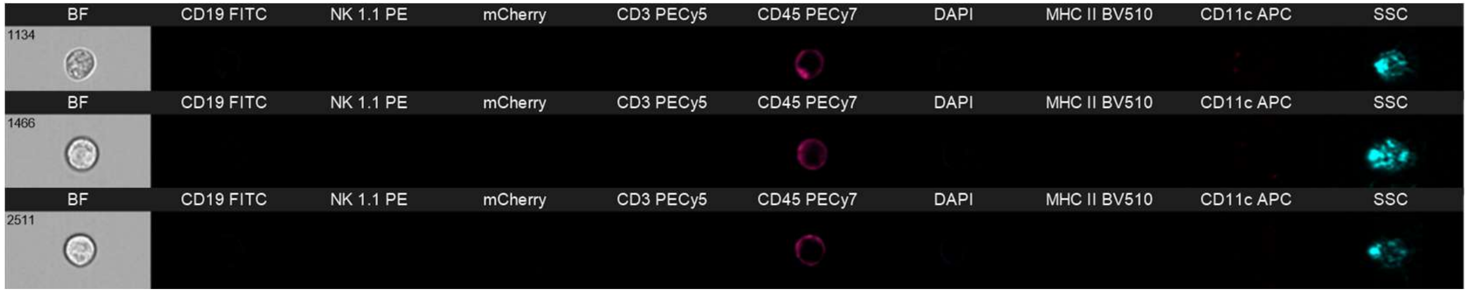

Intensity_MC_MHC II BV510, Intensity_MC_CD11c APC

Population	Count	%Gated
DN & DAPI- CD45+ & single &...	1971	100
DC & DN & DAPI- CD45+ & sin...	226	11.5
CD11c- MHCII- & DN & DAPI- ...	918	46.6

Panel 2: B cells, T cells, DCs

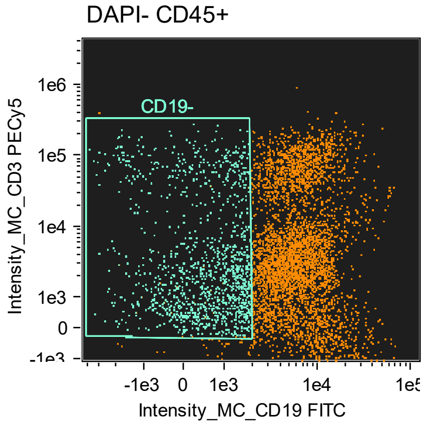

Intensity_MC_CD19 FITC, Intensity_MC_CD3 PECy5

Population	Count	%Gated
DAPI- CD45+ & single & CD45...	5611	100
CD19- & DAPI- CD45+ & singl...	1274	22.7

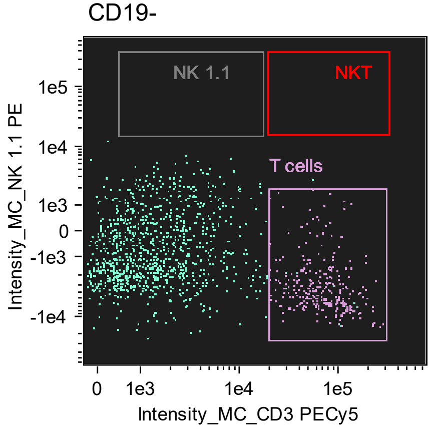

Intensity_MC_CD3 PECy5, Intensity_MC_NK 1.1 PE

Population	Count	%Gated
CD19- & DAPI- CD45+ & singl...	1274	100
NKT & CD19- & DAPI- CD45+ &...	0	0
NK 1.1 & CD19- & DAPI- CD45...	0	0
T cells & CD19- & DAPI- CD4...	290	22.8

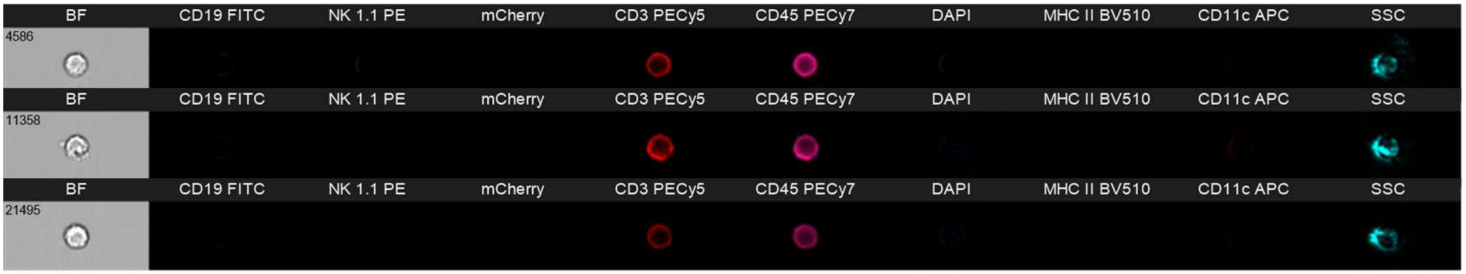

Panel 2: mCherry+ in the B cells, T cells, DCs:

CD45+ , single , DAPI- CD45+ , s , DC , CD11c- MHCII- , CD19- , NKCD3+ , NK 1.1 , CD3+ cells , DAPI+ CD45+ , CD45-

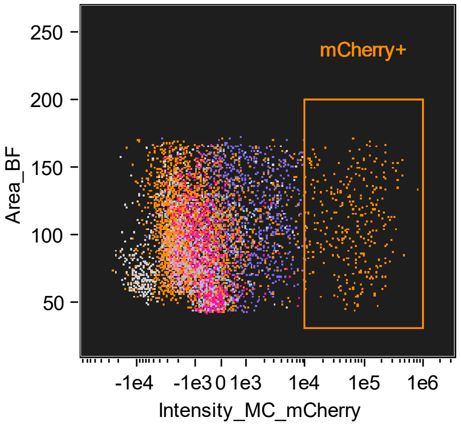

Intensity_MC_mCherry, Area_BF

Population	Count	%Gated
DC & DN & DAPI- CD45+ & sin...	226	100
mCherry+ & DC & DN & DAPI- ...	8	3.54
T cells & CD19- & DAPI- CD4...	318	100
mCherry+ & T cells & CD19- ...	2	0.63
CD11c- MHCII- & DN & DAPI- ...	918	100
mCherry+ & CD11c- MHCII- & ...	15	1.63
B cells & DAPI- CD45+ & sin...	379	100
mCherry+ & B cells & DAPI- ...	21	5.54
DAPI- CD45+ & single & CD45...	5611	100
mCherry+ & DAPI- CD45+ & si...	177	3.15
DAPI+ CD45+ & single & CD45...	1367	100
mCherry+ & DAPI+ CD45+ & si...	132	9.66
NKT & CD19- & DAPI- CD45+ &...	0	0
mCherry+ & NKT & CD19- & DA...	0	0
NK 1.1 & CD19- & DAPI- CD45...	0	0
mCherry+ & NK 1.1 & CD19- &...	0	0

CD45+ , single , DAPI- CD45+ , s , DC , CD11c- MHCII- , CD19- , NKCD3+ , NK 1.1 , CD3+ cells , DAPI+ CD45+ , CD45-

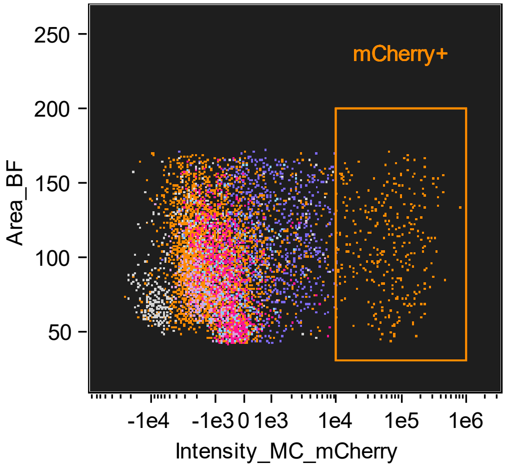

Intensity_MC_mCherry, Area_BF

Population	Count	%Gated
DC & DN & DAPI- CD45+ & sin...	226	100
mCherry+ & DC & DN & DAPI- ...	8	3.54
T cells & CD19- & DAPI- CD4...	318	100
mCherry+ & T cells & CD19- ...	2	0.63
CD11c- MHCII- & DN & DAPI- ...	918	100
mCherry+ & CD11c- MHCII- & ...	15	1.63
B cells & DAPI- CD45+ & sin...	379	100
mCherry+ & B cells & DAPI- ...	21	5.54
DAPI- CD45+ & single & CD45...	5611	100
mCherry+ & DAPI- CD45+ & si...	177	3.15
DAPI+ CD45+ & single & CD45...	1367	100
mCherry+ & DAPI+ CD45+ & si...	132	9.66

Panel 2: mCherry+ with B cells, T cells, DCs

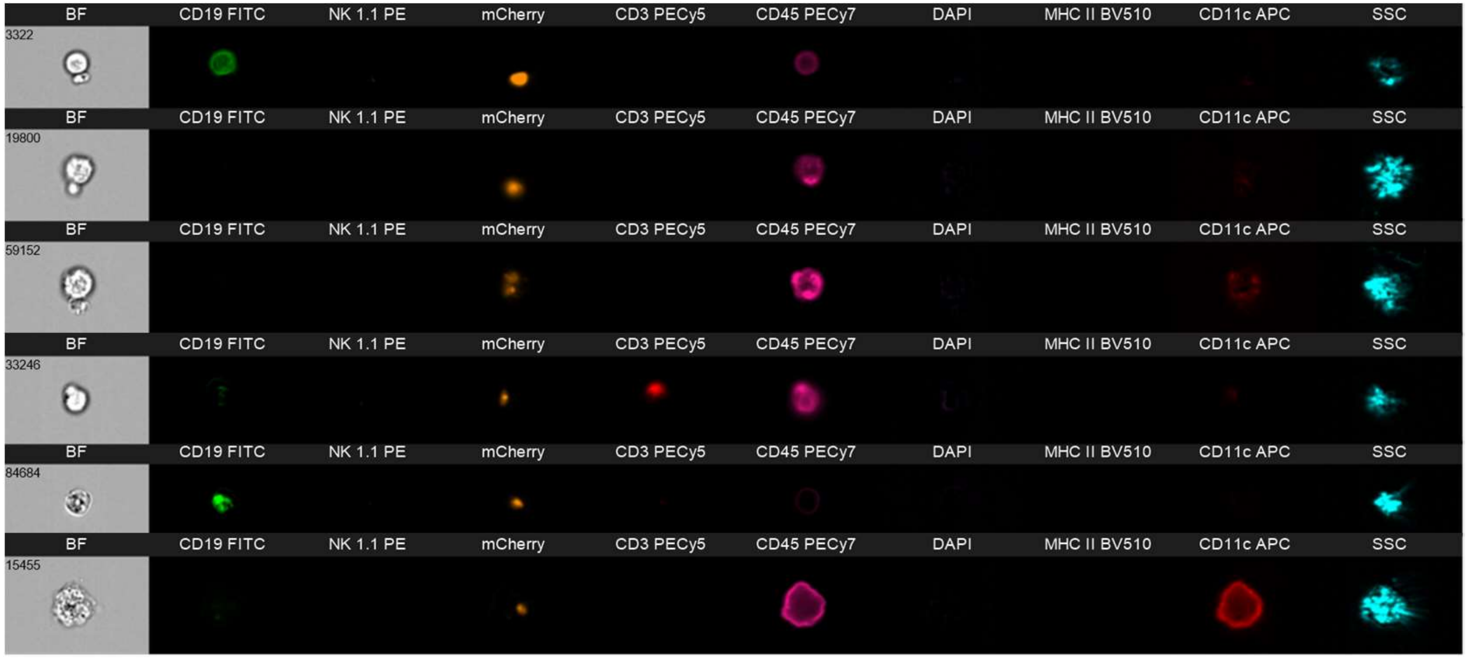

Supplement: Supplementary file 4 [file DataSheet_4.pdf]
